# Synthesis of Oxasilolanes by TBAT-Catalyzed Hydroxyl-Directed Hydrosilylation

**DOI:** 10.1021/acs.joc.5c01496

**Published:** 2025-08-26

**Authors:** Tess Q. Billmire, Adam P. Jones, Sarah M. Maffett, Robert F. Berger, Claire Gervais, Werner Kaminsky, Gregory W. O’Neil

**Affiliations:** † Department of Chemistry, 1632 Western Washington University, Bellingham, Washington 98229, United States; ‡ Department of Chemistry, 7284 University of Washington, Seattle, Washington 98195, United States

## Abstract

The reaction of allylic and homoallylic styrenyl alcohols with diphenylsilane and catalytic tetrabutylammonium difluorotriphenylsilicate (TBAT) produces 5- and 6-membered ring oxasilolanes, respectively. Differing substitution at the carbinol position, phenyl ring, and carbon–carbon double bond were all found to have significant impacts on both yield and diastereoselectivity. A mechanism is proposed involving fluoride-promoted intramolecular hydrosilylation and formation of an intermediate benzylic anion, followed by cyclization and oxasilolane formation. This mechanism is supported by double bond stereospecificity experiments along with the scope and stereochemical outcome of the reaction.

## Graphical abstract

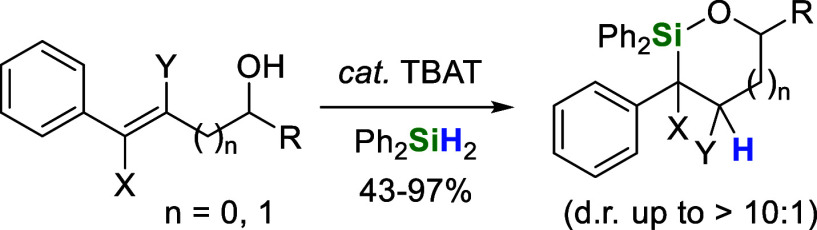

## Introduction

The hydrosilylation reaction is a fundamental process in organosilicon chemistry, widely implemented to produce valuable organosilanes, for instance as intermediates in the synthesis of pharmaceuticals and polymers.^1^ The reaction involves the addition of silicon and hydrogen (Si–H) across a π bond of the substrate, enabling the introduction of silicon into molecular structures for the development of advanced materials with enhanced properties. The history of the hydrosilylation reaction dates back to the 1940s,^2^ followed by a seminal paper in 1957 by Speier^3^ describing the use of chloroplatinic acid (H_2_PtCl_6_) as a catalyst to promote the addition of Si–H bonds to alkenes. Karstedt’s catalyst, a platinum(0)-divinyltetramethyldisiloxane complex, was introduced in the 1970s,^4^ offering improved activity and selectivity compared to Speier’s catalyst and became a standard for industrial processes.^5^ In the following decades, other transition metals such as rhodium, ruthenium, and palladium were introduced as hydrosilylation catalysts.^6^ Modern hydrosilylation research has focused on earth-abundant metal catalysis (iron, cobalt, nickel) to reduce reliance on expensive precious metals,^1c,7^ and green chemistry approaches like solvent-free conditions^8^ and recyclable catalysts.^9^ Hydrosilylation continues to be a powerful tool in synthetic chemistry, with ongoing innovations improving its sustainability, selectivity, and scope.^10^

Recently, our group reported a tetrabutylammonium difluorotriphenylsilicate (TBAT)-catalyzed hydrosilylation of beta-hydroxy ketones (Scheme 1).^11^ The reaction is thought to proceed by intramolecular hydrosilylation through intermediate silicate **Si–I**. As a result, dioxasilinane products can be obtained with high levels of chemoselectivity and diastereoselectivity. In the interest of expanding the scope of this reaction to include other types of substrates, we discovered that the same reagent combination (i.e., TBAT + diphenylsilane (Ph_2_SiH_2_)) was capable of transforming styryl alcohol **1** into oxasilolane **2**.^12^ Transformations of this type are generally carried out using expensive transition metals.^13–15^ In fact, the conversion of **1** into **2** had previously been reported by Gatineau and co-workers in 2013,^16^ but in that case using a two-step sequence involving first silyl ether formation followed by treatment with Wilkinson’s catalyst (Rh­(PPh_3_)_3_Cl). A comparison of the NMR data we obtained for **2** to that reported by Gatineau revealed the same sense of diastereoselection for the two methods. While the yield from our TBAT-catalyzed reaction was lower, we thought this strategy warranted further investigation given the value of oxasilolanes as synthetic intermediates^17–21^ and to compliment other efforts aimed at replacing precious metals in hydrosilylation chemistry.^1c,22^ Moreover, with growing interest in silicon-containing pharmaceuticals,^23^ oxasilolanes may emerge as a valuable heterocycle for medicinal chemistry studies.

**Scheme 1:**
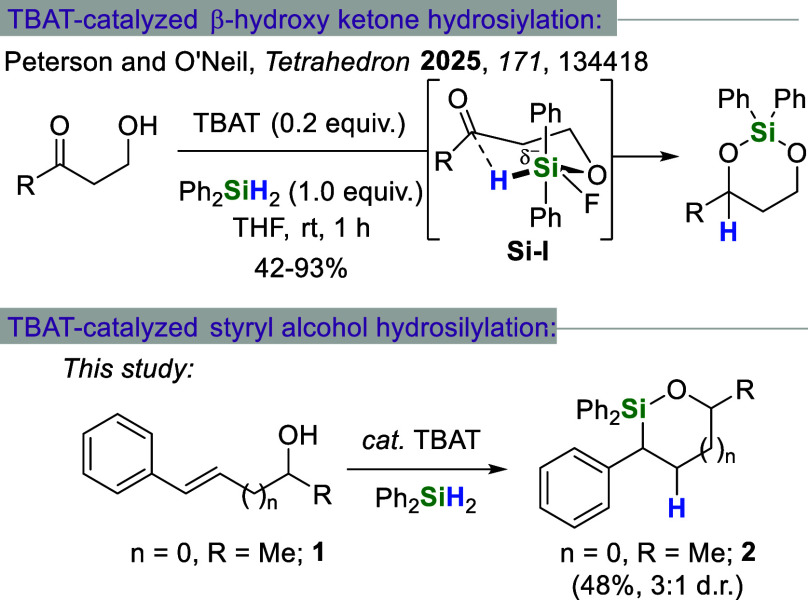
Previously Reported TBAT-Catalyzed Beta-Hydroxy Ketone Hydrosilylation (Top) and TBAT-Catalyzed Styryl Alcohol Hydrosilylation (Bottom) Described in This Paper

## Results and Discussion

Our studies began with the synthesis of styryl alcohols **3**–**6** ^24,25^ featuring different carbinol groups (R) and investigating their TBAT-promoted hydrosilylation (Table 1). Oxasilolane products were obtained in good yield (83–96%) from compounds **3**–**5** upon treatment with catalytic TBAT (0.2 equiv) and stoichiometric Ph_2_SiH_2_ in toluene (PhMe) at room temperature (Entries 2–4). The lower yield from **1** can be explained by competing dimerization/oligomerization, evidenced by persistent signals in the alkene region of the ^1^H NMR spectrum for the crude product mixture, presumably facilitated by having a smaller methyl group flanking the hydroxyl. This could also explain why a complex mixture was obtained from cinnamyl alcohol (Entry 5). For compound **6** (R = Ph), a complex mixture was obtained for reasons that are not yet understood but may arise from the alcohol being both allylic and benzylic (Entry 6). Diastereoselectivity ranged from 3.3:1 to 5.6:1 and increased with increasing sterics of the carbinol group. Stereochemistry for the major diastereomer of products is assumed to be the same as that obtained for compound **2**.

**Table 1:**
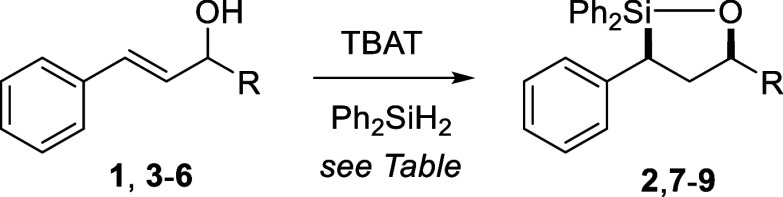
TBAT-Catalyzed Hydrosilylation of Styryl Alcohols Differentiated at the Carbinol Position^a^

entry	R	yield (%)^b^	oxasilolane (d.r.)^c^
1	Me (**1**)	48	**2** (3.3:1)
2	*n*-Bu (**3**)	84	**7** (3.4:1)
3	*i*-Pr (**4**)	83	**8** (4.9:1)
4	*t*-Bu (**5**)	75	**9** (5.6:1)
5	H		
6	Ph (**6**)		
7	Me (**1**)	55	**2** (3.4:1)
8	*n*-Bu (**3**)^d^	81	**7** (3.1:1)
9	*t*-Bu (**5**)^d^	87	**9** (4.5:1)
10	Me (**1**)^e^	30	**2** (3.7:1)
11	*i*-Pr (**4**)^f^	31	**8** (3.7:1)
12	*i*-Pr (**4**)^g^	12^l^	**8** (ND)
13	*i*-Pr (**4**)^h^	6^l^	**8** (ND)
14	*i*-Pr (**4**)^i^	42^l^	**8** (2.8:1)
15	*i*-Pr (**4**)^j^		
16	*i*-Pr (**4**)^k^		

a Notes for Table: Reactions were performed by adding TBAT (0.2 equiv) to a solution of the substrate and Ph_2_SiH_2_ (0.9 equiv) in PhMe at room temperature followed by stirring for 15 h.

b Isolated yield after chromatography on silica.

c Determined by ^1^H NMR.

d Performed at 50 °C for 2 h.

e Performed at 0 °C for 24 h.

f Performed using dichloromethane as solvent.

g Performed using tetrahydrofuran as solvent.

h Performed using acetonitrile as solvent.

i Performed using DMSO as solvent.

j Performed using TBAF in place of TBAT.

k Performed using Et_2_SiH_2_ in place of Ph_2_SiH_2_. ND = Not Determined.

l Determined by ^1^H NMR using *p*-xylene as an internal standard.

Performing the reaction at slightly elevated temperature (50 °C) resulted in a more efficient reaction with no noticeable change in yield or diastereoselectivity (Entries 7–9). Reducing the reaction temperature to 0 °C gave a lower yield of the oxasilolane product even with extended reaction time (Entry 10). Using compound **4** we also screened several different solvents (Entries 11–14). As shown in Table 2, while oxasilolane was formed during reactions with each of the solvents tested, toluene proved optimal. Replacing TBAT with tetrabutylammonium fluoride (TBAF) gave no oxasilolane (Entry 15). Instead, the reduced saturated product was formed that we attribute to the presence of adventitious moisture often associated with this reagent.^26^ The use of diethylsilane in place of diphenylsilane also failed to generate the oxasilolane, with no hydrosilylation occurring by ^1^H NMR analysis (Entry 16).

**Table 2:**
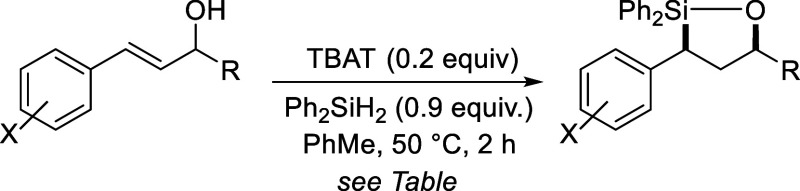
Phenyl Substituent Effects^a^

entry	X	R	% yield^b^	d.r.^c^
1	*p*-F	Bu	60	1.8:1
2	*p*-Cl	*i*-Pr	56	3.7:1
3	*p*-Br	*i*-Pr	79	5.5:1
4	*p*-Me	Bu	90	2.0:1
5	*p*-OMe	Bu	70	1.9:1
6	*m*-Me	Bu	66	3.1:1
7	*o*-Br	*i*-Pr		
8	*o*-OMe	Bu	43	2.2:1

a Notes for Table: Reactions were performed by adding TBAT (0.2 equiv) to a solution of the substrate and Ph_2_SiH_2_ (0.9 equiv) in PhMe followed by stirring 50 °C for 2 h.

b Isolated yield after chromatography on silica.

c Determined by ^1^H NMR.

Using the optimum conditions identified in Table 1, several additional styryl alcohols were examined to further investigate the substrate scope (Table 2). Selection of substrates for this study was based in part on the commercial availability of the starting cinnamyl aldehydes from which the substrates were readily prepared by addition of isopropylmagnesium bromide (*i*-PrMgBr) or butyllithium (BuLi), as well as an interest in testing different substituent types (e.g., electron-donating vs electron-withdrawing) and locations on the benzene ring. Some interesting patterns emerged from these results. For instance, an ortho-substituent seems to be deactivating, presumably as a result of sterics, with no oxasilolane product formed when an ortho-bromine was present and a lower yield (43%) when the ortho-substituent was a methoxy group (Entries 7 and 8 respectively). For reasons that are not yet understood, *para*-chloro (*p*-Cl, Entry 1) and *para*-fluoro (*p*-F; Entry 2) substitutions resulted in noticeably lower yields of the oxasilolane product compared to the other para-substituents tested (Br, Me, and OMe; Entries 3–5). From the ^1^H NMR spectrum of the crude product mixtures from the *p*-Cl and *p*-F reactions, no alkene signals remained, suggesting that hydrosilylation did occur, but other products than the oxasilolane were formed. Stereochemistry of the major diastereomer is assumed to be as drawn, in line with our results from Table 1.

We also tested the reaction on trisubstituted alkene substrates (Scheme 2). In this case, slightly higher reaction temperatures were required (80 °C). Additionally, it was found that only a methyl group at the carbinol position was tolerated. Within these constraints, polysubstituted oxasilolanes **10** and **11** could be generated, with the yield for **10** being significantly higher (96% vs 47% for **11**). In both cases, only a single diastereomer was observed by ^1^H NMR. The stereochemistry of **10** was assigned as illustrated based on the large coupling constants (*J*) measured for Ha-c in its ^1^H NMR spectrum, suggestive of an anti-arrangement for those hydrogens. This was confirmed by Tamao-oxidation^27^ of **10** which gave exclusively anti,anti- **12**, whose stereochemistry was confirmed by comparison to reported NMR data for the same compound.^28^ No evidence of additional diastereomers were observed in the ^1^H NMR spectrum of **12**, supporting the high diastereoselectivity of the hydrosilylation reaction and stereospecificity of the Tamao-oxidation. The stereochemistry of **11** was difficult to determine by NMR analysis and attempts at Tamao-oxidation of **11** for a similar analysis to **12** have thus far failed. Instead, protodesilylation of **11** was performed. Using TBAF for this purpose resulted in significant epimerization, giving **13** as a ∼1:1 mixture of diastereomers. Switching to *t*-BuOK and 18-crown-6 in DMSO,^29^ however, gave **13** as a single diastereomer by ^1^H NMR analysis whose stereochemistry could be assigned based on previously reported data.^30^ Assuming this was the result of a stereoretentive protodesilylation,^31^ the stereochemistry of **11** would be as drawn and consistent with the diastereoselectivity observed for the hydrosilylation reaction that produced oxasilolane **10**.

**Scheme 2:**
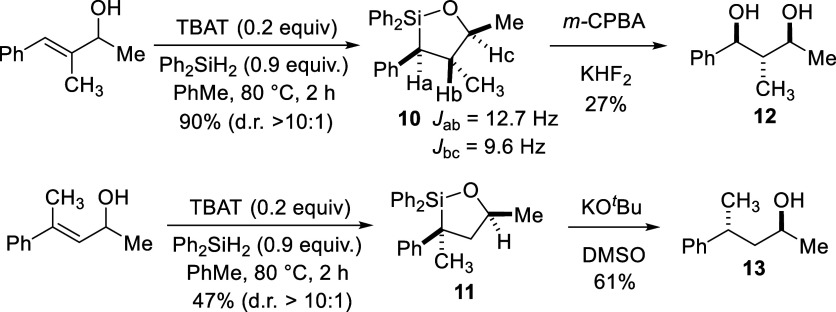
Formation of Oxasilolanes **10** and **11** by Hydrosilylation of Trisubstituted Alkene-Containing Styryl Alcohols and Stereochemistry Determination by ^1^H NMR Analysis and Conversion to Known Compounds **12** and **13**

Several experiments were conducted in an attempt to understand the mechanism of the reaction. For instance, the importance of the phenyl ring, carbon–carbon double bond, and hydroxyl group were examined using substrates **14**,^32^ **15**,^33^ and **16** ^34^ respectively (Scheme 3). With compounds **14**–**16**, no hydrosilylation products were observed by ^1^H NMR analysis. Using compound **17**,^35^ only hydrosilylation of the styrene C=C occurred, affording oxasilolane **18** in 97% yield.

**Scheme 3:**
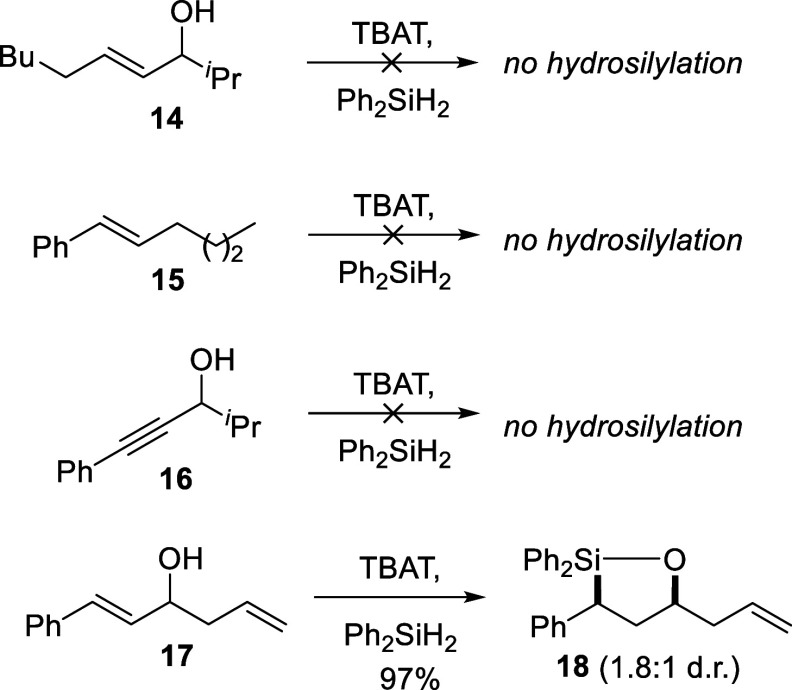
Testing the Importance of the Phenyl-, Hydroxyl, and C=C Groups

Compound **1** was also selectively synthesized as its cis-isomer (*Z-* **1**) and tested in a subsequent hydrosilylation reaction (Figure 1). Interestingly, the same major diastereomer of **2** was obtained as when the reaction was performed with *E*-**1**. However, the yield of **2** from *Z*-**1** was significantly lower (37% vs 55%). Together with the results presented in Scheme 4, we propose a three-step mechanism involving first fluoride-catalyzed silyl etherification^36^ and formation of **Si–I**. Intramolecular fluoride-catalyzed hydride addition^37^ generates carbanion **Si–II**, where now the starting C=C geometry is lost. Cyclization then furnishes the oxasilolane and regenerates the fluoride catalyst.

**Figure 1:**
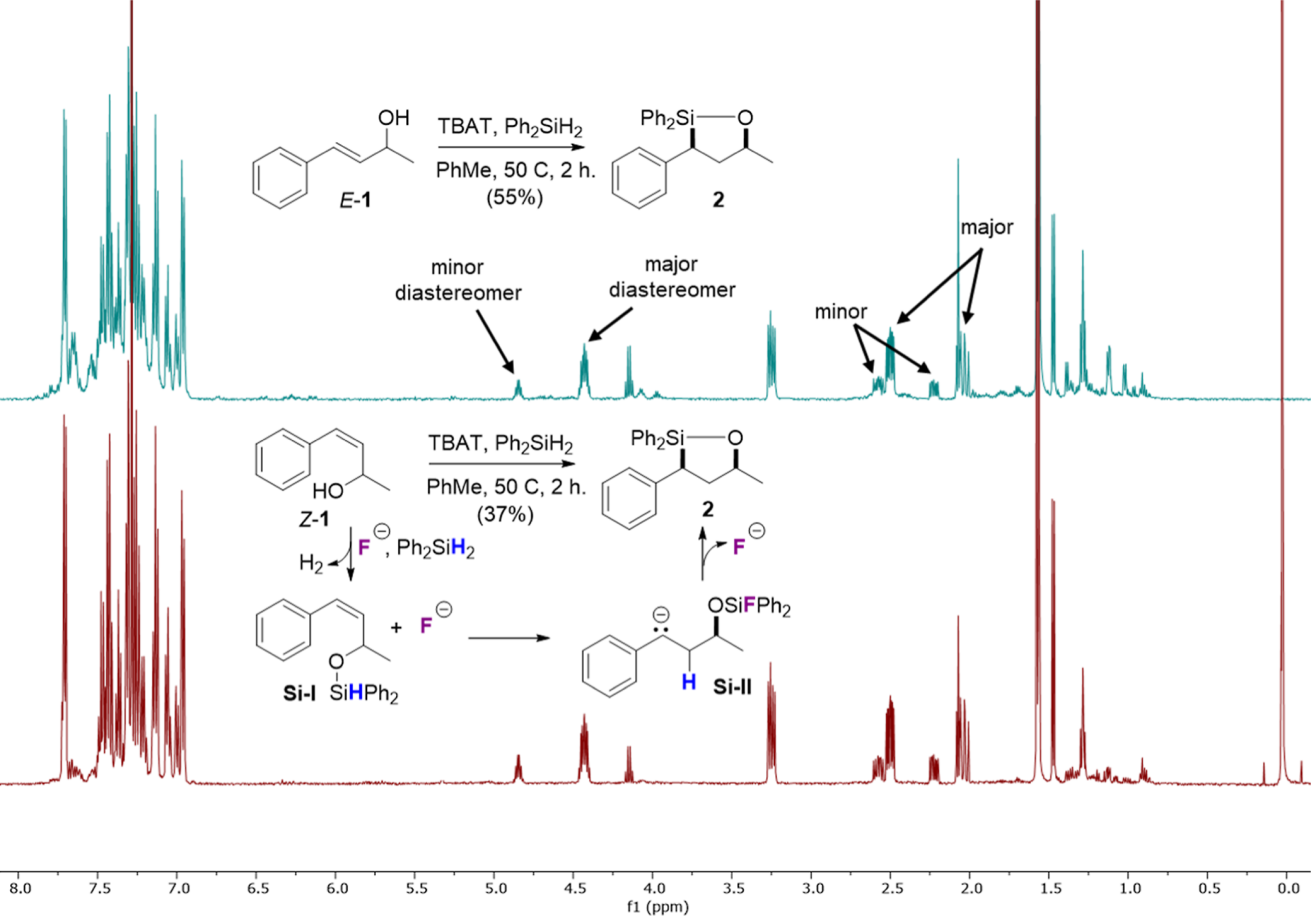
^1^H NMR spectra obtained from the hydrosilylation of *E*-**1** (top, blue trace) and *Z*-**1** (bottom, red trace). The isolated yield of **2** was lower from *Z*-**1**, yet the same major diastereomer was obtained. A mechanism is proposed to account for this result involving intermediates **Si–I** and **Si–II**.

**Scheme 4:**
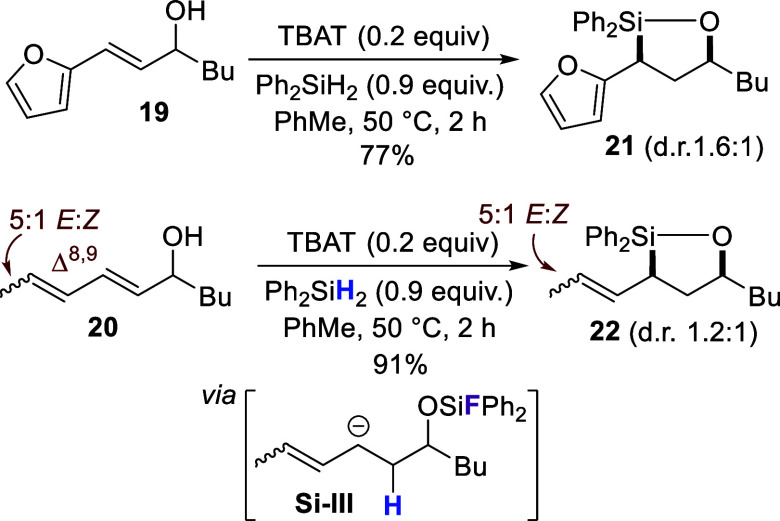
Oxasilolane Formation by Hydrosilylation of Furanyl- and Diene Alcohols **19** and **20**

The proposed mechanism would also explain the importance of the phenyl group by providing stabilization to the anionic intermediate **Si–II**. We questioned, therefore, if other groups capable of anion stabilization might also promote the reaction. To test this, furan **19** and diene **20** ^38^ were synthesized and used as substrates in subsequent hydrosilylations (Scheme 4). For both, the corresponding oxasilolanes **21** and **22** respectively were formed in good yield, supporting our hypothesis of a mechanism involving a resonance stabilized anionic intermediate **S–III**. Compound **20** used in this reaction was a 5:1 *E,E*:*E,Z* mixture, stemming from the 2,4-hexadienal that was purchased for its synthesis also being a 5:1 *E,E*:*E,Z* mixture.^39^ Interestingly, the stereochemistry of the Δ ^8,9^ double bond in compound **20** was retained upon conversion to **22**, suggesting that the carbanion in **Si–III** is perhaps localized at C7. The regioselectivity for the hydrosilylation of **20** is also noteworthy, where conjugated dienes represent a significant selectivity challenge for transition metal-catalyzed hydrosilylations.^40^

Six-membered ring oxasilolanes could also be formed with this procedure (Table 3). Similar to what was observed for the five-membered ring system, methyl and phenyl substitution at the carbinol position (R) was problematic (Entries 1 and 5). Otherwise, oxasilolanes **23–25** were obtained in good yield upon treatment of the corresponding homoallylic alcohols with Ph_2_SiH_2_ (0.9 equiv) and catalytic TBAT (0.2 equiv) in PhMe. Diastereoselectivity was modest for each of the substrates tested when performing the reaction at room temperature (Entries 2–5). Interestingly, increasing the reaction temperature resulted in an increase in d.r. (up to 9:1, Entry 9).

**Table 3:**
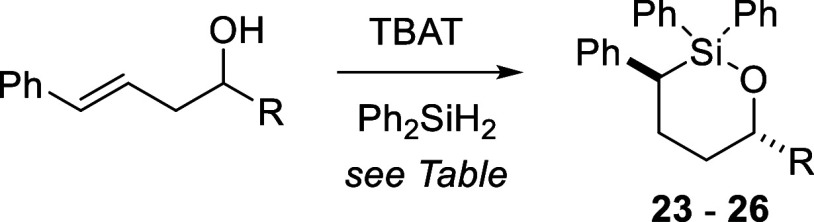
Six-Membered Ring Oxasilolane Synthesis^a^

entry	R	yield (%)^b^	d.r.^c^
1	Me^d^		
2	*n*-Bu^d^	71	**23** (1.8:1)
3	*i*-Pr^d^	74	**24** (1.2:1)
4	*t*-Bu^d^	94	**25** (1.3:1)
5	Ph^d^	37	**26** (1.4:1)
6	*n*-Bu^e^	91	**23** (5.1:1)
7	*n*-Bu^f^	59	**23** (6.3:1)
8	*i*-Pr^e^	88	**24** (11:1)
9	*i*-Pr^f^	97	**24** (9:1)

a Notes for Table: Reactions were performed by adding TBAT (0.2 equiv) to a solution of the substrate and Ph_2_SiH_2_ (0.9 equiv) in PhMe.

b Determined by ^1^H NMR using cis-butenediol as an internal standard.

c Determined by ^1^H NMR.

d Performed at room temperature for 15 h.

e Performed at 50 °C for 2 h.

f Performed at 80 °C for 2 h.

The stereochemistry of the major diastereomer of 6-membered oxasilolanes **23**–**25** was determined to be anti as drawn based on ^1^H NMR analysis (Figure 2). More specifically, the benzylic proton (**Hx**) of the major diastereomer in all cases displayed coupling constants consistent (*J*) with an axial orientation, whereas *J*-values for the minor diastereomer indicated an equatorial orientation. Ruling out the presumed high-energy anti-diaxial structure **D**, this data would then be consistent with structures **A** and **B** representing the major and minor diastereomers respectively. The major diastereomer of oxasilolane **25** also proved to be crystalline, and suitable crystals were able to be grown for analysis by X-ray diffraction. In line with our NMR analysis, the solved structure clearly shows the phenyl ring at C1 and *tert*-butyl group at C4 on opposite sides of the oxasilolane ring.

**Figure 2:**
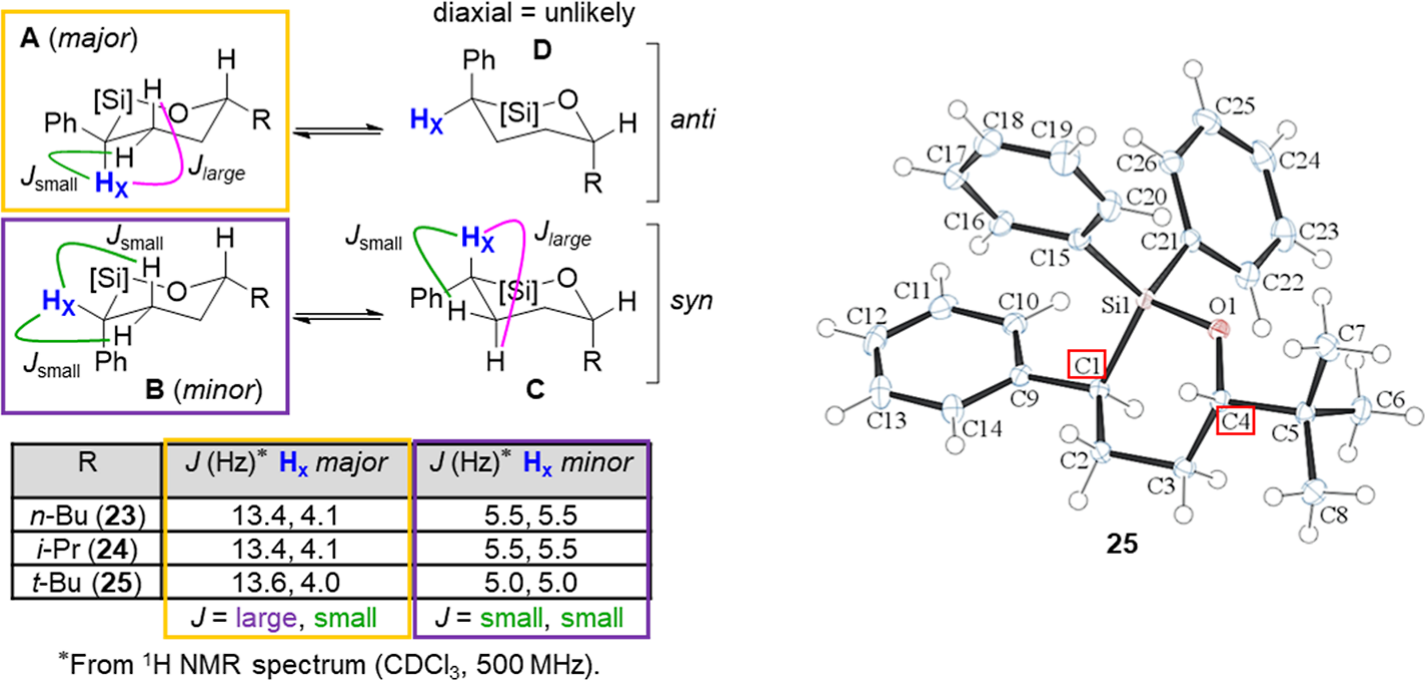
Stereochemical analysis of 6-membered oxasilolane products by NMR (left) and crystal structure ORTEP image of **25** with thermal elipsoids at the 50% probability level (right). Both sets of data indicate the major diastereomer obtained from hydrosilylation is the anti-substituted oxasilolane.

**Figure 3:**
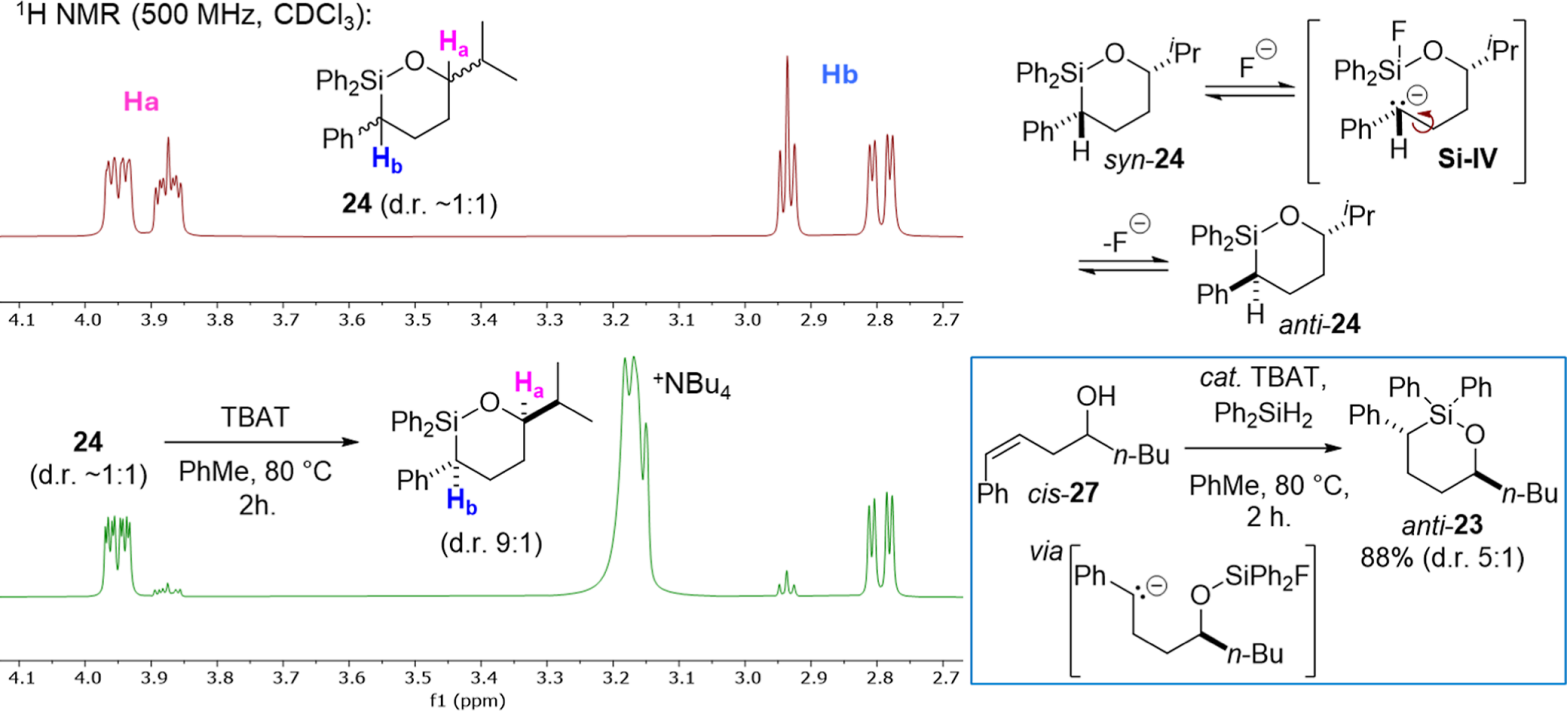
Stacked 1H NMR spectra of a 1:1 mixture diastereomeric of syn- and anti- **24** (top, red trace) and that obtained after treatment with TBAT at 80 °C for 2 h (bottom, green trace). The change in d.r., as indicated by signals for Ha (pink) and Hb (blue), indicates a possible ring-opening process involving benzyl carbanion **Si–IV** (shown to the right).

To better understand the stereochemistry of these hydrosilylation reactions, the relative energies of different six-membered oxasilolanes were computed by DFT (Table 4). In all cases, the anti-isomer was calculated to be lower in energy. This was true for oxasilinanes containing diphenyl- or dimethyl-substituted silicon atoms. As expected, larger groups like isopropyl and *tert*-butyl had a greater preference for the anti-isomer, which is consistent with the d.r. values obtained when synthesizing these oxasilolanes by hydrosilylation (ref. Table 3).

**Table 4:**
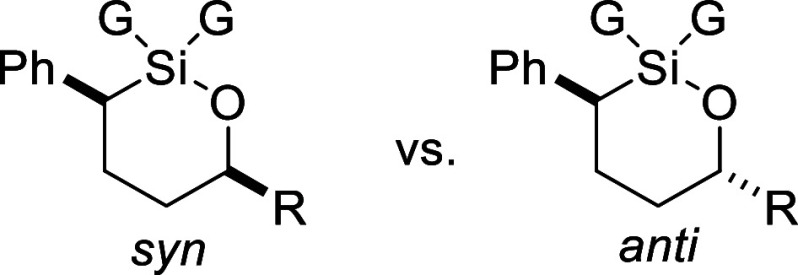
Computed Energy Differences by DFT Between syn- and anti-Substituted Oxasilinanes

G	R	Δ*E*_syn‑anti_ (kJ/mol)
Ph	Me	9.77
Ph	*n*-Bu	9.82
Ph	*i*-Pr	10.00
Ph	*t*-Bu	10.61
Me	Me	8.94
Me	*n*-Bu	8.45
Me	*i*-Pr	9.11
Me	*t*-Bu	9.17

Our DFT calculations suggest there could be a thermodynamic component to the stereochemical outcome of oxasilolane formation, which could explain the connection between higher temperatures and higher d.r. To test for reversibility, a ∼1:1 mixture of oxasilolane **24** was treated with TBAT in toluene at 80 °C (Figure 3). After 2 h, the d.r. changed to 9:1 in favor of the more stable anti-isomer. A potential mechanism for this isomerization would be fluoride-induced ring-opening of the syn-isomer and formation of benzylic anion **Si–IV**. Bond rotation followed by carbanion inversion and ring-closure would then give anti-**24**. Consistent with the intermediacy of benzylic carbanions such as **Si–IV** (and **Si–III**, *ref.* Figure 1), the reaction of cis-**27** produced primarily anti-**23**, the same major diastereomer obtained from the corresponding trans starting material (*ref*. Table 3, Entries 2, 6, and 7).

In line with the synthesis of 5-membered oxasilolanes, six-membered oxasilolane formation from alkyne **28** ^41^ under these conditions did not occur (Scheme 5). Similarly, attempted 7-membered oxasilolane formation from **29** also produced a complex mixture of products that we have tentatively identified as dimeric/oligomeric structures. Formation of a six-membered oxasilolane from a trisubstituted double bond was however successful. In this case, compounds **30** and **31** were transformed into oxasilolanes **32** and **33** respectively (Scheme 5). The large coupling constant in the ^1^H NMR spectrum for the protons attached to the phenyl- and methyl-substituted carbons in **32** suggests an anti-arrangement for these groups. The stereochemistry of **33** has yet to be confirmed but would be as drawn if the stereochemical trends observed for the substrates tested thus far hold for these systems as well. Like the 5-membered oxasilolanes, diastereoselectivity for 6-membered oxasilolane formation from trisubstituted double bonds exceeded those obtained when using disubstituted double bond-containing homoallylic alcohols. Both the yield and diastereoselectivity for **33** were higher than **32**. From the ^1^H NMR spectrum of the crude product mixture containing **32**, signals belonging to the double bond were no longer present, indicating that hydrosilylation had occurred, but oxasilolane formation was less selective than for **33**.

**Scheme 5:**
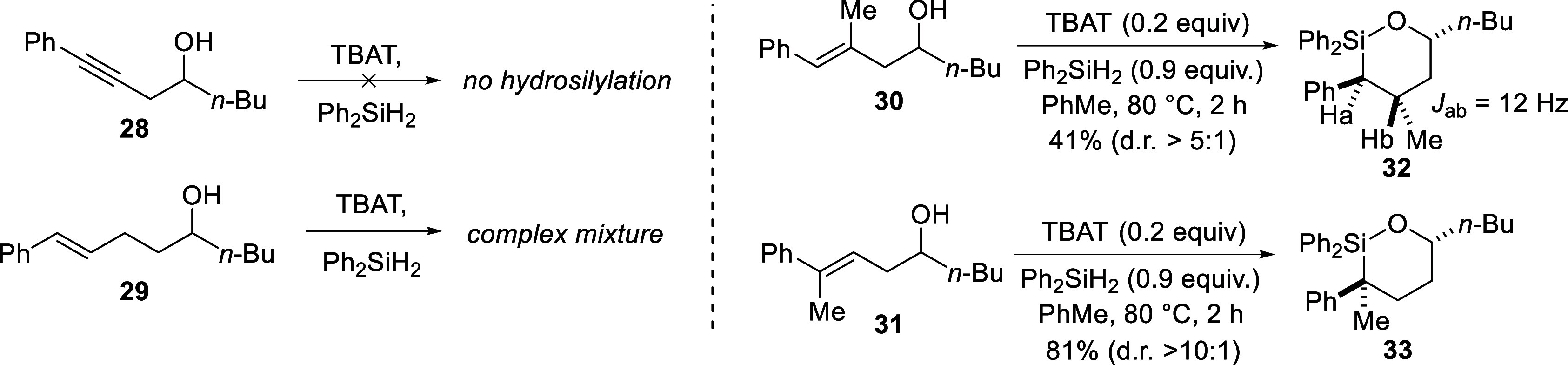
Failed Oxasilolane Formation and Hydrosilylation of Trisubstituted Double Bonds

Since these same conditions for C=C hydrosilylation were also effective for ketone hydrosilylations, we questioned if the two processes could be sequenced to create an atom-economical approach to oxasilolanes. As an initial test, commercially available enone **34** ^42^ was subjected to TBAT-catalyzed hydrosilylation conditions (Scheme 6). Oxasilolane **2** was indeed generated from this reaction, but in lower yield than what was obtained when using alcohol **1** (37% from **34** vs 55% from **1**). One potential cause for the lower yield of **2** from **34** would be a competing 1,4-hydrosilylation,^43–50^ leading to unwanted byproducts. Slowing down 1,4-addition, for instance by the introduction of larger groups at the 4-position, might therefore be one solution to overcome these difficulties. To test this, enone **35** featuring an additional methyl group at the 4-position was treated with diphenylsilane and catalytic TBAT in toluene at 80 °C. This reaction led to clean formation of oxasilolane **11** in comparable yield to what was obtained from the corresponding alcohol (52% vs 50%; *ref*. Scheme 3). The sense of diastereoselection was also the same, however the d.r. from the reaction with **35** was lower (3.5:1 vs 10:1).

**Scheme 6:**
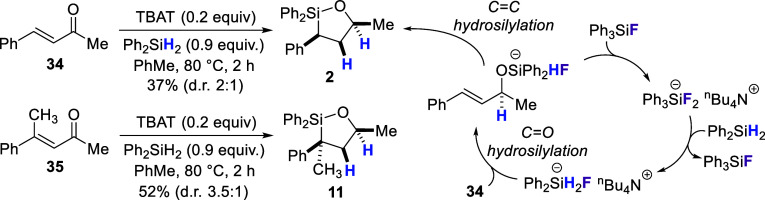
Oxasilolane Formation by Sequential C=O and C=C Hydrosilylation

We also used enone **34** to test the functional group tolerance of this tandem TBAT-catalyzed hydrosilylation reaction. According to the additive-based screening method of Glorius and co-workers,^57^ different additives featuring common functional groups (e.g., ester, amide, amine) were added to the reaction mixture and progress was monitored by ^1^H NMR. As shown in Table 5, yields of **2** were comparable for each of the additives tested with the exception of nitromethane (Entry 5). The ^1^H NMR spectrum from this reaction showed loss of signals corresponding to both **34** and nitromethane,^51^ indicating a reaction had occurred, but not formation of **2**. Base-promoted reduction of nitro groups by hydridosilanes is known,^52^ which may explain the loss of the nitromethane signal. However, the fate of **34** in this reaction has yet to be determined. Additive recoveries were also similarly high for all but nitromethane, suggesting these functional groups are compatible with this TBAT-catalyzed hydrosilylation methodology.

**Table 5:**
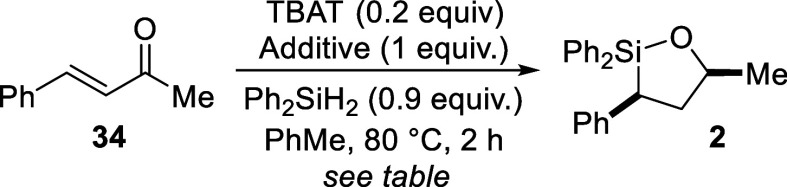
Functional Group Tolerance by Additive-Based Screening

entry	additive^a^	2 (yield %)^b^	additive recov. (%)^c^
1		36	
2	ethyl acetate	33	72
3	pyridine	35	75
4	acetonitrile	27	70
5	nitromethane	0	0
6	triethylamine	34	93

a Notes for table: All reactions were performed by adding TBAT (0.2 equiv) to a solution of **34** (250 mg) plus the indicated additive (1.0 equiv) and 1-octene (1.0 equiv) as an inert internal standard in toluene-d8 and heating to 80 °C for 2 h. Mixtures were analyzed directly by ^1^H NMR.

b Determined by comparing integration ratios in the ^1^H NMR spectrum for signals corresponding to **34**/1-octene before TBAT addition and **2**/1-octene after the reaction had occurred.

c Determined by comparing integration ratios in the ^1^H NMR spectrum for signals corresponding to the additive and 1-octene before TBAT addition and the additive and 1-octene after the reaction had occurred.

## Conclusion

Certain allylic- and homoallylic alcohols can be converted into 5- and 6-membered oxasilolanes respectively upon treatment with diphenylsilane and catalytic TBAT. The mechanism appears to involve formation of a carbanion intermediate, limiting the reaction to substrates capable of carbanion stabilization (e.g., through resonance). Additionally, larger groups flanking the alcohol are beneficial, that we surmise may be due to prevention of competing dimerization/oligomerization. These larger groups also increase the diastereoselectivity of oxasilolane formation (5–6:1 for *i*-Pr and *t*-Bu). Studies involving substrates containing differentially substituted phenyl rings indicate that ortho-substitution is deactivating, presumably due to sterics as these groups come in closer proximity to the reacting double bond. Trisubstituted double bonds can also be engaged in this transformation, leading to complex oxasilolanes in good yield and high diastereoselectivity. The stereochemistry of these products, along with those arising from disubstituted double bond-containing compounds was determined using a combination of detailed NMR analysis and further reactions of the oxasilolane products (i.e., Tamao oxidation, and protodesilylation) to generate known compounds. Compared to traditional methods for oxasilolane syntheses by hydrosilylation, this TBAT-catalyzed process is attractive by generating useful synthetic intermediates without the need for precious metal catalysts. Future work will include optimizing this and other reactions to produce highly substituted oxasilolanes.

## Experimental Section

### General Information

All reactions were carried out in vessels under an argon atmosphere unless otherwise specified. Reactions that required heating were conducted on a heating stir plate in heating blocks. Dry solvents were prepared by passing the solvent through a column of activated alumina under nitrogen immediately prior to use. All reagents were purchased and used as received unless mentioned otherwise. TLC analysis used 0.25 mm silica layer fluorescence UV_254_ plates. Column chromatography: silica gel (230–400 mesh). IR: FT-IR with single-bounce diamond ATR. NMR: Spectra were recorded on a 500 MHz spectrometer in CDCl_3_ or C_6_D_6_; chemical shifts (d) are given in ppm, coupling constants (*J*) in Hz. Solvent signals were used as references (CDCl_3_: δ 77.0 ppm; residual CHCl_3_ in CDCl_3_: δ 7.26 ppm, C_6_D_6_: δ 128.06 ppm; residual C_6_H_6_ in C_6_D_6_ δ 7.16 ppm). HRMS: quadrupole time-of-flight LC–MS with electrospray ionization (ESI positive and negative). X-ray crystallography: Samples were prepared by slow evaporation (pentane) at room temperature. A colorless needle, measuring 0.375 × 0.100 × 0.080 mm^3^ was mounted on a loop with oil. Data was collected at −173 °C on a single crystal X-ray diffractometer (Bruker APEXII, Mo-radiation) equipped with an X-ray optical collimator.

### General Procedure A: Room Temperature TBAT-Catalyzed Hydrosilylation

To a solution of the styryl alcohol in toluene (to make a 0.4 M solution) was added diphenylsilane (0.9 equiv) followed by TBAT (0.2 equiv) and the mixture was stirred for 2 h. The reaction was quenched with aq. NH_4_Cl and extracted with MTBE (2 × 15 mL). The combined organic extracts were dried over MgSO_4_, filtered, and concentrated on a rotary evaporator. The crude product was purified by column chromatography on silica (30:1 Hexanes/MTBE).

### General Procedure B: 50 °C TBAT-Catalyzed Hydrosilylation

To a solution of the styryl alcohol in toluene (to make a 0.4 M solution) diphenylsilane (0.9 equiv) followed by TBAT (0.2 equiv) was added, and the mixture was stirred for 2 h at 50 °C. The reaction was quenched with aq. NH_4_Cl and extracted with MTBE (2 × 15 mL). The combined organic extracts were dried over MgSO_4_, filtered, and concentrated on a rotary evaporator. The crude product was purified by column chromatography on silica (30:1 Hexanes/MTBE).

### General Procedure C: 80 °C TBAT-Catalyzed Hydrosilylation

To a solution of styryl alcohol in dry toluene (to make a 0.4 M solution) was added diphenylsilane (0.9 equiv) followed by TBAT (0.2 equiv), and the mixture was stirred for 2 h at 80 °C. The reaction was quenched with aq. NH_4_Cl and extracted with MTBE (2 × 15 mL). The combined organic extracts were dried over MgSO_4_, filtered, and concentrated on a rotary evaporator. The crude product was purified by column chromatography on silica (30:1 Hexanes/MTBE).

### 5-methyl-2,2,3-triphenyl-1,2-oxasilolane (2)

Prepared from (*E*)-4-phenylbut-3-en-2-ol (**1**, 100 mg) according to general procedure B. The oxasilolane product **2** (121 mg, 55%) was obtained as a colorless oil. *Spectral data matched that previously reported*:^16^ ^1^H NMR (500 MHz, CDCl_3_) δ: 7.77 – 7.72 (m, 3H), 7.54 – 7.20 (m, 10H), 7.01 – 6.98 (m, 2H), 4.46 (dtd, *J* = 11.8, 5.9, 3.7 Hz, 1H), 3.28 (dd, *J* = 14.0, 6.8 Hz, 1H), 2.56 – 2.50 (m, 1H), 2.08 (ddd, *J* = 13.9, 12.7, 10.9 Hz, 1H), 1.60 (d, *J* = 6.0 Hz, 3H). IR: 3069, 3024, 2966, 2925, 2862, 1591, 1494, 1451, 1428, 1377, 1116, 1095, 1070, 1028, 1015, 997, 939, 906, 880, 840, 784, 761, 736, 715, 695, 618 cm^–1^. HRMS (ESI+) calcd for C_22_H_23_OSi, (M + H) 331.1513; found, 331.1504.

### 5-butyl-2,2,3-triphenyl-1,2-oxasilolane (**7**)

Prepared from (*E*)-1-phenylhept-1-en-3-ol (**3**, 50 mg) according to general procedure B. The oxasilolane product **7** (79 mg, 81%) was obtained as a colorless oil. Spectral data for the major diastereomer: ^1^H NMR (500 MHz, CDCl_3_) δ: 7.72 – 7.68 (m, 2H), 7.52 – 7.34 (m, 7H), 7.16 – 6.94 (m, 6H), 4.32 – 4.20 (m, 1H), 3.26 – 3.17 (m, 1H), 2.53 – 2.47 (m, 1H), 2.09 – 1.98 (m, 1H), 1.77 (ddt, *J* = 13.4, 11.0, 5.1 Hz, 1H), 1.49 – 1.41 (m, 5H), 0.97 (dt, *J* = 13.5, 7.2 Hz, 3H). ^13^C­{^1^H} NMR (126 MHz, CDCl_3_) δ: 140.8, 135.0, 134.8, 130.8, 130.3, 129.9, 128.2, 128.04, 128.03, 127.4, 127.1, 124.6, 78.4, 38.9, 37.7, 34.8, 28.2, 22.9, 14.1. IR (ATR): 2954, 2929, 2858, 1494, 1452, 1428, 1117, 1052, 1028, 996, 964, 917, 885, 842, 815, 760, 738, 715, 694 cm^–1^. HRMS (ESI+) calcd for C_25_H_29_OSi, (M + H) 373.1982; found, 373.1969.

### 5-isopropyl-2,2,3-triphenyl-1,2-oxasilolane (**8**)

Prepared from (*E*)-4-methyl-1-phenylpent-1-en-3-ol (**4**, 100 mg) according to general procedure A. The oxasilolane product **8** (171 mg, 83%) was obtained as a colorless oil. Spectral data for the major diastereomer: δ ^1^H NMR (500 MHz, CDCl_3_) δ: 7.74 – 7.69 (m, 2H), 7.53 – 7.36 (m, 9H), 7.18 – 7.14 (m, 2H), 7.02 – 6.97 (m, 2H), 3.98 (ddd, *J* = 10.8, 6.9, 3.7 Hz, 1H), 3.20 (dt, *J* = 14.7, 7.3 Hz, 1H), 2.53 – 2.47 (m, 1H), 2.13 – 2.04 (m, 1H), 2.00 (dt, *J* = 13.3, 6.7 Hz, 1H), 1.19 (d, *J* = 6.7 Hz, 3H), 1.07 (d, *J* = 6.8 Hz, 3H). ^13^C­{^1^H} NMR (126 MHz, CDCl_3_) δ: 141.0, 135.8, 135.0, 134.8, 129.8, 128.2, 128.0, 127.7, 127.4, 127.1, 124.6, 83.5, 36.2, 35.0, 34.9, 27.0, 19.4, 18.4. IR (ATR): 2957, 2871, 1590, 1494, 1451, 1428, 1384, 1116, 1066, 1027, 997, 904, 893, 828, 801, 761, 736, 715, 694, 619, 611 cm^–1^. HRMS (ESI+) calcd for C_24_H_27_OSi, (M + H) 359.1826; found, 359.1816.

### 5-(*tert*-butyl)-2,2,3-triphenyl-1,2-oxasilolane (**9**)

Prepared from (*E*)- 4,4-dimethyl-1-phenylpent-1-en-3-ol (**5**, 100 mg) according to general procedure A. The oxasilolane product **9** (148 mg, 75%) was obtained as a colorless oil. S. data for the major diastereomer: δ ^1^H NMR (500 MHz, CDCl_3_) δ: 7.76 – 7.71 (m, 4H), 7.58 – 7.34 (m, 9H), 7.09 – 7.04 (m, 2H), 3.99 (dd, *J* = 11.4, 3.5 Hz, 1H), 3.23 (dd, *J* = 13.5, 6.9 Hz, 1H), 2.44 (ddt, *J* = 9.0, 6.9, 3.5 Hz, 1H), 2.20 (ddd, *J* = 13.6, 12.5, 11.4 Hz, 1H), 1.15 (s, 9H). ^13^C­{^1^H} NMR (126 MHz, CDCl_3_) δ: 141.3, 135.0, 134.7, 130.8, 130.2, 129.8, 128.2, 128.1, 128.0, 127.4, 127.2, 124.6, 86.0, 35.4, 34.8, 26.1. IR (ATR): 3069, 3050, 3025, 2952, 2868, 1601, 1590, 1493, 1477, 1465, 1457, 1452, 1429, 1394, 1363, 1264, 1228, 1188, 1157, 1117, 1056, 1028, 1004, 997, 987, 887, 823, 802, 762, 738, 714, 696 cm^–1^. HRMS (ESI+) calcd for C_25_H_29_OSi, (M + H) 373.1982; found, 373.1969.

### 4,5-dimethyl-2,2,3-triphenyl,1–2,oxasiolane (**10**)

Prepared from (*E*)- 3-methyl-4-phenylbut-3-en-2-ol (50 mg) according to general procedure C. The oxasilolane product **10** (96 mg, 90%) was obtained as a colorless oil. ^1^H NMR (500 MHz, CDCl_3_) δ: 7.70 – 7.66 (m, 2H), 7.49 – 7.36 (m, 7H), 7.14 – 7.03 (m, 4H), 6.87 (dd, *J* = 7.1, 1.6 Hz, 2H), 3.98 (dq, *J* = 9.6, 6.0 Hz, 1H), 2.75 (d, *J* = 12.7 Hz, 1H), 2.18 (ddt, *J* = 16.0, 12.8, 6.4 Hz, 1H), 1.57 (d, *J* = 5.9 Hz, 3H), 1.02 (d, *J* = 6.3 Hz, 3H). ^13^C­{^1^H} NMR (126 MHz, CDCl_3_) δ: 139.8, 135.8, 135.0, 134.7, 128.2, 128.1, 128.1, 128.0, 127.7, 127.5, 124.7, 80.4, 46.7, 43.4, 21.5, 16.2. IR (ATR): 3745, 3069, 2968, 1448, 1437, 1429, 1374, 1363, 1188, 1157, 1116, 1072, 1050, 1027, 997, 972, 934, 913, 858, 839, 802, 770, 788, 738, 716, 696 cm^–1^. HRMS (ESI+) calcd for C_23_H_25_OSi, (M + H) 345.1669; found, 345.1658.

### 3,5-dimethyl-2,2,3-triphenyl,1–2,oxasiolane (**11**)

Prepared from (*E*)- 4-phenylpent-2-en-2-ol (280 mg) according to general procedure C. The oxasilolane product **11** (274 mg, 47%) was obtained as a colorless oil. ^1^H NMR (500 MHz, CDCl_3_) δ: 7.77 – 7.73 (m, 1H), 7.48 – 7.40 (m, 4H), 7.25 – 7.21 (m, 3H), 7.18 – 7.09 (m, 7H), 4.62 (dt, *J* = 10.0, 5.0 Hz, 1H), 2.33 (t, *J* = 11.8 Hz, 1H), 2.09 (dd, *J* = 12.9, 4.0 Hz, 1H), 1.54 – 1.49 (m, 4H), 1.42 (d, *J* = 1.5 Hz, 3H). ^13^C­{^1^H} NMR (126 MHz, CDCl_3_) δ: 146.9, 135.1, 134.8, 130.2, 129.6, 128.4, 127.9, 127.4, 126.6, 125.0, 72.6, 46.6, 35.6, 25.7, 23.7. IR (ATR): 2964, 2923, 2856, 1494, 1457, 1452, 1448, 1444, 1437, 1429, 1375, 1116, 1095, 1069, 1042, 1028, 997, 946, 908, 865, 811, 784, 760, 737, 712, 695 cm^–1^. HRMS (ESI+) calcd for C_23_H_25_OSi, (M + H) 345.1669 Found 345.1662.

### (*E*)-1-(furan-2-yl)­hept-1-en-3-ol (**19**)

To a solution of (*E*)-3-(furan-2-yl)­acrylaldehyde (500 mg, 4.0 mmol) in THF (8.2 mL), 1.6 M *n*-BuLi (3.1 mL, 5.0 mmol) was added, and the mixture was stirred for 1 h at −78 °C. The reaction was quenched with aq. NH_4_Cl (15 mL) and extracted with MTBE (2 × 15 mL). The combined organic extracts were dried over MgSO_4_, filtered, and concentrated on a rotary evaporator. The product **19** (698 mg, 94%) was obtained as a colorless oil and used without further purification. ^1^H NMR (500 MHz, CDCl_3_) δ: 7.37 (d, *J* = 1.8 Hz, 1H), 6.43 (dd, *J* = 15.8, 1.2 Hz, 1H), 6.39 (dd, *J* = 3.2, 1.8 Hz, 1H), 6.25 (d, *J* = 3.2 Hz, 1H), 6.20 (dd, *J* = 15.8, 6.5 Hz, 1H), 4.26 (qd, *J* = 6.4, 1.3 Hz, 1H), 1.69 – 1.59 (m, 3H), 1.46 – 1.33 (m, 4H), 0.98 – 0.89 (m, 3H). ^13^C­{^1^H} NMR (126 MHz, CDCl_3_) δ: 152.5, 141.9, 131.3, 118.5, 111.3, 107.9, 72.6, 37.1, 27.6, 22.7, 14.0. IR (ATR): 3368, 2957, 2931, 2860, 1490, 1466, 1457, 1378, 1255, 1200, 1152, 1131, 1072, 1012, 960, 927, 884, 847, 795, 729 cm^–1^. HRMS (ESI+) calcd for C_11_H_17_O_2_, (M + H) 181.1223; found, 181.1221.

### (6*E*,8*E*)-deca-6,8-dien-5-ol (**20**)

To a solution of (2*E*,4*E*)-hexa-2,4-dienal (0.50 g, 5.0 mmol) in THF (10 mL), *n*-BuLi (1.6 M, 3.1 mL, 5.0 mmol) was added, and the mixture was stirred for 1 h at −78 °C. The reaction was quenched with aq. NH_4_Cl (15 mL) and extracted with MTBE (2 × 15 mL). The combined organic extracts were dried over MgSO_4_, filtered, and concentrated on a rotary evaporator. The crude product **20** was obtained as a colorless oil (0.58 g, 76%). ^1^H NMR (500 MHz, CDCl_3_) δ: 6.17 (dd, *J* = 15.2, 10.4 Hz, 1H), 6.08 – 5.99 (m, 1H), 5.75 – 5.67 (m, 1H), 5.56 (dd, *J* = 15.1, 7.0 Hz, 1H), 4.10 (qd, *J* = 6.6, 3.7 Hz, 1H), 1.78 – 1.74 (m, 3H), 1.62 – 1.47 (m, 2H), 1.38 – 1.25 (m, 4H), 0.93 – 0.88 (m, 3H). ^13^C­{^1^H} NMR (126 MHz, CDCl_3_) δ: 133.5, 130.8, 130.8, 129.9, 73.0, 37.1, 27.6, 22.7, 18.1, 14.0. IR (ATR): 2957, 2930, 2873, 2859, 1457, 1378, 1363, 1340, 1130, 1045, 1022, 984, 947, 927, 899, 719, 668 cm^–1^. HRMS (ESI+) calcd for C_10_H_19_O, (M + H) 155.1430; found, 153.1434.

### 5-butyl-3-(furan-2-yl)-2,2-diphenyl-1,2-oxasilolane (**21**)

Prepared from (*E*)-1-(furan-2-yl)­hept-1-en-3-ol (50 mg) according to general procedure C. The oxasilolane product **21** (84 mg, 77%) was obtained as a colorless oil. S. data for the major diastereomer: ^1^H NMR (500 MHz, CDCl_3_) δ: 7.80 – 7.77 (m, 1H), 7.51 – 7.42 (m, 5H), 7.40 – 7.33 (m, 5H), 6.19 (dt, *J* = 3.3, 1.9 Hz, 1H), 5.72 (dt, *J* = 3.2, 1.0 Hz, 1H), 4.27 – 4.16 (m, 1H), 3.20 – 3.10 (m, 1H), 2.56 (ddd, *J* = 12.7, 7.1, 3.8 Hz, 1H), 1.94 (td, *J* = 13.2, 11.1 Hz, 1H), 1.78 – 1.70 (m, 2H), 1.50 – 1.38 (m, 4H), 0.97 (t, *J* = 7.1 Hz, 3H). S. data for the major and minor diastereomer: ^13^C­{^1^H} NMR (126 MHz, chloroform-d) δ: 156.3, 155.6, 140.2, 140.2, 135.2, 134.8, 134.7, 134.6, 134.6, 134.5, 134.2, 132.9, 132.7, 130.4, 130.3, 130.1, 130.0, 128.1, 128.0, 127.6, 110.2, 110.2, 103.0, 103.0, 78.3, 78.2, 37.6, 37.5, 37.2, 36.4, 28.4, 28.2, 27.8, 25.6, 22.9, 22.7, 14.1, 14.1. IR (ATR): 2954, 2929, 2858, 1653, 1590, 1577, 1507, 1465, 1457, 1448, 1437, 1429, 1420, 1171, 1118, 1075, 1054, 1010, 997, 965, 942, 918, 883, 819, 791, 768, 737, 715, 696 cm^–1^. HRMS (ESI+) calcd for C_23_H_27_O_2_Si, (M + H) 363.1775; found, 363.1770.

### (*E*)-5-butyl-2,2-diphenyl-3-(prop-1-en-1-yl)-1,2-oxasilolane (**22**)

Prepared from (6*E*,8*E*)-deca-6,8-dien-5-ol (50 mg) according to general procedure C. The oxasilolane product **22** (98 mg, 91%) was obtained as a colorless oil. S. data for the major and minor diastereomer: ^1^H NMR (500 MHz, CDCl_3_) δ: 7.71 (ddt, *J* = 8.0, 6.4, 1.5 Hz, 4H), 7.67 – 7.62 (m, 4H), 7.50 – 7.40 (m, 12H), 5.46 – 5.42 (m, 2H), 5.40 – 5.34 (m, 2H), 4.50 – 4.43 (m, 1H), 4.15 (dddd, *J* = 10.8, 6.8, 5.8, 3.8 Hz, 1H), 2.60 (dtd, *J* = 17.7, 7.9, 6.5 Hz, 2H), 2.31 (ddd, *J* = 12.9, 7.3, 3.9 Hz, 1H), 2.19 (ddd, *J* = 13.4, 8.2, 6.0 Hz, 1H), 2.03 (ddd, *J* = 13.3, 7.5, 5.0 Hz, 1H), 1.92 – 1.84 (m, 1H), 1.78 – 1.71 (m, 2H), 1.64 (dt, *J* = 5.0, 1.4 Hz, 6H), 1.60 – 1.53 (m, 2H), 1.51 – 1.39 (m, 8H), 0.99 (dtd, *J* = 13.2, 7.2, 4.4 Hz, 6H). ^13^C­{^1^H} NMR (126 MHz, CDCl_3_) δ: 135.2, 135.1, 135.1, 134.6, 134.4, 130.9, 130.6, 130.2, 130.1, 130.1, 129.9, 129.9, 129.8, 128.0, 127.9, 127.7, 127.6, 122.4, 122.2, 121.0, 78.5, 77.9, 39.6, 37.8, 37.7, 37.5, 31.8, 28.8, 28.4, 28.2, 28.2, 22.9, 22.7, 17.9, 14.1, 14.1. IR (ATR): 2955, 2928, 2856, 1465, 1457, 1437, 1428, 1420, 1117, 1052, 1029, 1010, 993, 916, 879, 870, 845, 801, 767, 729, 708, 696 cm^–1^. HRMS (ESI+) calcd for C_22_H_29_OSi, (M + H) 337.1982; found, 337.1975.

### 6-butyl-2,2,3-triphenyl-1,2-oxasilinane (**23**)

Prepared from (*E*)-1-phenyloct-1-en-4-ol^53^ (50 mg) according to general procedure A. The oxasilolane product **23** (68 mg, 71%) was obtained as a colorless oil. S. data for the major diastereomer: ^1^H NMR (500 MHz, CDCl_3_) δ: 7.72 – 7.65 (m, 1H), 7.62 – 7.55 (m, 2H), 7.47 – 7.34 (m, 8H), 7.28 – 7.22 (m, 2H), 7.20 – 7.15 (m, 2H), 4.15 (dddd, *J* = 11.1, 6.9, 4.3, 2.2 Hz, 1H), 2.83 (dd, *J* = 13.5, 4.1 Hz, 1H), 2.27 (dtd, *J* = 13.1, 4.4, 2.5 Hz, 1H), 2.15 (qd, *J* = 13.4, 2.5 Hz, 1H), 2.00 (ddt, *J* = 13.9, 4.7, 2.3 Hz, 1H), 1.76 – 1.67 (m, 3H), 1.66 – 1.57 (m, 2H), 1.41 (dddd, *J* = 14.7, 12.9, 7.8, 1.9 Hz, 2H), 0.97 (dd, *J* = 7.8, 6.9 Hz, 3H). ^13^C­{^1^H} NMR (126 MHz, CDCl_3_) δ: 142.4, 135.8, 135.3, 134.9, 128.1, 127.8, 127.4, 127.3, 124.7, 75.9, 38.5, 36.0, 34.4, 28.1, 27.8, 22.8, 14.2. IR (ATR): 2930, 2855, 1598, 1590, 1428, 1116, 1092, 1081, 1061, 1027, 995, 966, 908, 801, 758, 740, 735, 713, 695 cm^–1^. HRMS (ESI+) calcd for C_26_H_31_OSi, (M + H) 387.2139; found, 387.2138.

### 6-isopropyl-2,2,3-triphenyl-1,2-oxasilinane (**24**)

Prepared from (*E*)-2-methyl-6-phenylhex-5-en-3-ol^54^ (50 mg) according to general procedure A. The oxasilolane product **24** (72 mg, 74%) was obtained as a colorless oil. S. data for the major diastereomer: ^1^H NMR (500 MHz, CDCl_3_) δ: 7.64 – 7.55 (m, 2H), 7.40 – 7.36 (m, 3H), 7.26 – 7.21 (m, 2H), 7.22 – 7.12 (m, 4H), 7.12 – 7.09 (m, 2H), 6.90 (ddd, *J* = 8.1, 1.7, 1.0 Hz, 2H), 3.95 (ddd, *J* = 11.2, 5.1, 2.2 Hz, 1H), 2.79 (dd, *J* = 13.5, 4.1 Hz, 1H), 2.29 (dtd, *J* = 13.3, 4.8, 2.6 Hz, 1H), 2.13 (qd, *J* = 13.2, 2.4 Hz, 1H), 1.98 (ddt, *J* = 13.7, 4.5, 2.2 Hz, 1H), 1.88 (pd, *J* = 6.8, 4.9 Hz, 1H), 1.72 (dddd, *J* = 15.3, 13.6, 11.4, 2.5 Hz, 1H), 1.09 (d, *J* = 6.8 Hz, 3H), 1.07 (d, *J* = 6.8 Hz, 3H). ^13^C­{^1^H} NMR (126 MHz, CDCl_3_) δ: 142.4, 135.3, 134.8, 132.6, 129.9, 129.6, 129.2, 128.1, 127.8, 127.5, 127.3, 124.7, 80.5, 34.8, 34.7, 32.4, 28.0, 18.7, 17.8. IR (ATR): 2961, 2929, 2851, 1598, 1590, 1428, 1365, 1157, 1121, 963, 950, 908, 840, 801, 758, 738, 714, 696 cm^–1^. HRMS (ESI+) calcd for C_25_H_29_OSi, (M + H) 373.1982; found, 373.1982.

### 6-(*tert*-butyl)-2,2,3-triphenyl-1,2-oxasilinane (**25**)

Prepared from (*E*)-2,2-dimethyl-6-phenylhex-5-en-3-ol^55^ (50 mg) according to general procedure A. The oxasilolane product **25** (89 mg, 94%) was obtained as a white solid. S. data for the major diastereomer: ^1^H NMR (500 MHz, CDCl_3_) δ: 7.66 – 7.57 (m, 2H), 7.48 – 7.44 (m, 1H), 7.41 – 7.36 (m, 4H), 7.27 – 7.23 (m, 2H), 7.22 – 7.18 (m, 2H), 7.12 (dd, *J* = 8.0, 1.5 Hz, 2H), 6.95 – 6.89 (m, 2H), 3.83 (dd, *J* = 11.3, 2.0 Hz, 1H), 2.77 (dd, *J* = 13.6, 4.0 Hz, 1H), 2.32 (dddd, *J* = 13.1, 4.8, 3.9, 2.3 Hz, 1H), 2.12 (qd, *J* = 13.2, 2.3 Hz, 1H), 2.05 (ddt, *J* = 13.7, 4.7, 2.2 Hz, 1H), 1.70 (dddd, *J* = 13.7, 12.7, 11.2, 2.4 Hz, 1H), 1.08 (s, 9H). ^13^C­{^1^H} NMR (126 MHz, CDCl_3_) δ: 142.5, 135.3, 134.8, 132.7, 129.9, 129.6, 129.5, 128.7, 128.1, 127.7, 127.5, 127.3, 124.7, 83.4, 35.6, 34.8, 29.8, 28.1, 26.0. IR (ATR): 2956, 2840, 2118, 1597, 1427, 1359, 1333, 1108, 1091, 1081, 1062, 1028, 1003, 995, 953, 945, 908, 900, 870, 798, 786, 771, 755, 741, 735, 713, 697 cm^–1^. HRMS (ESI+) calcd for C_26_H_31_OSi, (M + H) 387.2139; found, 387.2138.

### 2,2,3,6-tetraphenyl-1,2-oxasilinane (**26**)

Prepared from (*E*)-1,4-diphenylbut-3-en-1-ol^56^ (50 mg) according to general procedure A. The oxasilolane product **26** (34 mg, 37%) was obtained as a white solid. S. data for the major diastereomer: ^1^H NMR (500 MHz, CDCl_3_) δ: 7.85 – 7.79 (m, 1H), 7.74 – 7.68 (m, 2H), 7.63 – 7.55 (m, 2H), 7.52 – 7.39 (m, 6H), 7.31 – 7.26 (m, 3H), 7.26 – 7.17 (m, 4H), 7.02 – 6.92 (m, 2H), 5.32 (dd, *J* = 11.2, 2.3 Hz, 1H), 3.01 – 2.93 (m, 1H), 2.51 – 2.36 (m, 2H), 2.37 – 2.27 (m, 1H), 2.10 – 1.91 (m, 1H). ^13^C­{^1^H} NMR (126 MHz, CDCl_3_) δ: 144.9, 142.0, 135.4, 134.9, 131.8, 130.2, 129.9, 129.3, 128.3, 128.2, 127.9, 127.6, 127.3, 127.2, 125.3, 124.9, 77.5, 39.3, 34.2, 28.3. IR (ATR): 3067, 2913, 2840, 1589, 1492, 1448, 1427, 1345, 1115, 1109, 1090, 1084, 1058, 1029, 1009, 997, 982, 929, 906, 883, 833, 790, 766, 750, 744, 738, 732, 714, 694 cm^–1^. HRMS (ESI+) calcd for C_28_H_27_OSi, (M + H) 407.1826; found, 407.1824.

### (*Z*)-1-phenyloct-1-en-4-ol (**27**)

To a solution of 1-phenyloct-1-yn-4-ol (**27**, 200 mg) in toluene (10 mL, 0.1 M) 1-hexene (0.03 mL, 5.0 equiv) and Lindlar’s catalyst (106 mg, 1.0 equiv) were added. The headspace was filled with hydrogen gas and stirred for 2 h. After purging the headspace with argon, the solution was filtered through Celite and concentrated under reduced pressure. The *cis-*alkene **28** (192 mg, 96%) was obtained as a colorless oil. ^1^H NMR: (500 MHz, CDCl_3_) δ: 7.39 – 7.30 (m, 3H), 7.26 (ddt, *J* = 7.6, 6.3, 1.8 Hz, 1H), 7.23 – 7.19 (m, 1H), 6.61 (dd, *J* = 11.7, 1.9 Hz, 1H), 5.78 (dt, *J* = 11.7, 7.4 Hz, 1H), 3.76 (qd, *J* = 6.2, 3.1 Hz, 1H), 2.53 (ddd, *J* = 7.5, 6.1, 1.8 Hz, 2H), 1.56 – 1.47 (m, 2H), 1.39 – 1.28 (m, 4H), 0.93 (dtd, *J* = 7.2, 5.0, 2.6 Hz, 3H). ^13^C­{^1^H} NMR (126 MHz, CDCl_3_) δ: 137.3, 131.5, 128.8, 128.4, 128.2, 126.8, 71.9, 36.8, 36.5, 27.9, 22.7, 14.1. IR (ATR): 3354, 2955, 2927, 2858, 1600, 1466, 1457, 1448, 1378, 1126, 1081, 964, 913, 862, 804, 768, 697, 668 cm^–1^. HRMS (ESI+) calcd for C_14_H_21_O, (M + H) 205.1592; found, 205.1592.

### (*E*)-1-phenylnon-1-en-5-ol (**29**)

To a solution of (4*E*)-5-phenyl-4-pentenal (100 mg) in THF (2 mL) at 78 °C was added *n*-BuLi (0.5 mL, 2.0 equiv) and the mixture was stirred for 2 h. The reaction was quenched with aq. NH_4_Cl (15 mL) and extracted with EtOAc (2 × 15 mL). The combined organic extracts were dried over MgSO_4_, filtered, and concentrated on a rotary evaporator. The crude product was purified by column chromatography on silica (4:1 Hexanes/EtOAc) to give the secondary alcohol **29** (46 mg, 33%) as a colorless oil. ^1^H NMR (500 MHz, CDCl_3_) δ: 7.39 – 7.35 (m, 2H), 7.34 – 7.30 (m, 2H), 7.23 (qt, *J* = 6.6, 1.4 Hz, 1H), 6.45 (dt, *J* = 15.8, 1.5 Hz, 1H), 6.27 (dt, *J* = 15.8, 6.9 Hz, 1H), 3.74 – 3.62 (m, 1H), 2.41 (dddt, *J* = 13.6, 7.6, 5.9, 1.6 Hz, 1H), 2.37 – 2.28 (m, 1H), 1.75 – 1.58 (m, 2H), 1.56 – 1.42 (m, 3H), 1.40 – 1.30 (m, 3H), 0.94 (t, *J* = 7.1 Hz, 3H). ^13^C­{^1^H} NMR (126 MHz, CDCl_3_) δ: 137.7, 130.5, 130.2, 128.5, 126.9, 126.0, 71.5, 37.3, 36.9, 29.3, 27.8, 22.8, 14.1. IR (ATR): 3349, 2956, 2928, 2858, 1429, 1126, 1072, 964, 740, 696 cm^–1^. HRMS (ESI+) calcd for C_15_H_23_O, (M + H) 218.1671; found, 218.1672.

### (*E*)-2-methyl-1-phenyloct-1-en-4-ol (**30**)

To a solution of (*E*)-3-methyl-4-phenylbut-3-enal (364 mg) in THF (8 mL, 0.3 M) at −78 °C was added *n*-BuLi (1.8 mL, 2.0 equiv) and the mixture was stirred for 2 h. The reaction was quenched with aq. NH_4_Cl (15 mL) and extracted with EtOAc (2 × 15 mL). The combined organic extracts were dried over MgSO_4_, filtered, and concentrated on a rotary evaporator. The crude product was purified by column chromatography on silica (4:1 Hexanes/EtOAc) to give the tertiary alkene **30** (258 mg, 52%) as a colorless oil. ^1^H NMR (500 MHz, CDCl_3_) δ: 7.38 – 7.33 (m, 2H), 7.30 – 7.27 (m, 2H), 7.25 – 7.21 (m, 1H), 6.39 (s, 1H), 3.84 (ddt, *J* = 12.1, 6.7, 3.5 Hz, 1H), 2.40 (ddd, *J* = 13.3, 3.7, 1.3 Hz, 1H), 2.26 (ddd, *J* = 13.4, 9.2, 0.9 Hz, 1H), 1.94 (d, *J* = 1.4 Hz, 3H), 1.58 – 1.52 (m, 2H), 1.44 – 1.35 (m, 4H), 0.96 (t, *J* = 7.2 Hz, 3H). ^13^C­{^1^H} NMR (126 MHz, CDCl_3_) δ: 137.9, 135.8, 128.9, 128.2, 128.1, 126.3, 69.1, 49.1, 36.9, 28.0, 22.8, 18.0, 14.1. IR (ATR): 3379, 2955, 2929, 2858, 1652, 1599, 1490, 1466, 1441, 1378, 1123, 1074, 1027, 1010, 916, 828, 741, 697 cm^–1^. HRMS (ESI+) calcd for C_15_H_23_O, (M + H) 219.1743; found, 219.1739.

### (*E*)-2-phenylnon-2-en-5-ol (**31**)

A solution *t*-BuLi (3.2 mL, 2.2 equiv) in Et_2_O (0.05 M) was cooled to −78 °C. (*E*)-(1-iodoprop-1-en-2-yl)­benzene (562 mg) was added and stirred for 20 min 1,2-Epoxyhexane (0.42 mL, 1.5 equiv) was added then the reaction was transferred to a −10 °C brine bath and stirred for 1 h. The reaction was quenched with aq. NH_4_Cl (15 mL) and extracted with EtOAc (2 × 15 mL). The combined organic extracts were dried over MgSO_4_, filtered, and concentrated on a rotary evaporator. The crude product was purified by column chromatography on silica (4:1 Hexanes/EtOAc) to give the tertiary alkene **30** (205 mg, 41%) as a colorless oil. ^1^H NMR (500 MHz, CDCl_3_) δ: 7.45 – 7.41 (m, 2H), 7.38 – 7.31 (m, 2H), 7.30 – 7.24 (m, 1H), 5.86 (tt, *J* = 7.2, 1.4 Hz, 1H), 3.77 (tt, *J* = 7.3, 5.1 Hz, 1H), 2.50 – 2.39 (m, 2H), 2.10 (t, *J* = 1.1 Hz, 3H), 1.57 (dddd, *J* = 14.8, 9.2, 7.9, 4.6 Hz, 2H), 1.43 – 1.33 (m, 4H), 0.95 (t, *J* = 7.1 Hz, 3H). ^13^C­{^1^H} NMR (126 MHz, CDCl^3^) δ: 143.6, 137.7 (d, *J* = 1.4 Hz), 128.2, 126.8, 125.7, 124.0, 71.8, 37.0, 36.7, 28.0, 22.8, 16.2, 14.1. IR (ATR): 3375, 2955, 2928, 2858, 1598, 1493, 1466, 1444, 1378, 1127, 1026, 906, 878, 756, 694 cm^–1^. HRMS (ESI+) calcd for C_15_H_23_O, (M + H) 219.1743; found, 219.1744.

### 6-butyl-4-methyl-2,2,3-triphenyl-1,2-oxasilinane (**32**)

Prepared from (*E*)-2-methyl-6-phenylhex-5-en-3-ol (**29**, 50 mg) according to general procedure C. The oxasilolane product **30** (38 mg, 41%) was obtained as a colorless oil. ^1^H NMR (500 MHz, CDCl3) δ: 7.74 (dddd, *J* = 5.8, 5.1, 2.9, 1.4 Hz, 2H), 7.63 – 7.52 (m, 2H), 7.40 – 7.26 (m, 2H), 7.20 – 7.11 (m, 4H), 7.04 (dd, *J* = 8.1, 6.5 Hz, 2H), 7.01 – 6.95 (m, 1H), 6.88 – 6.82 (m, 2H), 4.20 (dddd, *J* = 11.2, 7.7, 4.0, 2.0 Hz, 1H), 2.44 (d, *J* = 12.3 Hz, 1H), 2.19 (tqd, *J* = 12.6, 6.3, 2.6 Hz, 1H), 1.69 (dt, *J* = 8.9, 1.6 Hz, 1H), 1.59 (dt, *J* = 13.9, 2.4 Hz, 1H), 1.56 – 1.47 (m, 2H), 1.42 – 1.32 (m, 4H), 0.94 (t, *J* = 7.3 Hz, 3H), 0.88 (d, *J* = 6.4 Hz, 3H). ^13^C­{^1^H} NMR (126 MHz, CDCl_3_) δ: 140.3, 135.8, 135.5, 134.8, 129.8, 129.5, 128.8, 128.1, 127.9, 127.7, 127.3, 124.6, 75.1, 44.4, 42.8, 38.7, 33.3, 27.8, 22.9, 22.2, 14.2. IR (ATR): 2924, 2857, 1466, 1456, 1429, 1377, 1150, 1113, 1068, 1039, 1026, 998, 938, 915, 801, 765, 743, 738, 728, 720, 696 cm^–1^. HRMS (ESI+) calcd for C_15_H_23_O, (M + H) 219.1743; found, 219.1744.

### 6-butyl-3-methyl-2,2,3-triphenyl-1,2-oxasilinane (**33**)

Prepared from (*E*)-2-phenylnon-2-en-5-ol (**27**, 50 mg) according to general procedure C. The oxasilolane product **28** (74 mg, 81%) was obtained as a colorless oil. ^1^H NMR (500 MHz, CDCl_3_) δ: 7.62 (dq, *J* = 6.5, 1.3 Hz, 2H), 7.47 – 7.43 (m, 1H), 7.43 – 7.36 (m, 4H), 7.34 – 7.29 (m, 2H), 7.25 – 7.22 (m, 1H), 7.21 – 7.18 (m, 1H), 7.18 – 7.14 (m, 2H), 7.01 – 6.82 (m, 2H), 4.01 (dddd, *J* = 11.3, 7.2, 4.2, 2.6 Hz, 1H), 2.45 (td, *J* = 13.8, 3.2 Hz, 1H), 2.01 (tdd, *J* = 10.8, 3.8, 2.6 Hz, 2H), 1.77 (dddd, *J* = 15.2, 8.0, 5.6, 4.2 Hz, 1H), 1.68 – 1.60 (m, 2H), 1.55 (s, 3H), 1.52 – 1.37 (m, 4H), 0.98 (t, *J* = 7.3 Hz, 3H). ^13^C­{^1^H} NMR (126 MHz, CDCl_3_) δ: 148.0, 135.8, 135.6, 135.2, 129.9, 129.8, 128.3, 128.1, 127.51, 127.48, 126.7, 125.1, 74.6, 38.3, 34.3, 30.9, 29.3, 27.6, 24.7, 22.9, 14.2. IR (ATR): 2954, 2928, 2858, 1428, 1114, 1084, 1055, 1028, 997, 947, 801, 789, 756, 737, 729, 711, 696 cm^–1^. HRMS (ESI+) calcd for C_27_H_33_OSi, (M + H) 401.2295; found, 401.2296.

### 5-butyl-3-(4-fluorophenyl)-2,2-diphenyl-1,2-oxasilolane

Prepared from (*E*)-1-(4-fluorophenyl)­hept-1-en-3-ol (50 mg) according to general procedure B. The oxasiolane product (56 mg, 60%) was obtained as a colorless oil. S. data for the major diastereomer: ^1^H NMR (500 MHz, CDCl_3_) δ: 7.71 – 7.67 (m, 3H), 7.51 – 7.40 (m, 7H), 6.93 – 6.86 (m, 2H), 6.82 (td, *J* = 8.8, 1.4 Hz, 2H), 4.28 – 4.22 (m, 1H), 3.20 – 3.14 (m, 1H), 2.49 (dtd, *J* = 13.2, 6.6, 3.4 Hz, 1H), 1.96 (ddd, *J* = 13.8, 12.7, 10.9 Hz, 1H), 1.82 – 1.73 (m, 2H), 1.50 – 1.39 (m, 4H), 0.98 (t, *J* = 7.1 Hz, 3H). ^13^C­{^1^H} NMR (126 MHz, CD_2_Cl_2_) δ: 159.6, 133.3, 133.1, 132.9, 130.5, 128.9, 128.6, 128.2, 126.4, 126.3, 126.2, 126.2, 125.7, 113.1, 76.5, 37.4, 35.8, 32.0, 26.3, 21.0, 12.2. IR (ATR): 2954, 2929, 2858, 1603, 1590, 1507, 1486, 1465, 1457, 1428, 1221, 1187, 1158, 1117, 1092, 1053, 996, 964, 917, 888, 853, 829, 794, 767, 738, 720, 710, 696 cm^–1^. HRMS (ESI+) calcd for C_25_H_28_FOSi, (M + H) 391.1888; found, 391.1878.

### 3-(4-chlorophenyl)-5-isopropyl-2,2-diphenyl-1,2-oxasilolane

Prepared from (*E*)-1-(4-chlorophenyl)­hept-1-en-3-o (50 mg) according to general procedure B. The oxasiolane product (53 mg, 56%) was obtained as a colorless oil. S. data for the major diastereomer: ^1^H NMR (500 MHz, CDCl_3_) δ: 7.71 – 7.66 (m, 3H), 7.46 – 7.37 (m, 7H), 7.12 – 7.08 (m, 2H), 6.92 – 6.87 (m, 2H), 3.96 (ddd, *J* = 10.8, 6.9, 3.7 Hz, 1H), 3.19 – 3.11 (m, 1H), 2.47 (ddd, *J* = 12.5, 6.7, 3.7 Hz, 1H), 2.03 – 1.95 (m, 2H), 1.18 (d, *J* = 6.7 Hz, 3H), 1.05 (d, *J* = 6.7 Hz, 3H). ^13^C­{^1^H} NMR (126 MHz, CD_2_Cl_2_) δ: 137.8, 133.3, 133.1, 132.9, 128.5, 128.2, 126.5, 126.3, 126.2, 125.8, 125.7, 81.6, 34.6, 33.1, 32.6, 17.5, 16.5. IR (ATR): 3069, 2958, 2871, 1490, 1428, 1385, 1364, 1116, 1089, 1027, 1012, 996, 895, 870, 824, 738, 707, 696 cm^–1^. HRMS (ESI+) calcd for C_24_H_26_ClOSi, (M + H) 393.1441; found, 392.1443.

### 3-(4-bromophenyl)-5-butyl-2,2-diphenyl-1,2-oxasilolane

Prepared from (*E*)-1-(4-bromophenyl)­hept-1-en-3-ol (50 mg) according to general procedure B. The oxasiolane product (149 mg, 79%) was obtained as a colorless oil. S. data for the major diastereomer: ^1^H NMR (500 MHz, CDCl_3_) δ: 7.69 (ddd, *J* = 8.1, 6.9, 1.5 Hz, 3H), 7.65 – 7.61 (m, 2H), 7.46 – 7.39 (m, 7H), 6.87 – 6.82 (m, 2H), 3.96 (ddd, *J* = 10.8, 6.9, 3.7 Hz, 1H), 3.17 – 3.09 (m, 1H), 2.48 (ddd, *J* = 12.6, 6.7, 3.7 Hz, 1H), 2.06 – 1.96 (m, 2H), 1.19 (d, *J* = 6.7 Hz, 3H), 1.06 (d, *J* = 6.7 Hz, 3H). ^13^C­{^1^H} NMR (126 MHz, CD_2_Cl_2_) δ: 138.4, 133.1, 132.9, 132.5, 129.3, 128.6, 128.2, 126.9, 126.2, 125.9, 125.7, 81.6, 34.6, 33.1, 32.8, 17.6, 16.5. IR (ATR): 2958, 2871, 2123, 1589, 1487, 1428, 1402, 1384, 1330, 1304, 1187, 1114, 1071, 1008, 996, 896, 819, 801, 729, 696, 673 cm^–1^. HRMS (ESI+) calcd for C_24_H_26_BrOSi, (M + H) 437.0930; found, 437.0916.

### 5-butyl-2,2-diphenyl-3-(*p*-tolyl)-1,2-oxasilolane

Prepared from (*E*)-1-(*p*-tolyl)­hept-1-en-3-ol (50 mg) according to general procedure B. The oxasiolane product (84 mg, 90%) was obtained as a colorless oil. S. data for the major diastereomer: ^1^H NMR (500 MHz, CDCl_3_) δ: 7.67 (dq, *J* = 6.7, 1.6 Hz, 3H), 7.48 – 7.34 (m, 7H), 6.92 (dd, *J* = 8.1, 2.4 Hz, 2H), 6.85 – 6.81 (m, 2H), 4.24 – 4.18 (m, 1H), 3.16 – 3.09 (m, 1H), 2.46 (dtd, *J* = 12.9, 6.7, 3.5 Hz, 1H), 2.24 (s, 3H), 1.97 (ddd, *J* = 14.0, 12.7, 10.9 Hz, 1H), 1.74 (dddd, *J* = 21.3, 11.0, 5.3, 2.6 Hz, 2H), 1.46 – 1.37 (m, 4H), 0.97 – 0.91 (m, 3H). ^13^C­{^1^H} NMR (126 MHz, CDCl_3_) δ: 138.5, 137.6, 135.2, 135.2, 135.0, 134.8, 134.6, 134.6, 134.0, 134.0, 132.9, 132.8, 130.3, 130.2, 129.9, 129.8, 128.9, 128.9, 128.1, 128.0, 127.5, 127.5, 127.1, 127.1, 78.4, 78.2, 39.4, 37.9, 37.8, 37.6, 34.4, 31.3, 28.5, 28.3, 22.9, 22.7, 20.9, 20.9, 14.2, 14.1. IR (ATR): 2954, 2927, 2858, 1700, 1653, 1512, 1465, 1457, 1448, 1436, 1428, 1419, 1363, 1117, 1109, 1004, 996, 964, 908, 888, 850, 814, 782, 766, 735, 720, 711, 697 cm^–1^. HRMS (ESI+) calcd for C_26_H_31_OSi, (M + H) 387.2139; found, 387.2131.

### 5-butyl-3-(4-methoxyphenyl)-2,2-diphenyl-1,2-oxasilolane

Prepared from (*E*)-1-(4-methoxyphenyl)­hept-1-en-3-ol (50 mg) according to general procedure B. The oxasiolane product (64 mg, 70%) was obtained as a colorless oil. S. data for the major diastereomer: ^1^H NMR (500 MHz, CDCl_3_) δ: 7.69 (dt, *J* = 8.1, 1.5 Hz, 3H), 7.48 – 7.33 (m, 7H), 6.89 – 6.86 (m, 2H), 6.70 (dd, *J* = 8.8, 2.6 Hz, 2H), 4.28 – 4.21 (m, 1H), 3.76 (s, 3H), 3.17 – 3.10 (m, 1H), 2.52 – 2.45 (m, 1H), 2.01 – 1.92 (m, 1H), 1.84 – 1.72 (m, 2H), 1.52 – 1.39 (m, 4H), 0.98 (t, *J* = 7.1 Hz, 3H). ^13^C­{^1^H} NMR (126 MHz, CDCl_3_) δ: 157.0, 156.9, 135.3, 135.2, 135.0, 135.0, 134.8, 134.6, 133.7, 132.9, 132.8, 132.8, 130.3, 130.2, 129.9, 129.8, 128.1, 128.0, 127.6, 127.6, 127.5, 127.5, 113.7, 113.7, 78.4, 78.2, 55.3, 55.2, 39.5, 38.0, 37.8, 37.6, 33.7, 30.6, 28.5, 28.2, 22.9, 22.7, 14.2, 14.1. IR (ATR): 2954, 2928, 2856, 1609, 1509, 1428, 1419, 1244, 1178, 1117, 1109, 1036, 1003, 996, 964, 916, 888, 825, 778, 738, 726, 712, 697 cm^–1^. HRMS (ESI+) calcd for C_26_H_31_O_2_Si, (M + H) 403.2088; found, 403.2083.

### 5-butyl-2,2-diphenyl-3-(m-tolyl)-1,2-oxasilolane

Prepared from (*E*)-1-(m-tolyl)­hept-1-en-3-ol (50 mg) according to general procedure B. The oxasiolane product (61 mg, 66%) was obtained as a colorless oil. S. data for the major diastereomer: ^1^H NMR (500 MHz, CDCl_3_) δ: 7.72 (ddd, *J* = 7.9, 3.4, 1.5 Hz, 4H), 7.53 – 7.39 (m, 7H), 7.04 (td, *J* = 7.6, 1.8 Hz, 2H), 4.26 (dddd, *J* = 10.8, 6.8, 5.8, 3.8 Hz, 1H), 3.16 (dd, *J* = 8.0, 6.2 Hz, 1H), 2.56 – 2.48 (m, 1H), 2.19 (s, 3H), 2.02 (ddd, *J* = 13.9, 12.7, 10.9 Hz, 1H), 1.86 – 1.74 (m, 2H), 1.50 – 1.41 (m, 4H), 1.00 (t, *J* = 7.2 Hz, 3H). ^13^C­{^1^H} NMR (126 MHz, CDCl_3_) δ: 141.6, 140.8, 137.6, 135.2, 135.0, 134.8, 134.6, 130.2, 129.8, 128.0, 127.4, 125.4, 124.3, 78.3, 39.3, 37.6, 34.7, 28.2, 22.8, 21.4, 14.1. IR (ATR): 2955, 2928, 2858, 1603, 1589, 1486, 1465, 1457, 1428, 1377, 1363, 1117, 1109, 1082, 1054, 1008, 997, 964, 908, 878, 850, 803, 783, 730, 715, 695, 657 cm^–1^. HRMS (ESI+) calcd for C_26_H_31_OSi, (M + H) 387.2139; found, 387.2126.

### 5-butyl-3-(2-methoxyphenyl)-2,2-diphenyl-1,2-oxasilolane

Prepared from (*E*)-1-(3-methoxyphenyl)­hept-1-en-3-ol (50 mg) according to general procedure B. The oxasilolane product (28 mg, 30%) was obtained as a colorless oil. S. data for the major diastereomer: ^1^H NMR (500 MHz, CDCl_3_) δ: 7.81 – 7.77 (m, 2H), 7.51 – 7.43 (m, 7H), 7.10 – 7.02 (m, 4H), 6.66 (dd, *J* = 8.1, 1.2 Hz, 1H), 4.23 (dtd, *J* = 10.2, 6.2, 3.6 Hz, 1H), 3.36 (s, 3H), 3.25 (dd, *J* = 13.3, 6.6 Hz, 1H), 2.51 (ddd, *J* = 12.5, 6.9, 3.6 Hz, 1H), 2.02 (td, *J* = 13.0, 11.0 Hz, 1H), 1.83 – 1.71 (m, 2H), 1.49 – 1.38 (m, 4H), 0.97 (t, *J* = 7.2 Hz, 3H). S. data for the major and minor diastereomer: ^13^C­{^1^H} NMR (126 MHz, CDCl_3_) δ: 156.0, 155.8, 135.2, 134.9, 134.7, 134.7, 134.6, 134.6, 134.4, 130.3, 130.0, 129.8, 129.8, 129.7, 129.6, 129.4, 128.1, 127.8, 127.7, 127.6, 127.6, 127.3, 127.0, 126.2, 125.6, 125.4, 124.8, 120.5, 120.4, 109.1, 108.9, 78.3, 77.6, 54.2, 53.9, 38.0, 37.9, 36.8, 35.1, 28.4, 28.2, 27.5, 25.7, 22.9, 22.8, 14.2, 14.1. IR (ATR): 3069, 2954, 2927, 2856, 1590, 1490, 1457, 1428, 1238, 1116, 1090, 1027, 997, 916, 889, 853, 814, 783, 738, 712, 695 cm^–1^. HRMS (ESI+) calcd for C_26_H_31_O_2_Si, (M + H) 403.2081; found, 403.2087.

### Conformational Analysis by DFT

Geometries of gas-phase molecules were optimized and structural energies calculated using density functional theory (DFT), the ORCA 4.1 software package, the PBE functional (a generalized gradient approximation), and the def2-TZVP basis set. For each diphenyl- and dimethylsilyl 3,6-substituted oxasilolane studied, four conformations were compared: (1) 3-axial/6-equatorial, (2) 3-equatorial/6-axial, (3) 3-equatorial/6-equatorial, (4) 3-axial/6-axial.

## Supplementary Material



## Data Availability

The data underlying this study are available in the published article and its Supporting Information.

## Notes

Copies of ^1^H NMR and ^13^C­{^1^H} spectra, and crystallographic data for compound **25** (PDF)
