# Supplementary material for: Synthesis of Oxasilolanes by TBAT-Catalyzed Hydroxyl-Directed Hydrosilylation
Source: J Org Chem. 2025 Aug 26;90(35):12334–45. doi: 10.1021/acs.joc.5c01496 (PMC12418312; doi:10.1021/acs.joc.5c01496)
Supplement: Supplementary file 1 [file jo5c01496_si_001.pdf]

Supporting Information For:

Synthesis of oxasilolanes by TBAT-catalyzed hydroxyl-directed hydrosilylation

Tess Q. Billmire,[a] Adam P. Jones,[a] Sarah M. Maffett,[a] Robert F. Berger,[a] Claire Gervais,[b] Werner Kaminsky,[b] and Gregory W. O'Neil[a,*]

[a]Department of Chemistry, Western Washington University, Bellingham, WA 98229 (USA)

[b]Department of Chemistry, University of Washington, Seattle, WA 98195 (USA)

*Corresponding author. Email: oneilg@wwu.edu

Contents:

NMR Spectra for Compound:	Page
5-methyl-2,2,3-triphenyl-1,2-oxasilolane (**2**)	S2
5-butyl-2,2,3-triphenyl-1,2-oxasilolane (**7**)	S3
5-isopropyl-2,2,3-triphenyl-1,2-oxasilolane (**8**)	S4
5-(tert-butyl)-2,2,3-triphenyl-1,2-oxasilolane (**9**)	S5
4,5-dimethyl-2,2,3-triphenyl,1-2,oxasiolane (**10**)	S6
3,5-dimethyl-2,2,3-triphenyl,1-2,oxasiolane (**11**)	S7
2-methyl-1-phenylbutane-1,3-diol (**12**)	S8
4-phenylpentan-2-ol (**13**)	S8
5-allyl-2,2,3-triphenyl-1,2-oxasilolane (**18**)	S10
(*E*)-1-(furan-2-yl)hept-1-en-3-ol (**19**)	S11
(6*E*,8*E*)-deca-6,8-dien-5-ol (**20**)	S12
5-butyl-3-(furan-2-yl)-2,2-diphenyl-1,2-oxasilolane (**21**)	S13
(*E*)-5-butyl-2,2-diphenyl-3-(prop-1-en-1-yl)-1,2-oxasilolane (**22**)	S14
6-butyl-2,2,3-triphenyl-1,2-oxasilinane (**23**)	S15
6-isopropyl-2,2,3-triphenyl-1,2-oxasilinane (**24**)	S16
6-(tert-butyl)-2,2,3-triphenyl-1,2-oxasilinane (**25**)	S17
2,2,3,6-tetraphenyl-1,2-oxasilinane (**26**)	S18
(*Z*)-1-phenyloct-1-en-4-ol (**27**)	S19
(*E*)-1-phenylnon-1-en-5-ol (**29**)	S20
(*E*)-2-methyl-1-phenyloct-1-en-4-ol (**30**)	S21
(*E*)-2-phenylnon-2-en-5-ol (**31**)	S22
6-butyl-3-methyl-2,2,3-triphenyl-1,2-oxasilinane (**32**)	S23
6-butyl-4-methyl-2,2,3-triphenyl-1,2-oxasilinane (**33**)	S24
5-butyl-3-(4-fluorophenyl)-2,2-diphenyl-1,2-oxasilolane	S25
3-(4-chlorophenyl)-5-isopropyl-2,2-diphenyl-1,2-oxasilolane	S26
3-(4-bromophenyl)-5-butyl-2,2-diphenyl-1,2-oxasilolane	S27
5-butyl-2,2-diphenyl-3-(p-tolyl)-1,2-oxasilolane	S28
5-butyl-3-(4-methoxyphenyl)-2,2-diphenyl-1,2-oxasilolane	S29

5-butyl-2,2-diphenyl-3-(m-tolyl)-1,2-oxasilolane	S30
5-butyl-3-(2-methoxyphenyl)-2,2-diphenyl-1,2-oxasilolane	S31
Additive Screening Results for Hydrosilylation of **24**	S32
Crystallographic data for the structure of compound **25**	S33
References	S35

General Information. NMR Spectra were recorded on a 500 MHz spectrometer in $CDCl_3$ or C_6D_6; chemical shifts (d) are given in ppm, coupling constants (*J*) in Hz. Solvent signals were used as references ($CDCl_3$: δ 77.0 ppm; residual $CHCl_3$ in $CDCl_3$: δ 7.26 ppm, C_6D_6: δ 128.06 ppm; residual C_6H_6 in C_6D_6 δ 7.16 ppm).

Copies of NMR Spectra:

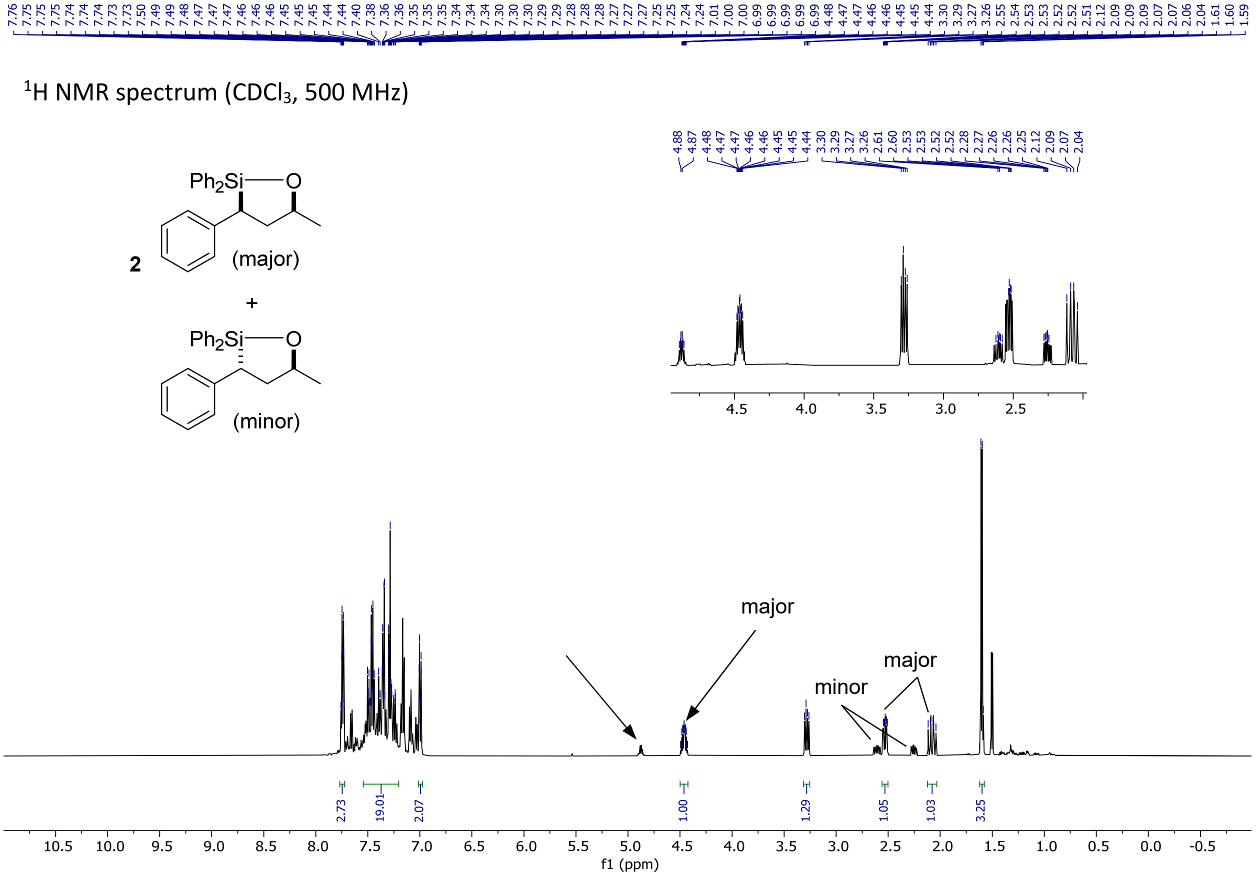

^{1}H NMR spectrum ($CDCl_3$, 500 MHz)

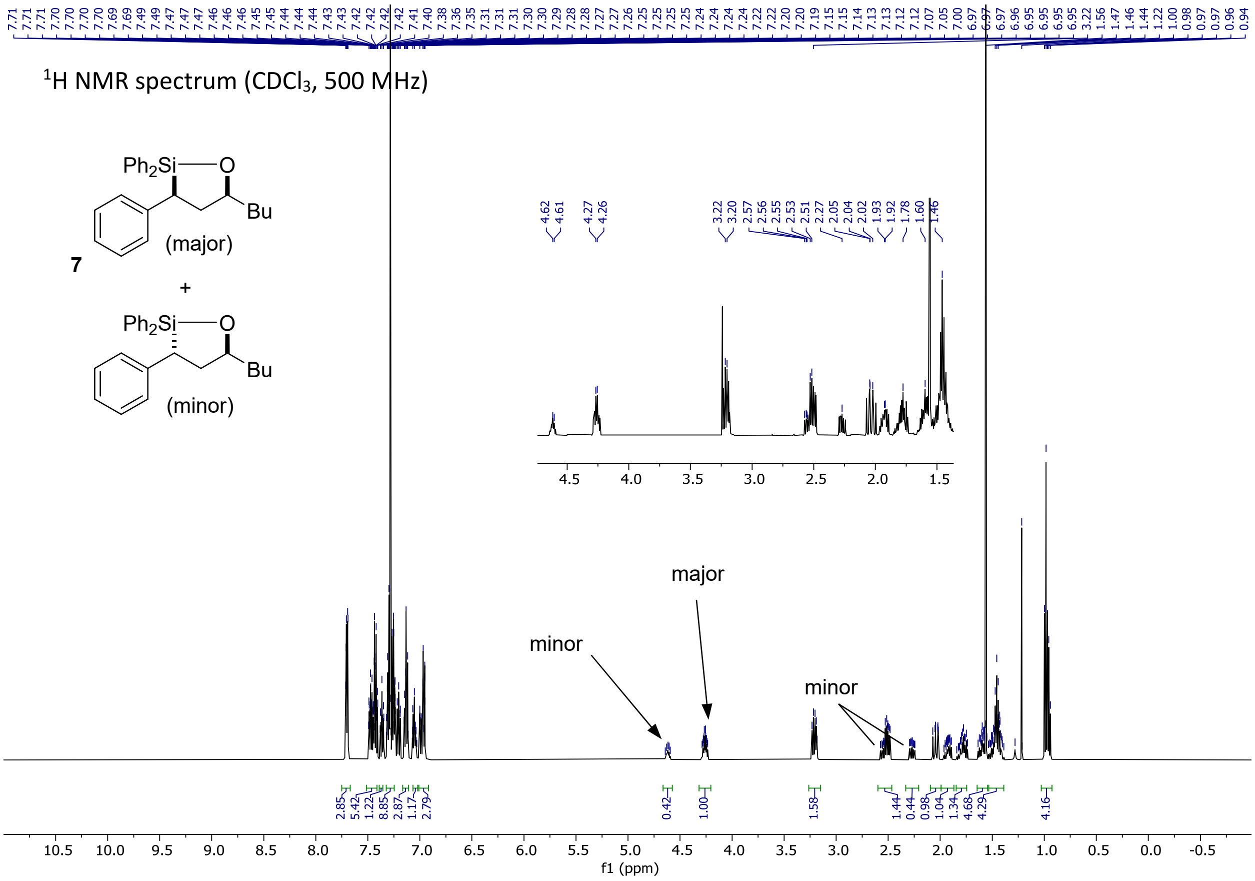

^{13}C{^{1}H} NMR spectrum ($CDCl_3$, 126 MHz)

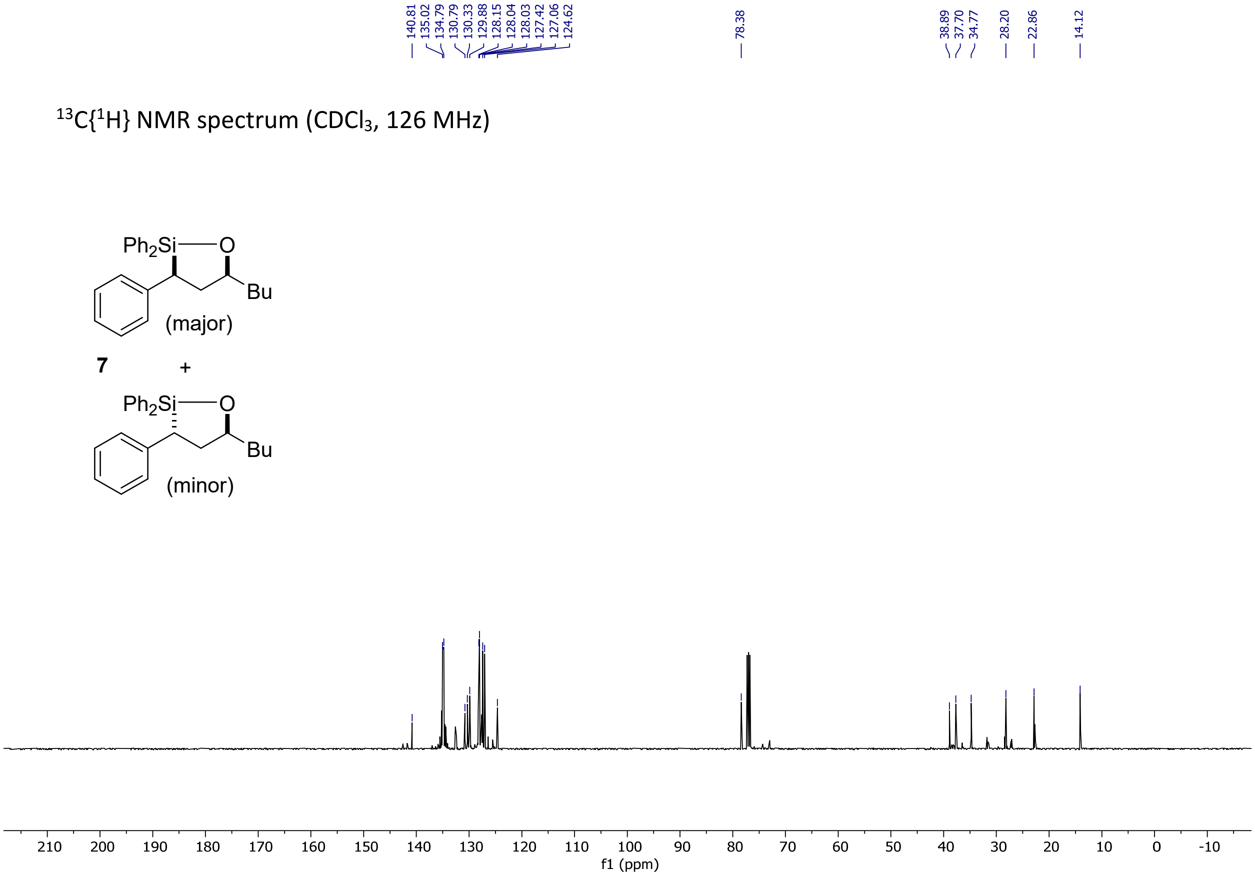

^{1}H NMR spectrum ($CDCl_3$, 500 MHz)

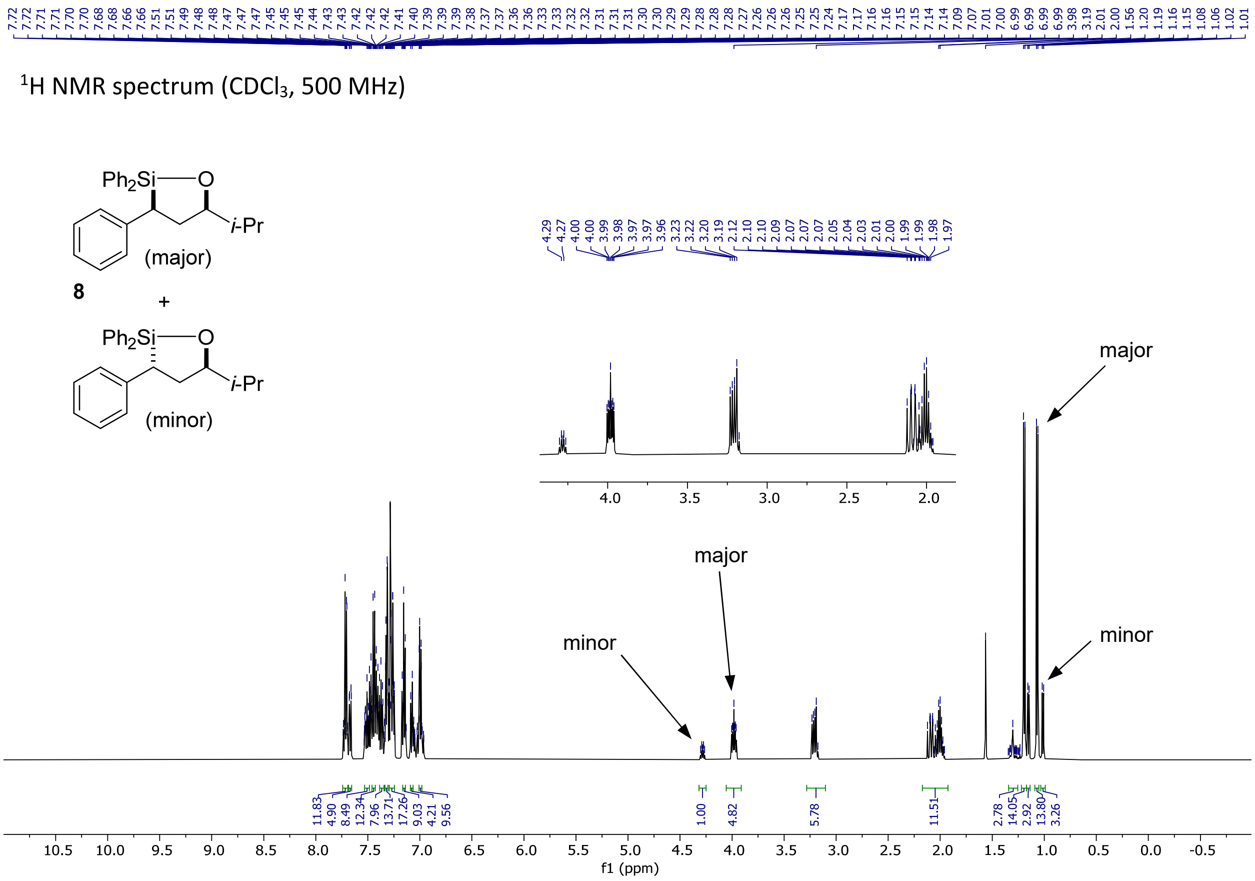

^{13}C{^{1}H} NMR spectrum ($CDCl_3$, 126 MHz)

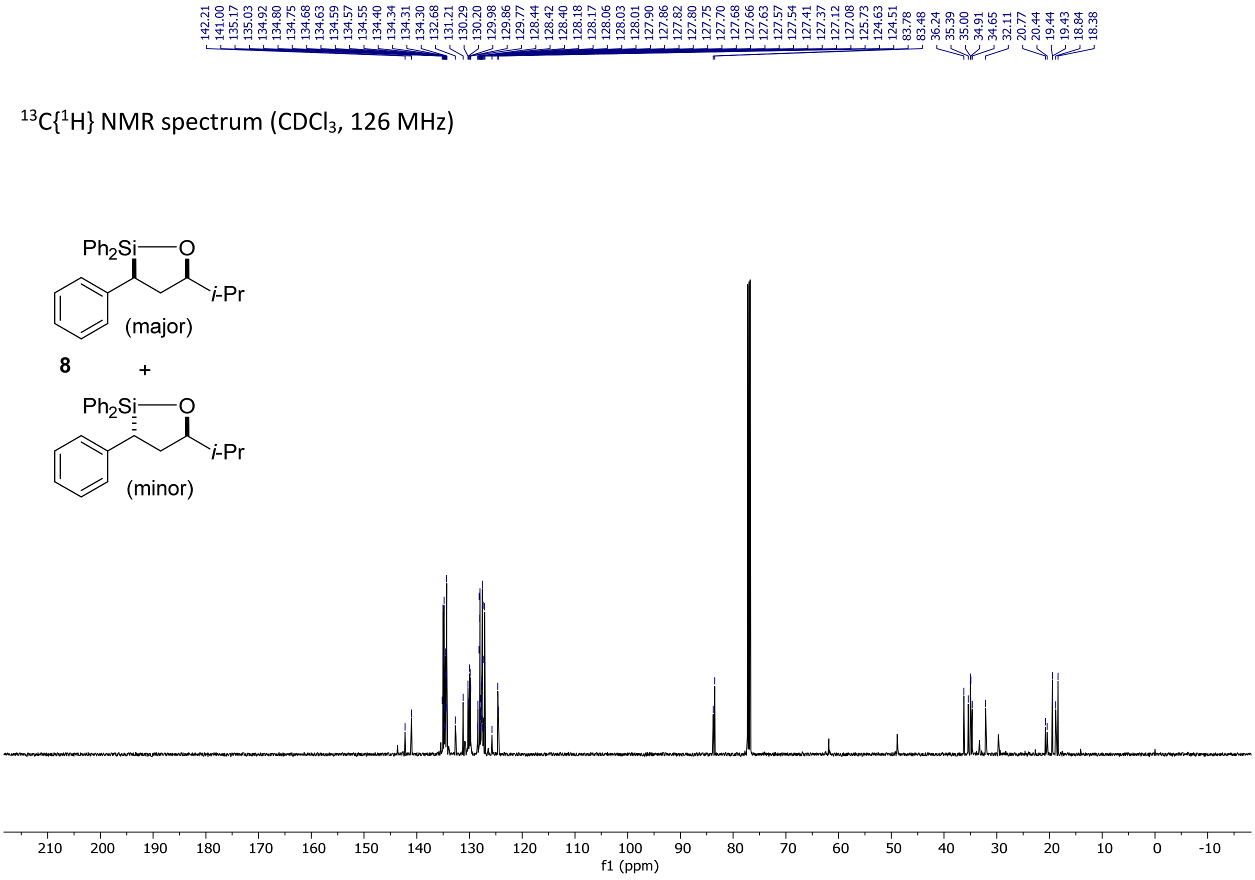

^{1}H NMR spectrum ($CDCl_3$, 500 MHz)

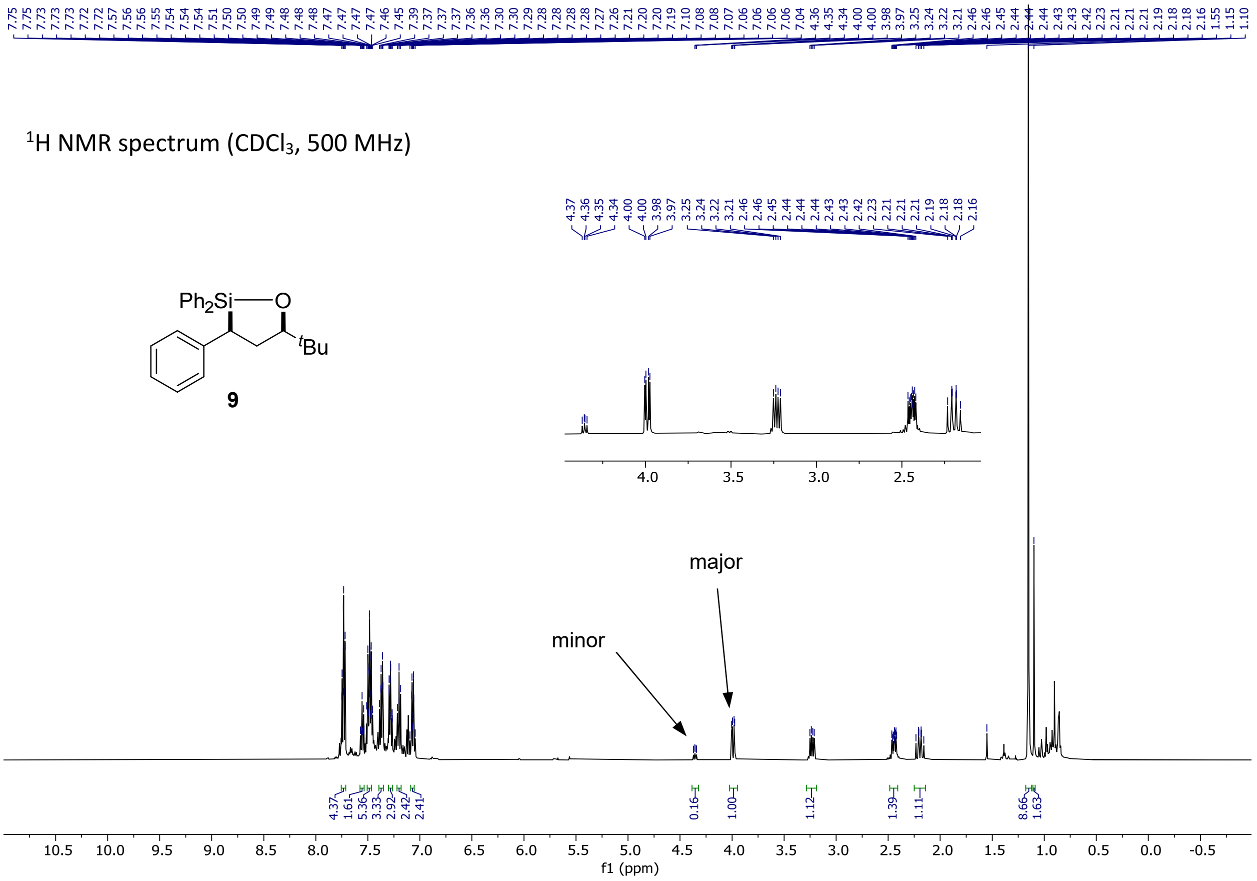

$^{13}C\{^{1}H\}$ NMR spectrum ($CDCl_3$, 126 MHz)

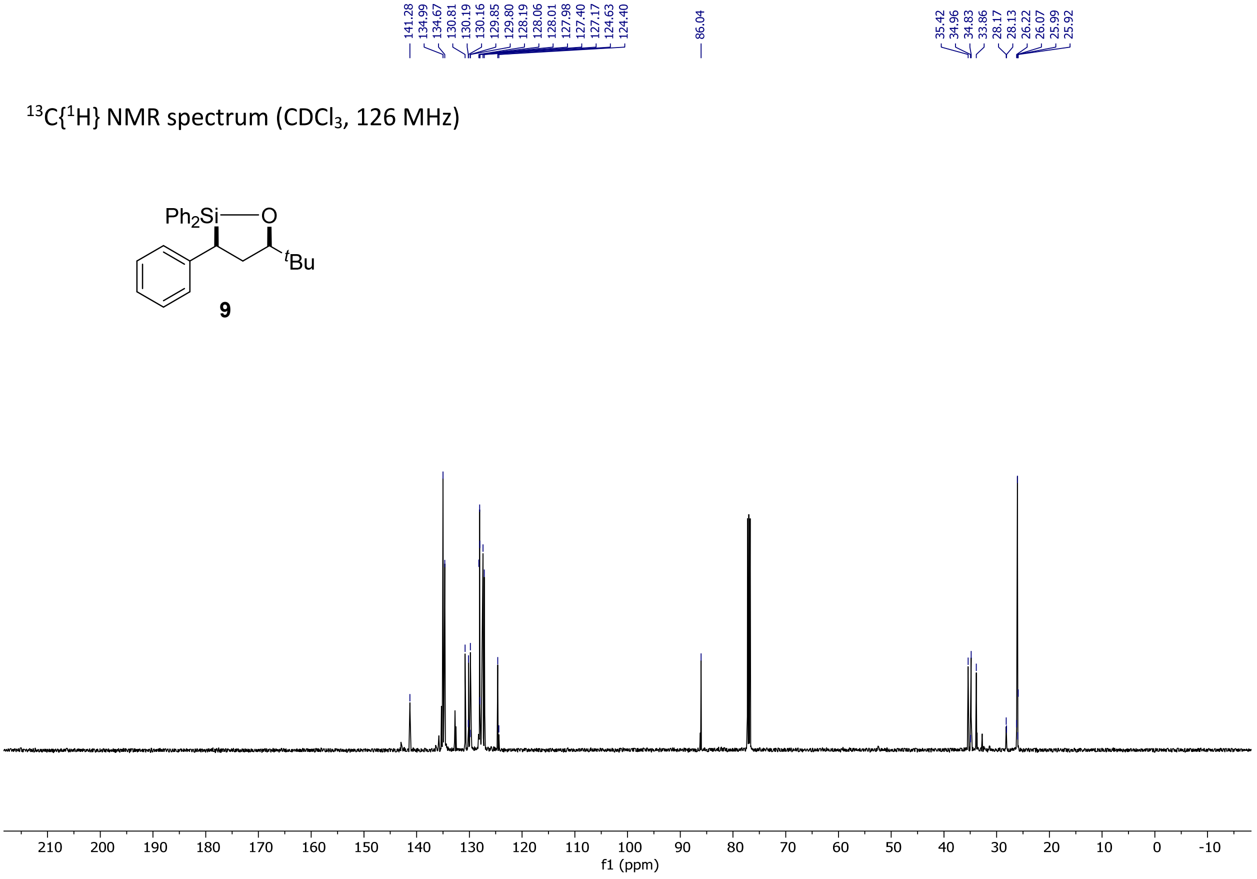

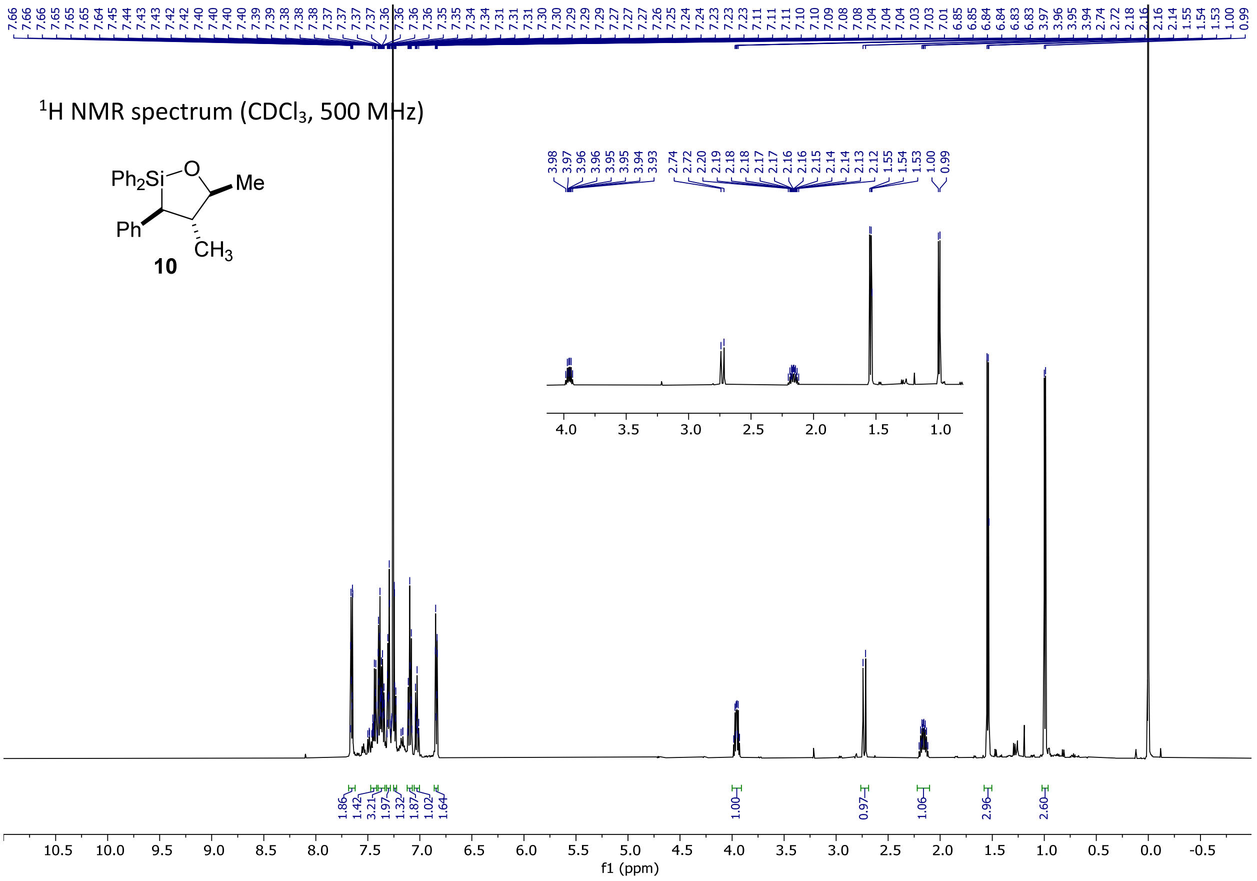

^{1}H NMR spectrum ($CDCl_3$, 500 MHz)
Ph$_2$Si O Me
Ph CH$_3$
10
1.86 1.42 3.21 1.97 1.32 1.87 1.02 1.64 1.00 0.97 1.06 2.96 2.60
f1 (ppm)

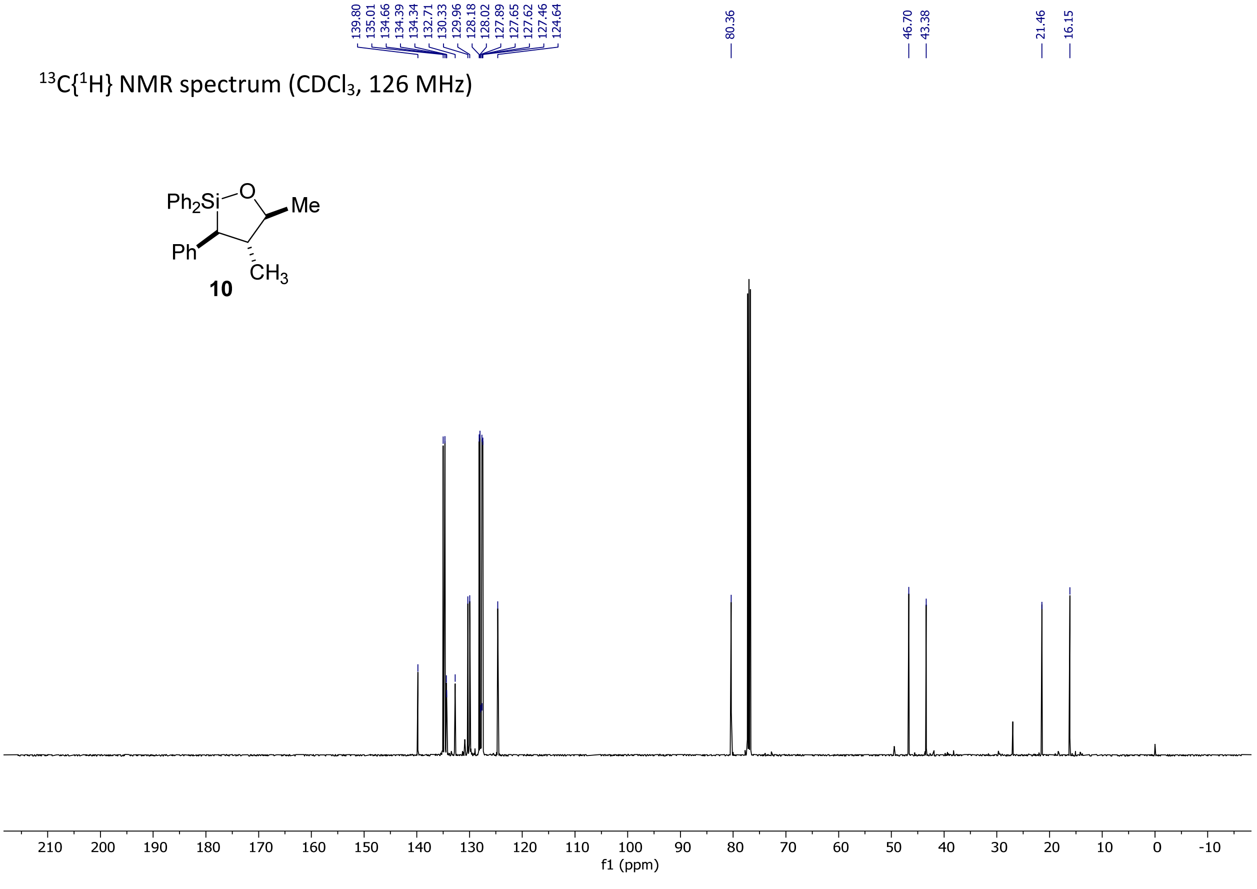

^{13}C{^{1}H} NMR spectrum ($CDCl_3$, 126 MHz)
139.80 135.01 134.66 134.39 134.34 132.71 130.33 129.96 128.18 128.02 127.89 127.65 127.62 127.46 124.64
80.36
46.70 43.38
21.46 16.15
Ph$_2$Si O Me
Ph CH$_3$
10
f1 (ppm)

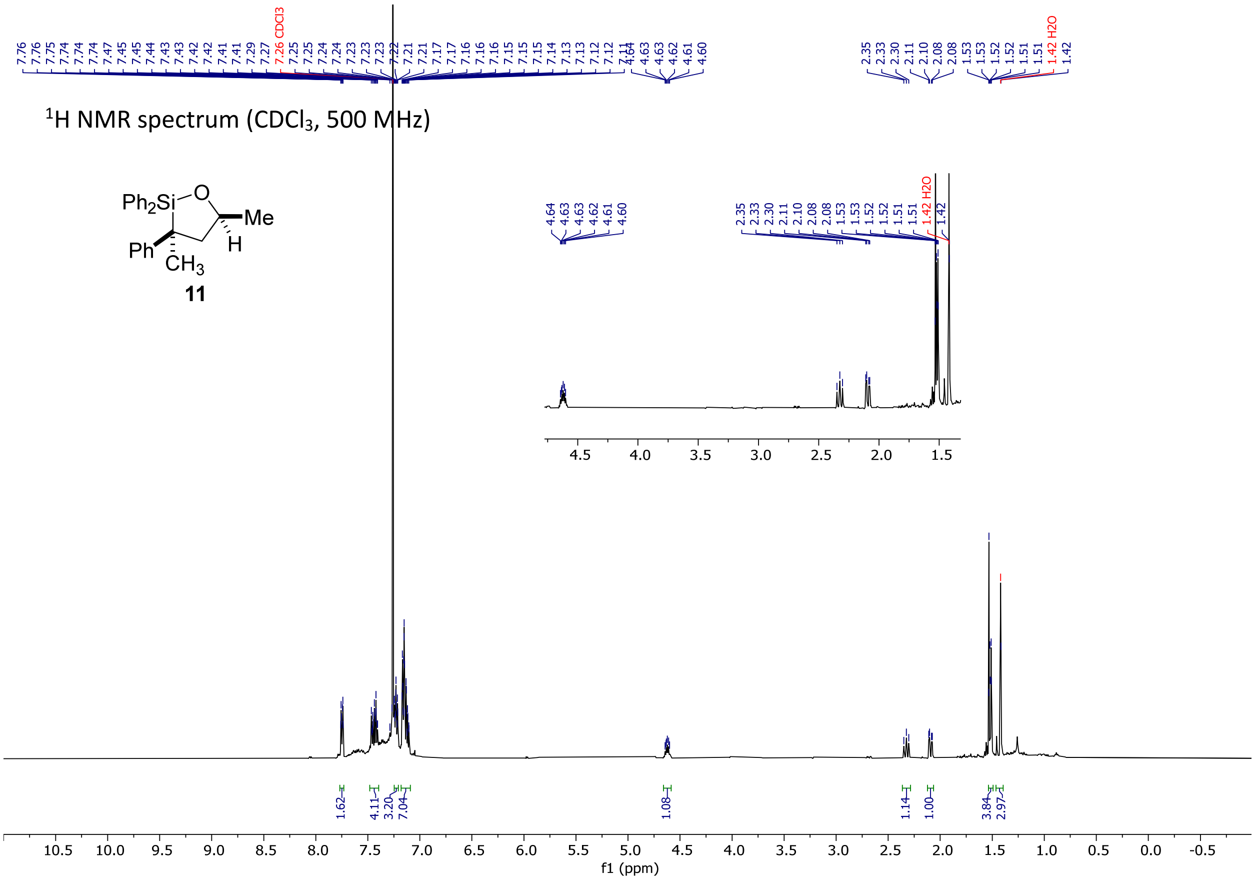

7.76 7.76 7.75 7.74 7.74 7.74 7.47 7.45 7.45 7.44 7.43 7.43 7.42 7.42 7.41 7.41 7.29 7.27 7.26 CDCl3 7.25 7.25 7.24 7.24 7.23 7.23 7.23 7.22 7.21 7.21 7.17 7.17 7.16 7.16 7.15 7.15 7.15 7.14 7.13 7.13 7.12 7.12 7.11 4.64 4.63 4.63 4.62 4.61 4.60
2.35 2.33 2.30 2.11 2.10 2.08 2.08 1.53 1.53 1.52 1.52 1.51 1.51 1.42 H2O 1.42
1H NMR spectrum (CDCl3, 500 MHz)
Ph2Si
O
Me
H
Ph
CH3
11
1.62 4.11 3.20 7.04 1.08 1.14 1.00 3.84 2.97
f1 (ppm)

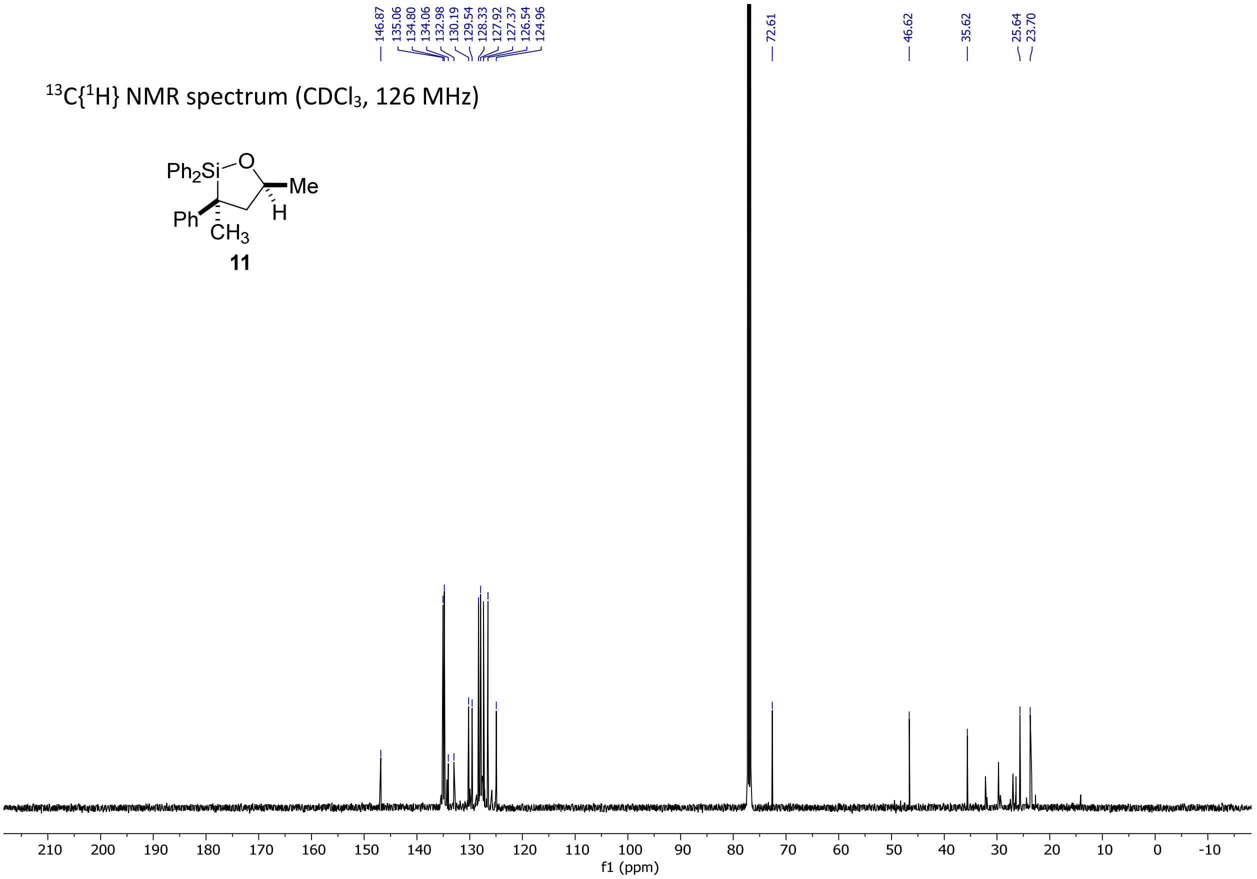

146.87 135.06 134.80 134.06 132.98 130.19 129.54 128.33 127.92 127.37 126.54 124.96
72.61 46.62 35.62 25.64 23.70
13C{1H} NMR spectrum (CDCl3, 126 MHz)
Ph2Si
O
Me
H
Ph
CH3
11
f1 (ppm)

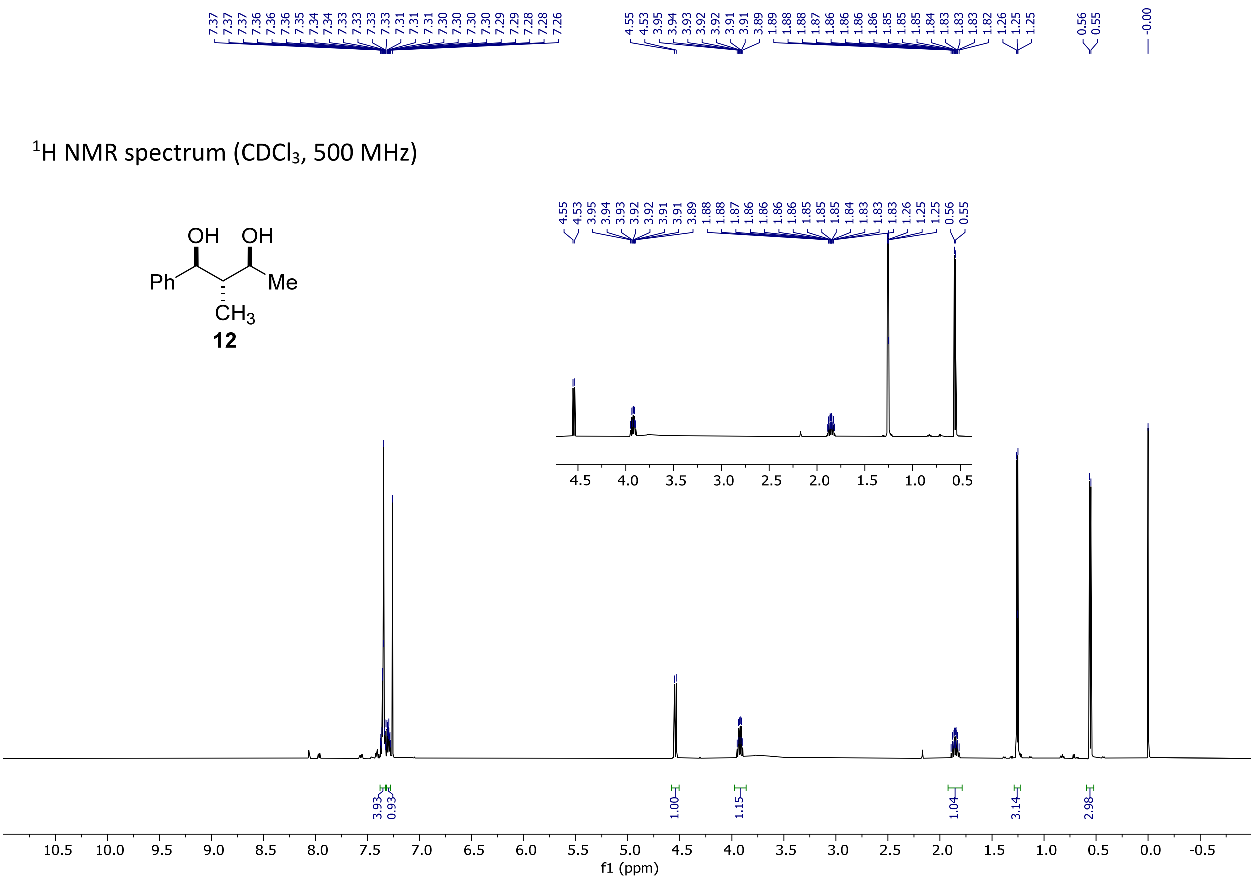

^{1}H NMR spectrum (CDCl$_3$, 500 MHz)
OH
OH
Ph
Me
CH$_3$
12
f1 (ppm)

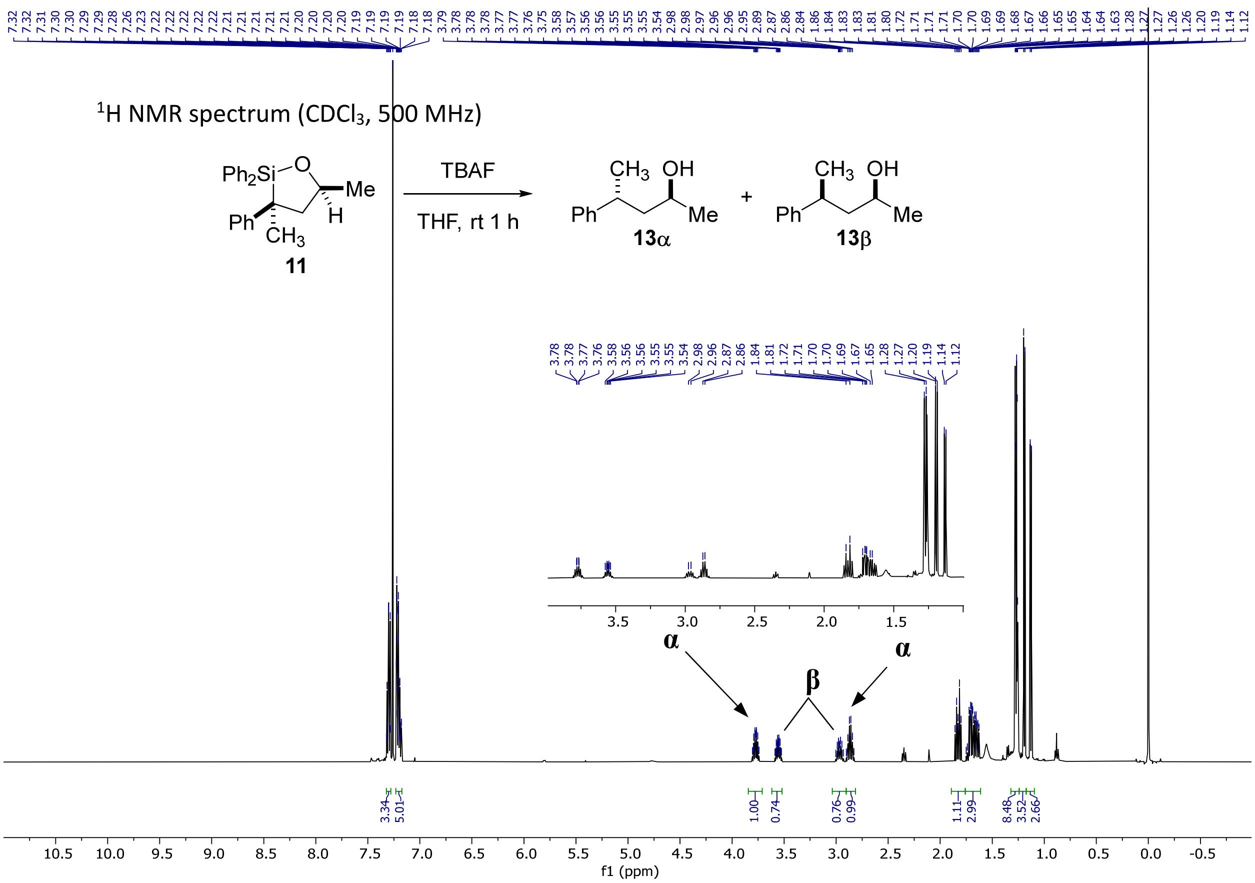

^{1}H NMR spectrum (CDCl$_3$, 500 MHz)
Ph$_2$Si
O
Me
H
Ph
CH$_3$
11
TBAF
THF, rt 1 h
CH$_3$
OH
Ph
Me
13α
+
CH$_3$
OH
Ph
Me
13β
α
β
α
f1 (ppm)

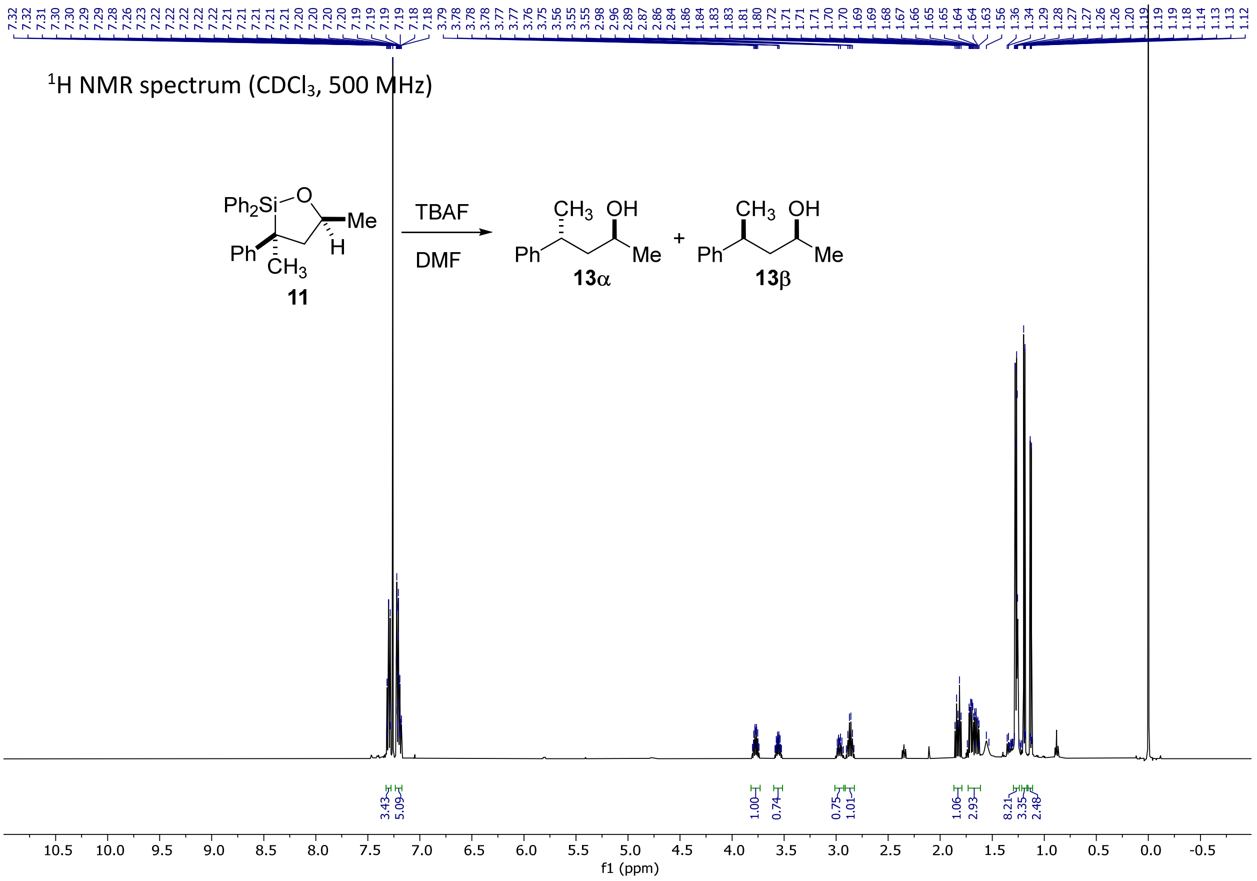

^{1}H NMR spectrum ($CDCl_3$, 500 MHz)
Ph2Si
O
Me
H
Ph
CH3
11
TBAF
DMF
CH3
OH
Ph
Me
13α
+
13β
f1 (ppm)

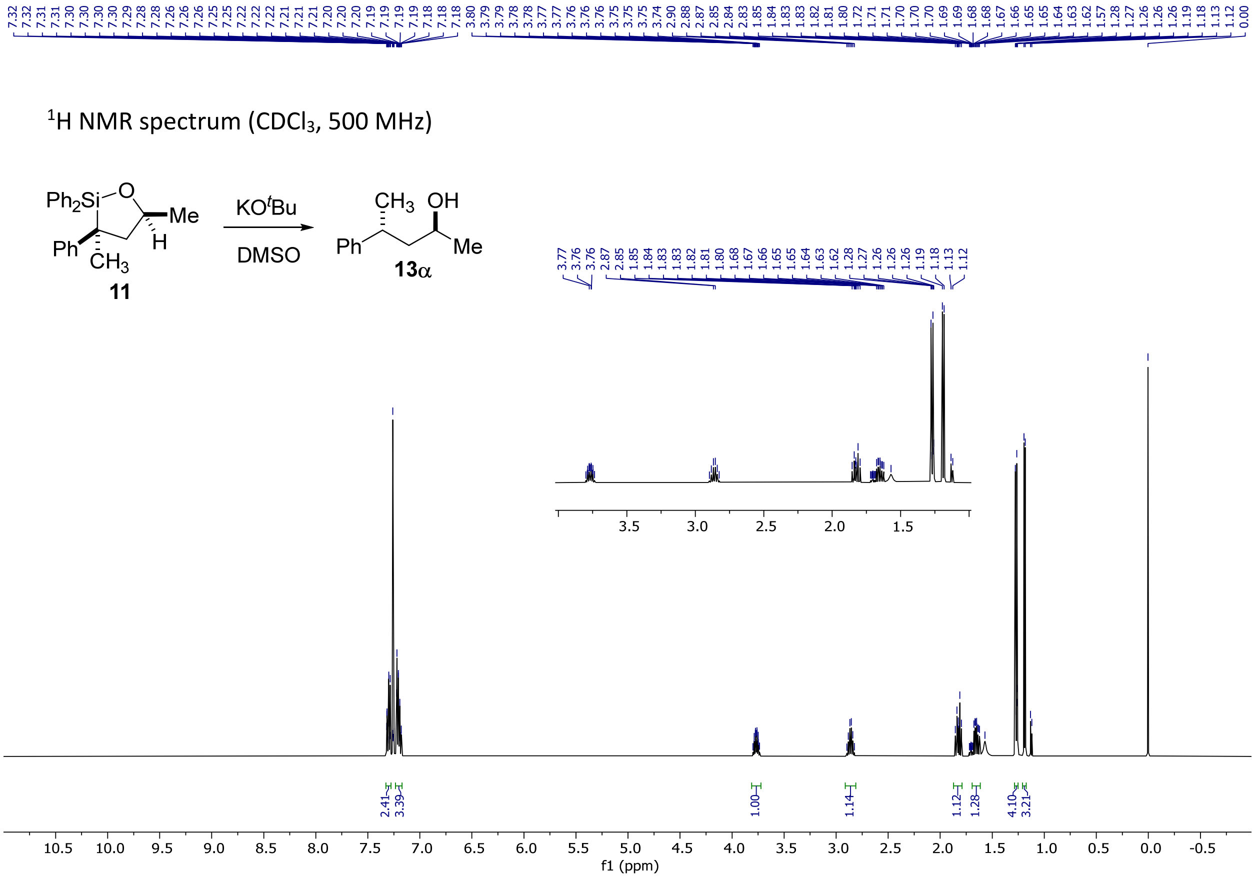

^{1}H NMR spectrum ($CDCl_3$, 500 MHz)
Ph2Si
O
Me
H
Ph
CH3
11
KOtBu
DMSO
CH3
OH
Ph
Me
13α
f1 (ppm)

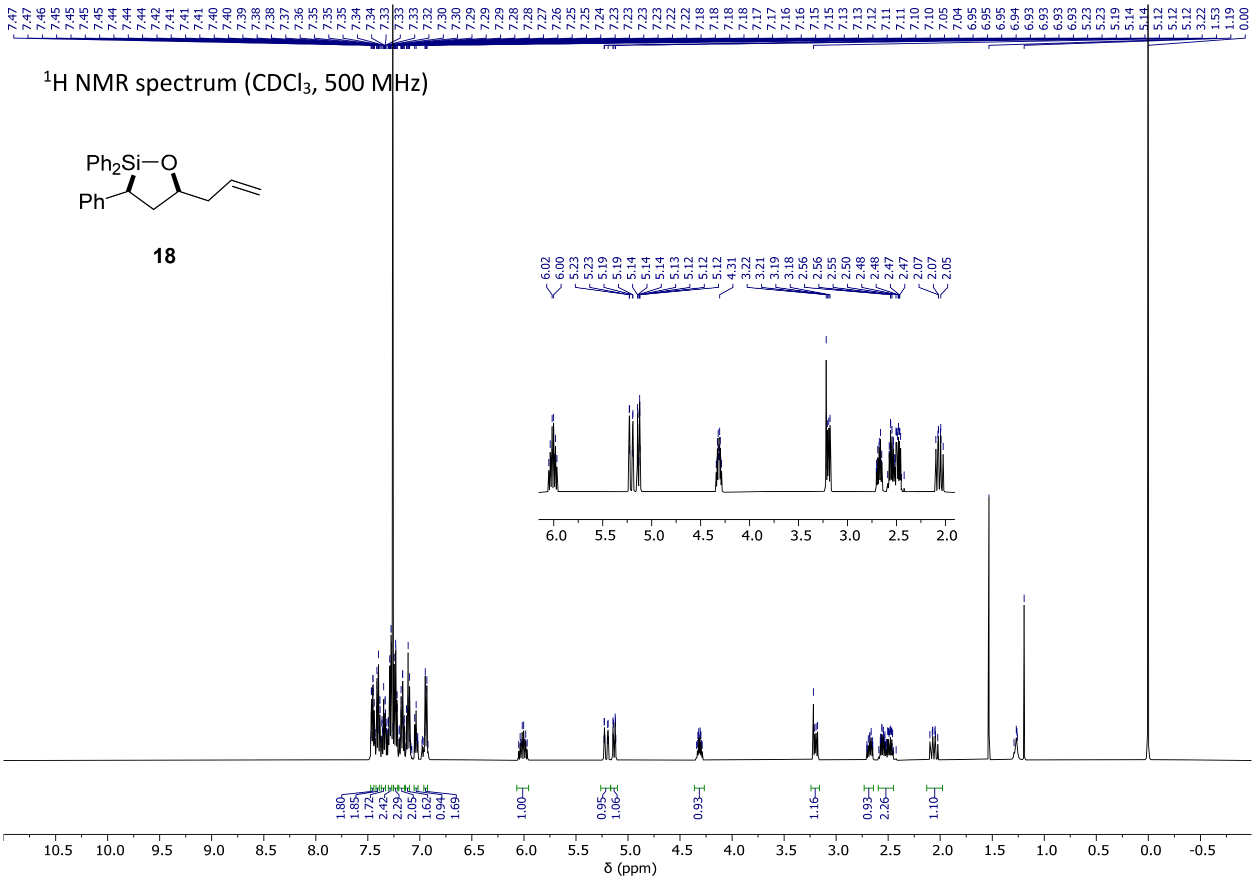

1H NMR spectrum (CDCl3, 500 MHz)
Ph2Si—O
Ph
18
δ (ppm)

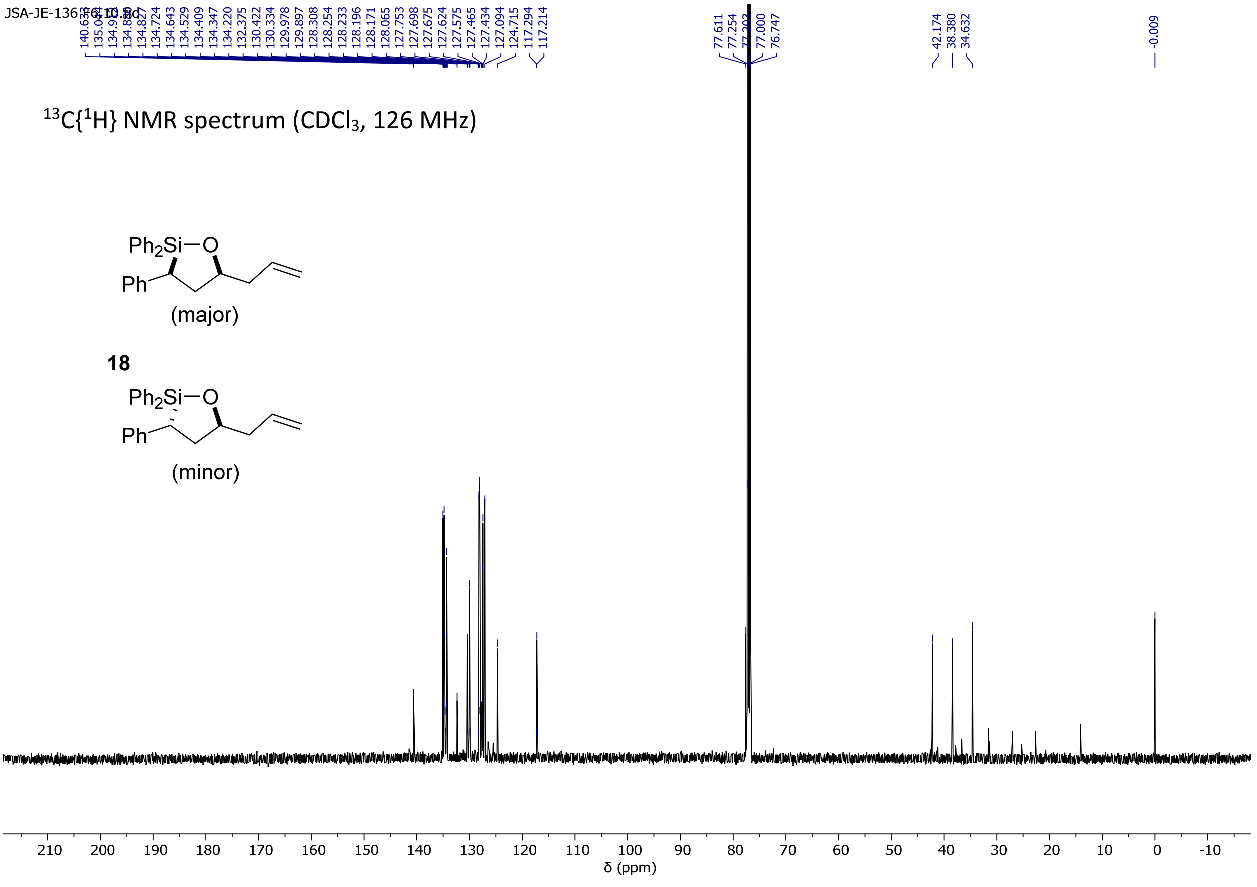

13C{1H} NMR spectrum (CDCl3, 126 MHz)
Ph2Si—O
Ph
(major)
18
Ph2Si—O
Ph
(minor)
δ (ppm)

^{1}H NMR spectrum ($CDCl_3$, 500 MHz)

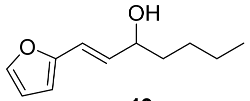

19

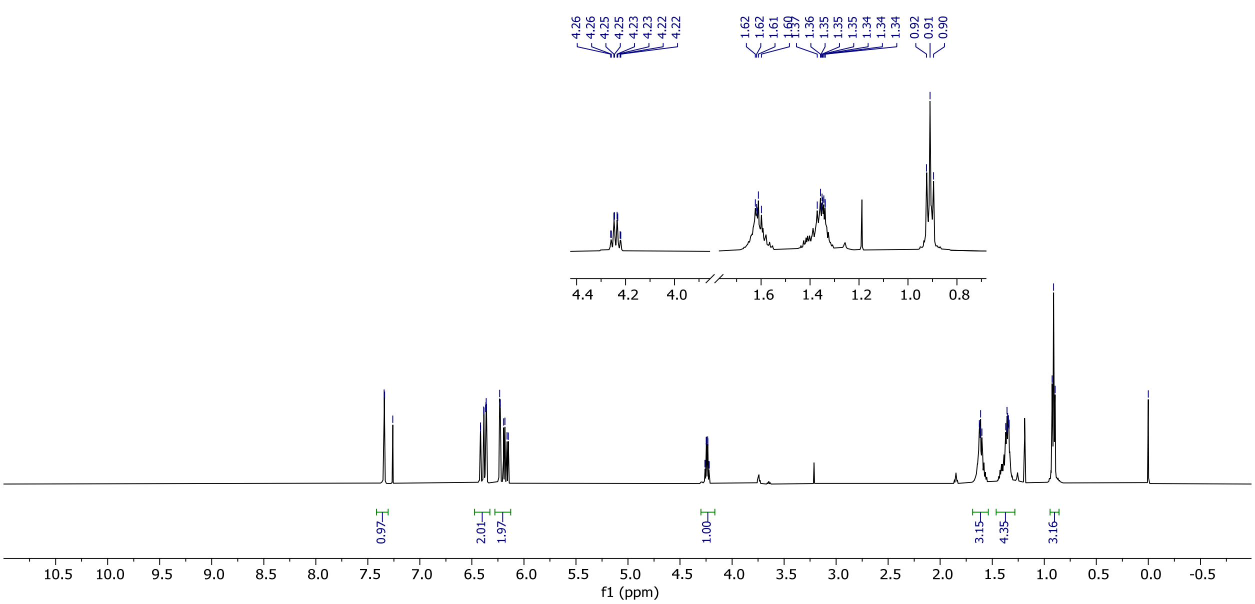

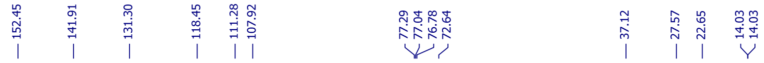

$^{13}C\{^{1}H\}$ NMR spectrum ($CDCl_3$, 126 MHz)

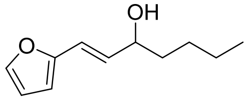

19

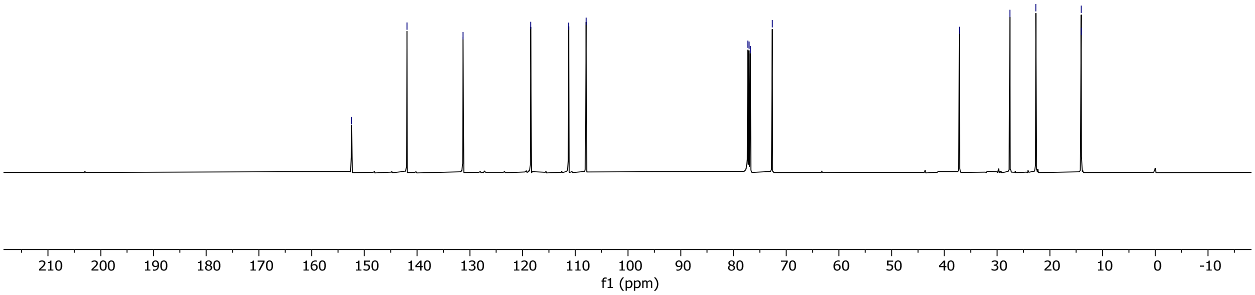

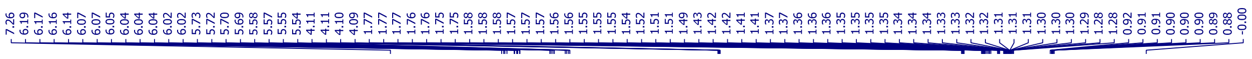

^{1}H NMR spectrum ($CDCl_3$, 500 MHz)

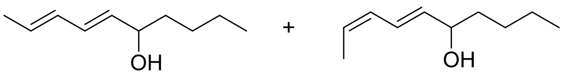

20

~5 : 1

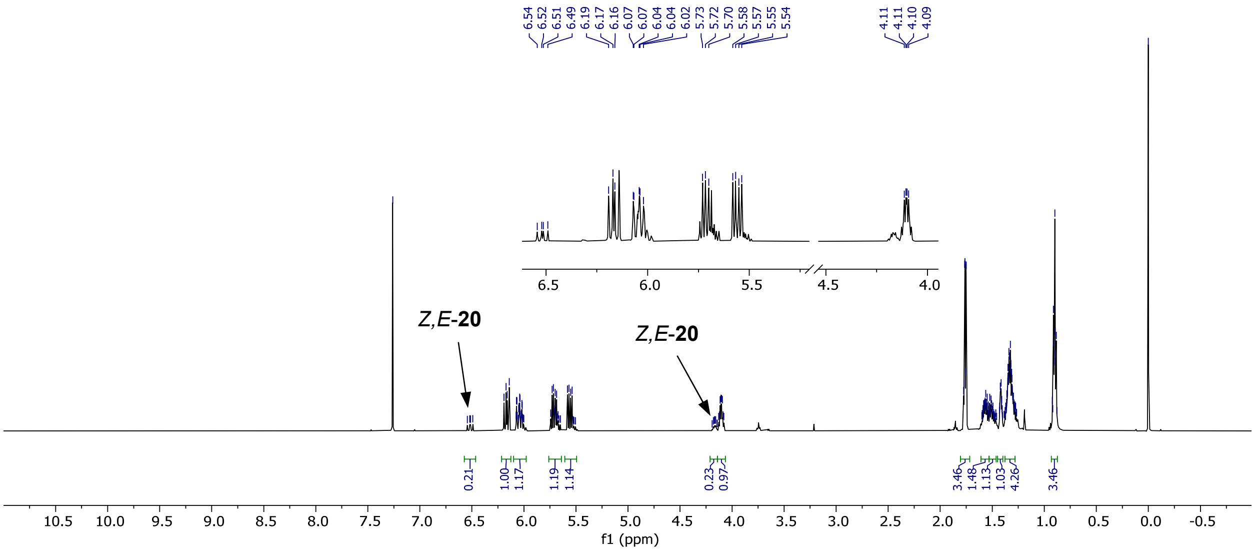

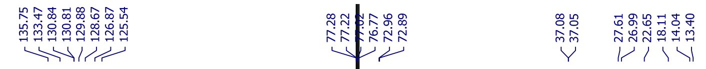

^{13}C{^{1}H} NMR spectrum ($CDCl_3$, 126 MHz)

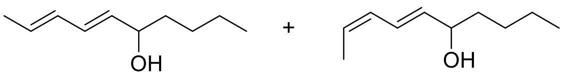

20

~5 : 1

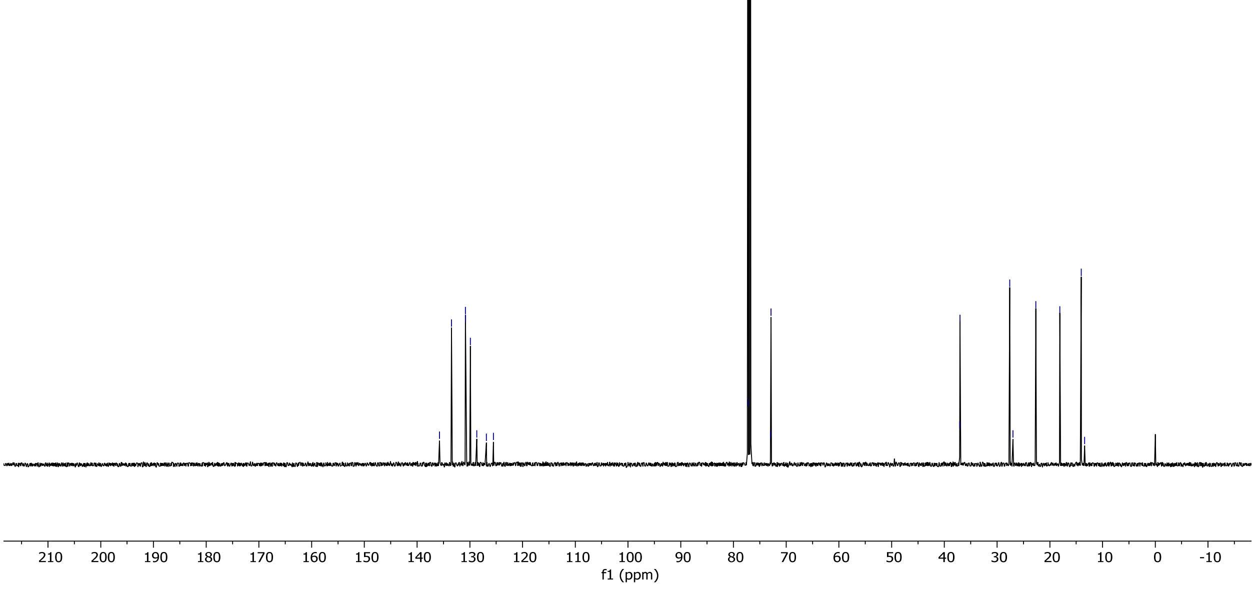

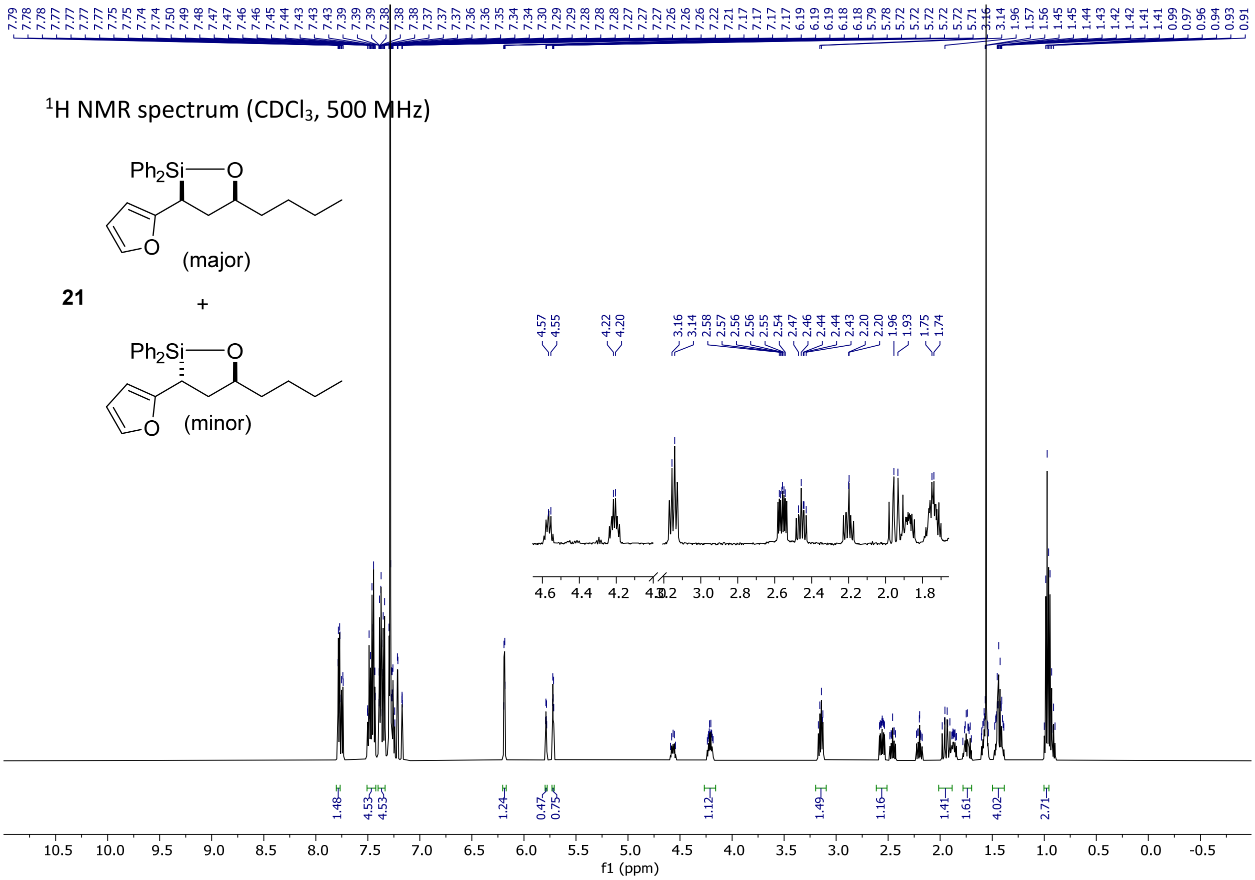
¹H NMR spectrum (CDCl₃, 500 MHz)
Ph₂Si—O
(major)
21
+
Ph₂Si—O
(minor)
f1 (ppm)

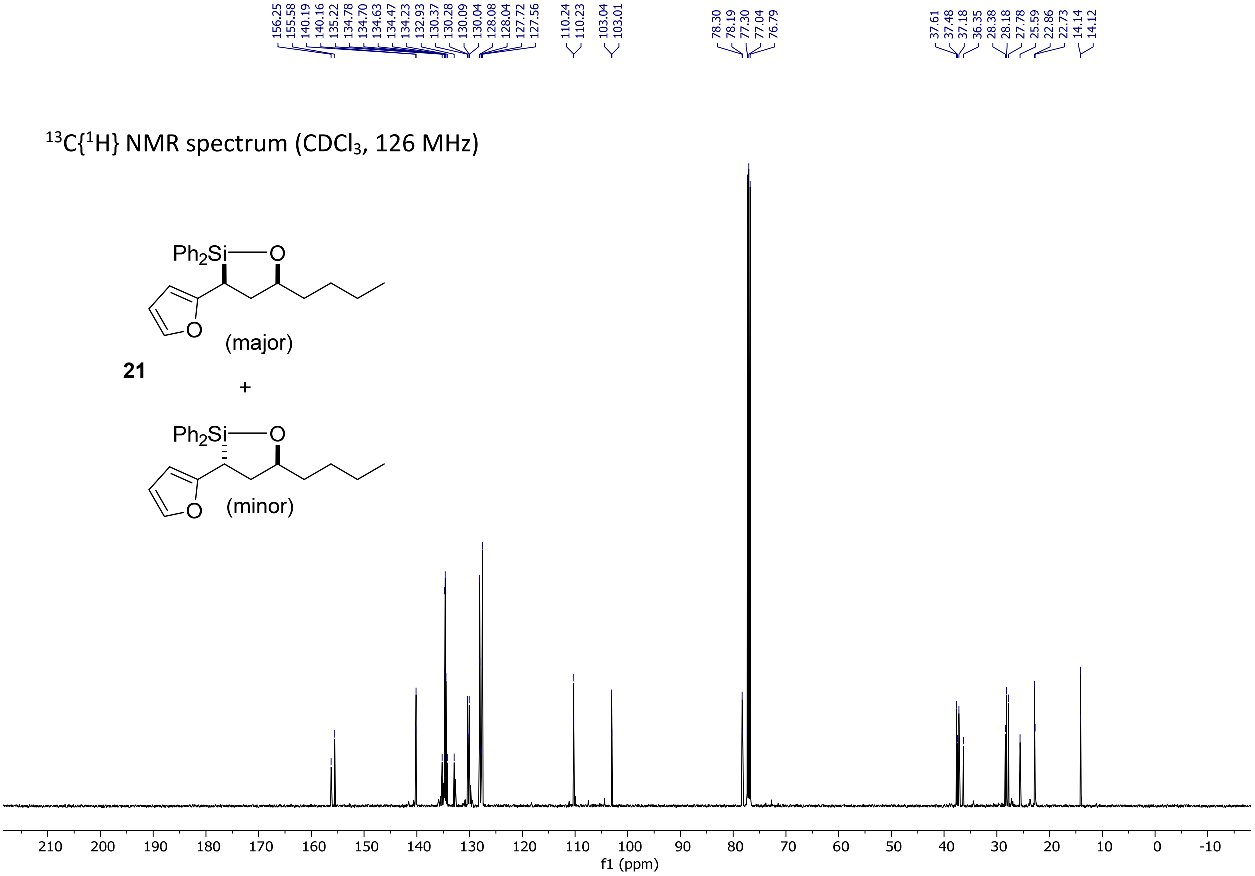
¹³C{¹H} NMR spectrum (CDCl₃, 126 MHz)
Ph₂Si—O
(major)
21
+
Ph₂Si—O
(minor)
f1 (ppm)

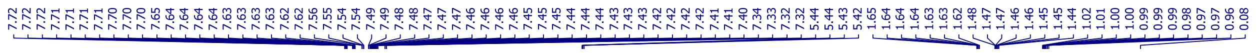

^{1}H NMR spectrum ($CDCl_3$, 500 MHz)

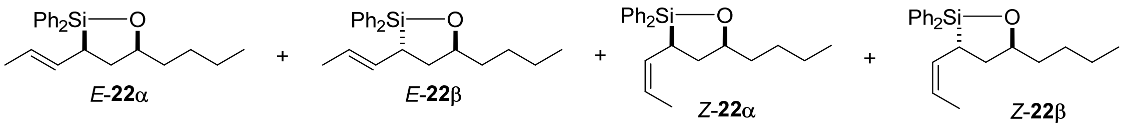

E-**22**α : *E*-**22**β : *Z*-**22**α : *Z*-**22**β
1.2 : 1 : 0.24 : 0.20

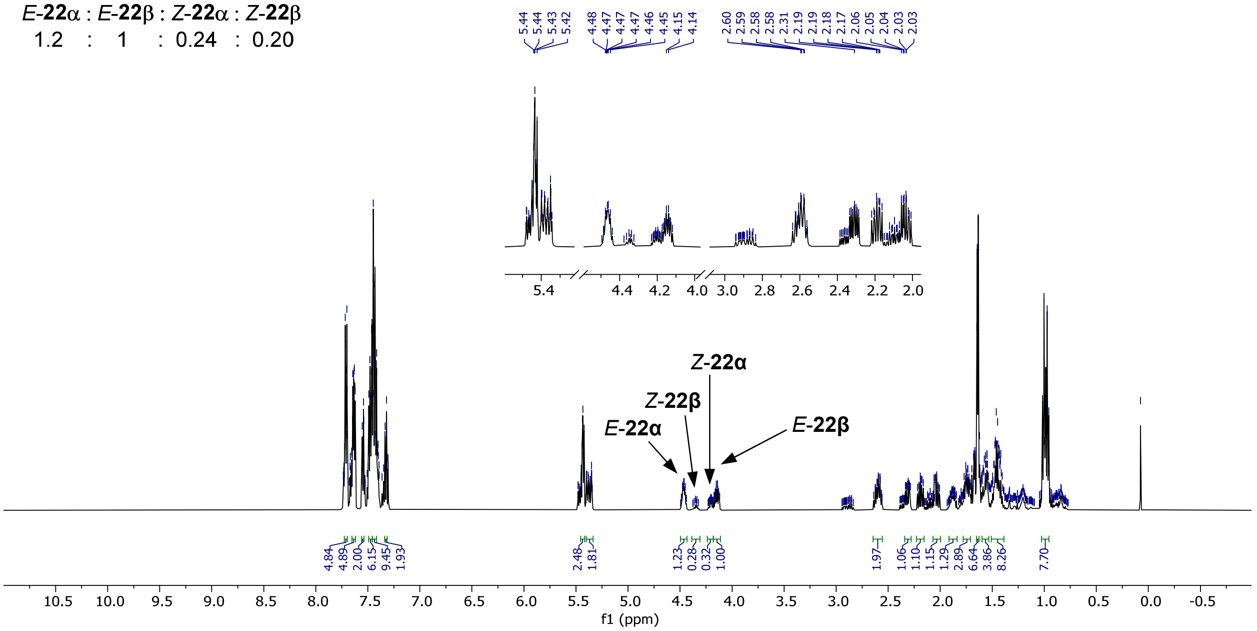

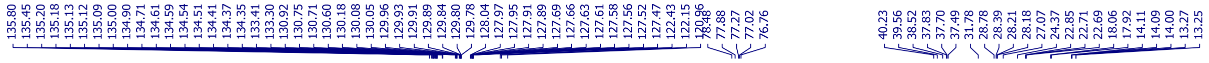

$^{13}C\{^{1}H\}$ NMR spectrum ($CDCl_3$, 126 MHz)

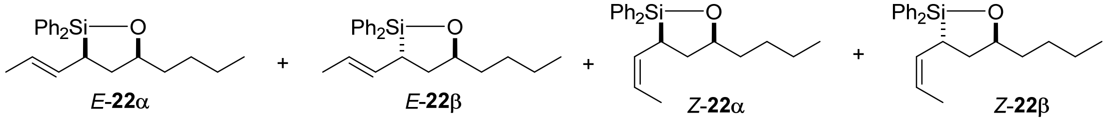

E-**22**α : *E*-**22**β : *Z*-**22**α : *Z*-**22**β = 1.2 : 1 : 0.24 : 0.20

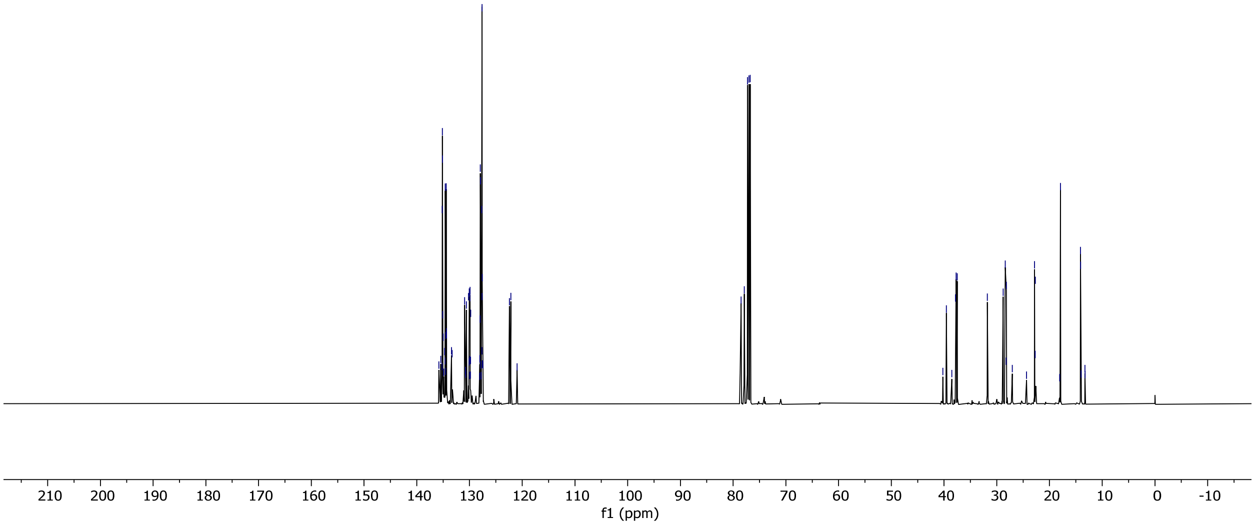

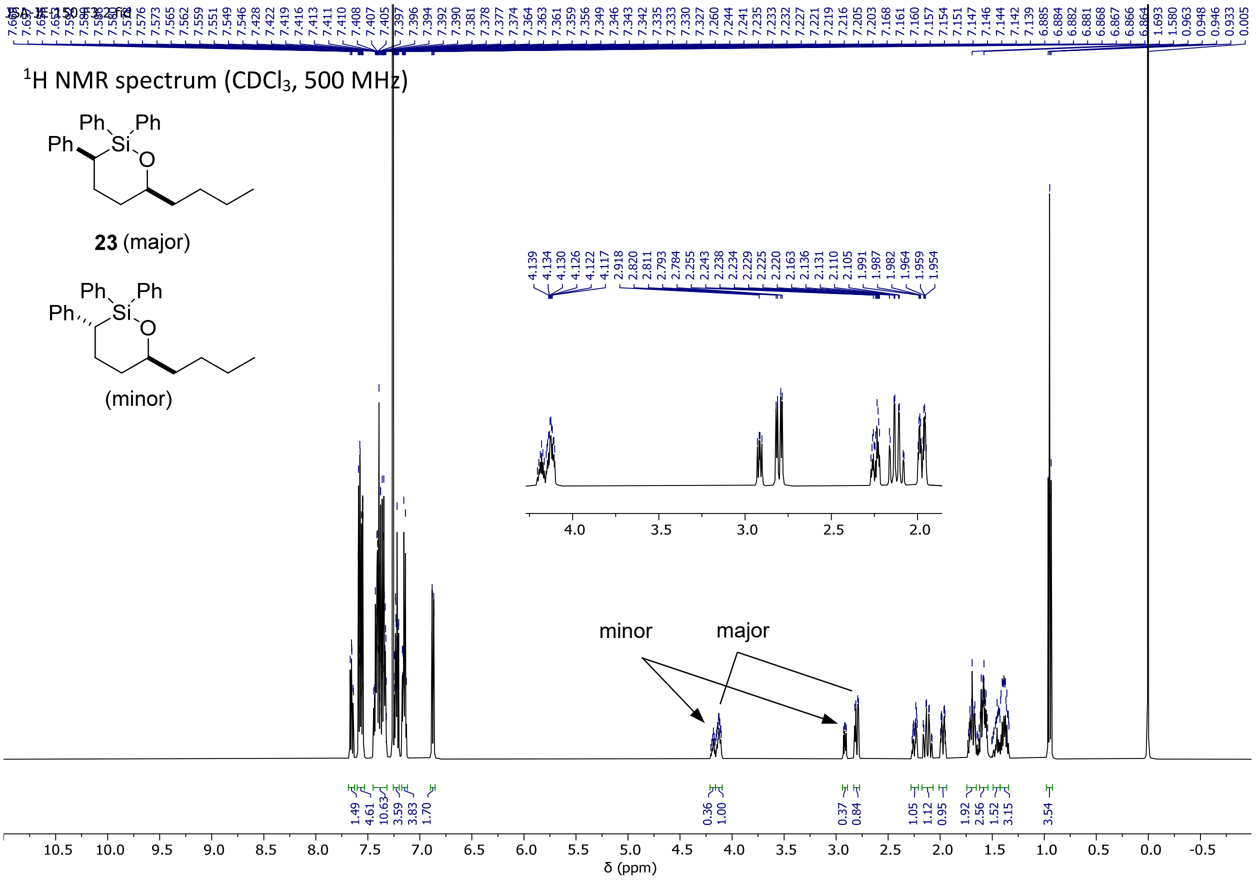
JSA-JE-150 F3.2.fid
1H NMR spectrum (CDCl3, 500 MHz)
Ph Ph Si O Ph
23 (major)
(minor)
minor
major
4.0 3.5 3.0 2.5 2.0
1.49 4.61 10.63 3.59 3.83 1.70 0.36 1.00 0.37 0.84 1.05 1.12 0.95 1.92 2.56 1.52 3.15 3.54
δ (ppm)

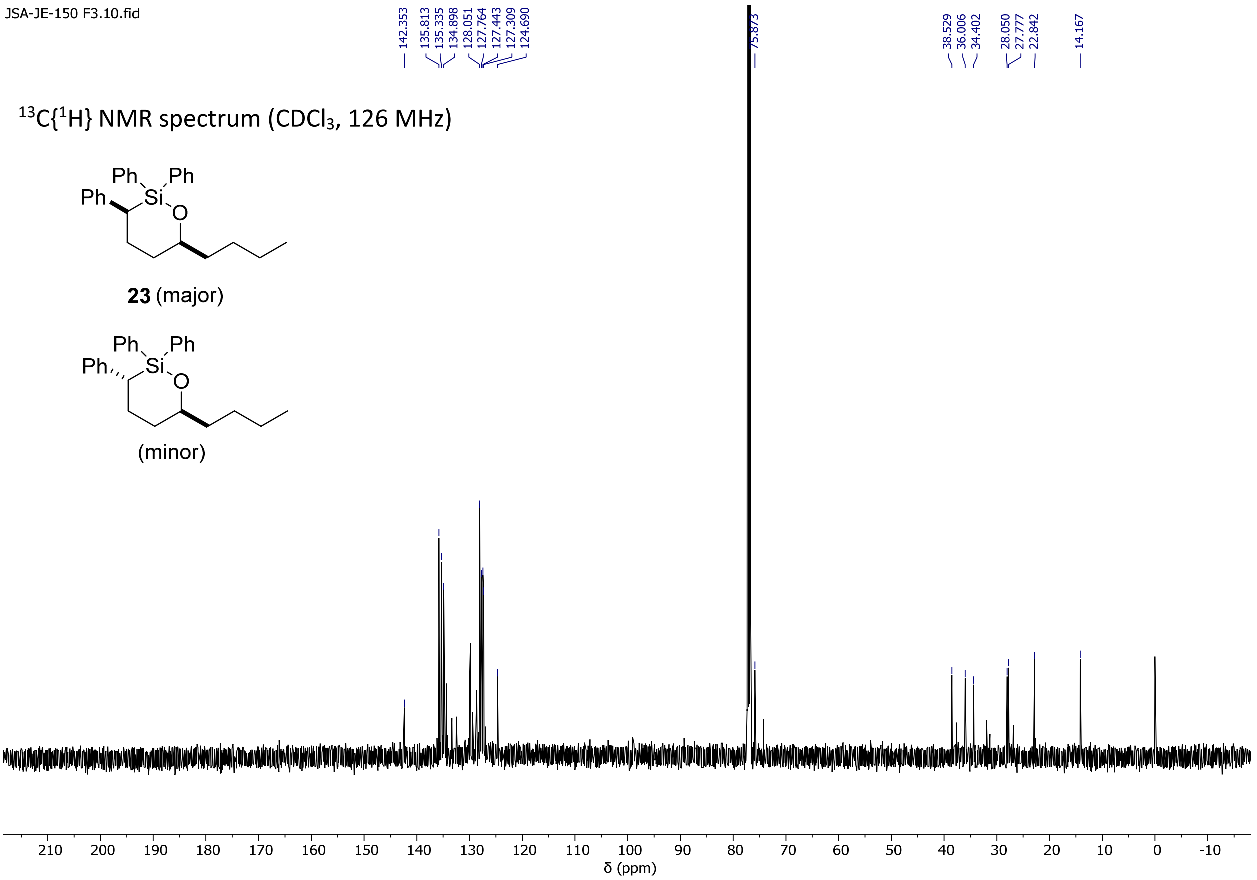
JSA-JE-150 F3.10.fid
142.353
135.813
135.335
134.898
128.051
127.764
127.443
127.309
124.690
75.873
38.529
36.006
34.402
28.050
27.777
22.842
14.167
13C{1H} NMR spectrum (CDCl3, 126 MHz)
Ph Ph Si O Ph
23 (major)
(minor)
δ (ppm)

^{1}H NMR spectrum ($CDCl_3$, 500 MHz)

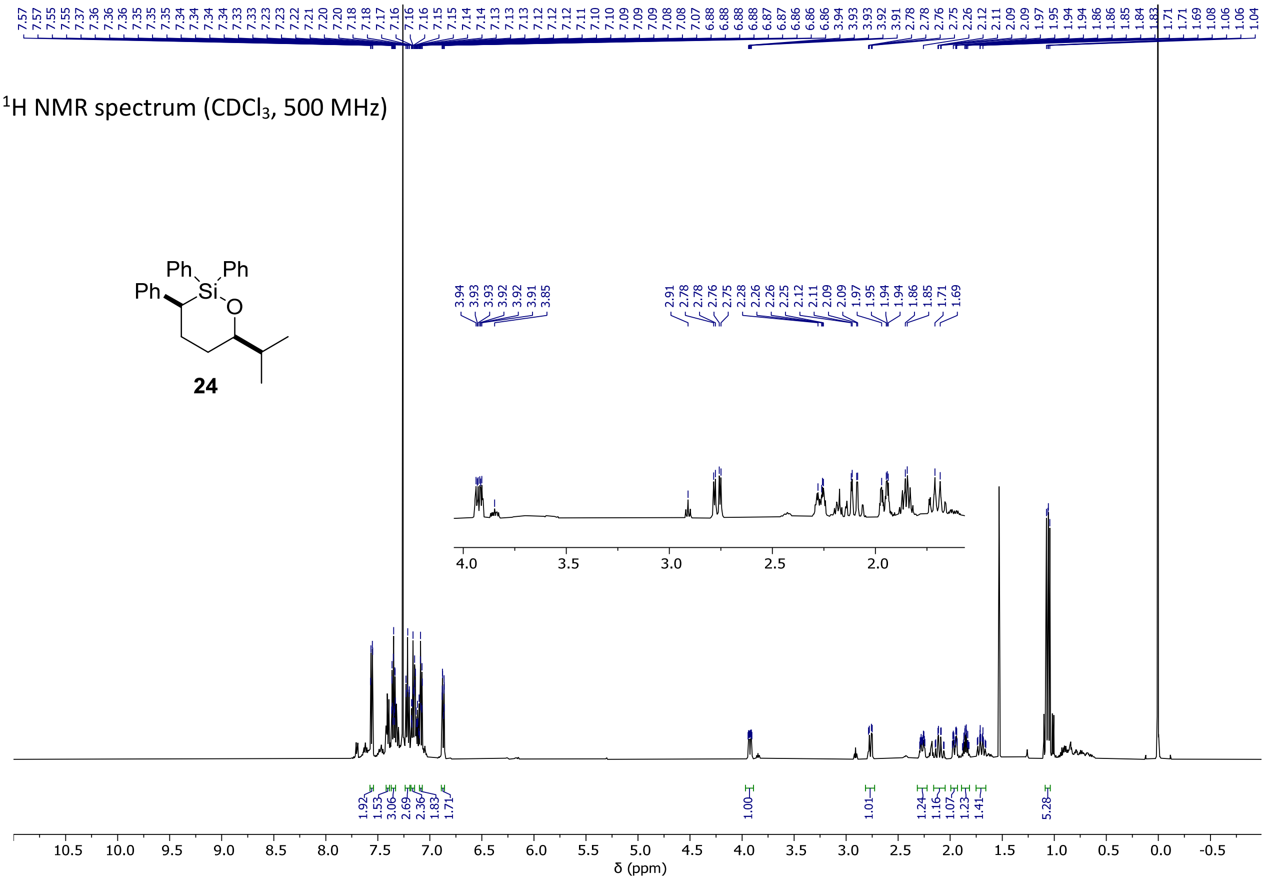

$^{13}C\{^{1}H\}$ NMR spectrum ($CDCl_3$, 126 MHz)

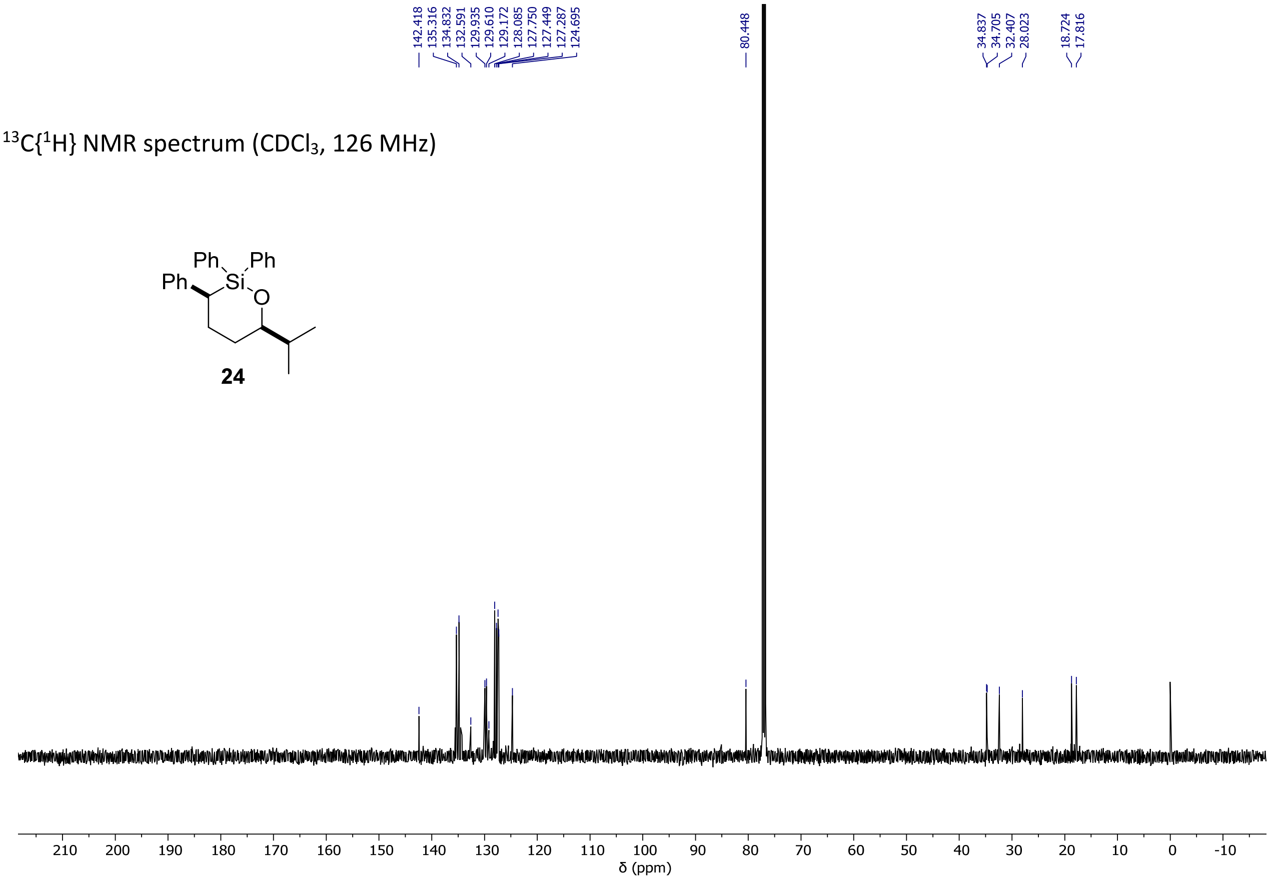

^{1}H NMR spectrum ($CDCl_3$, 500 MHz)

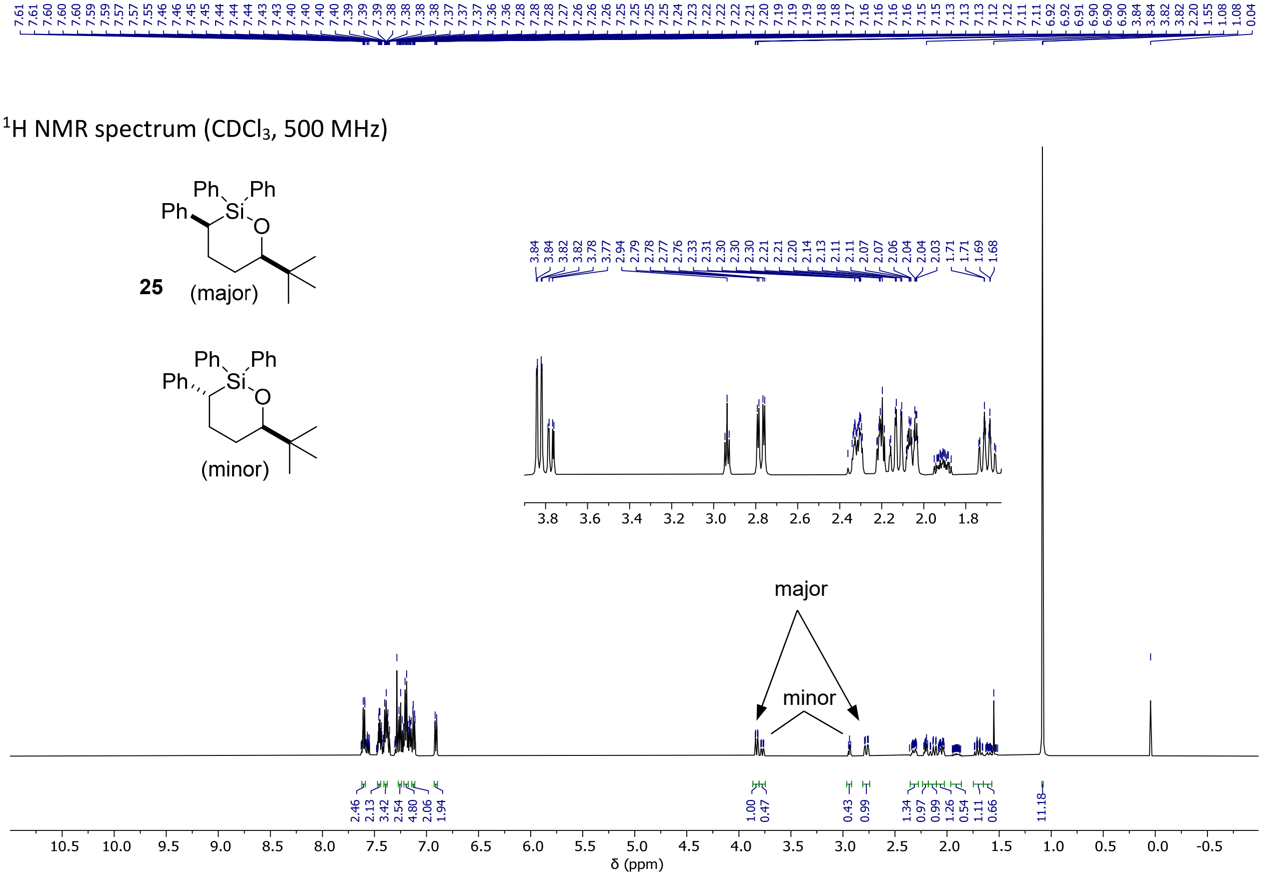

$^{13}C\{^{1}H\}$ NMR spectrum ($CDCl_3$, 126 MHz)

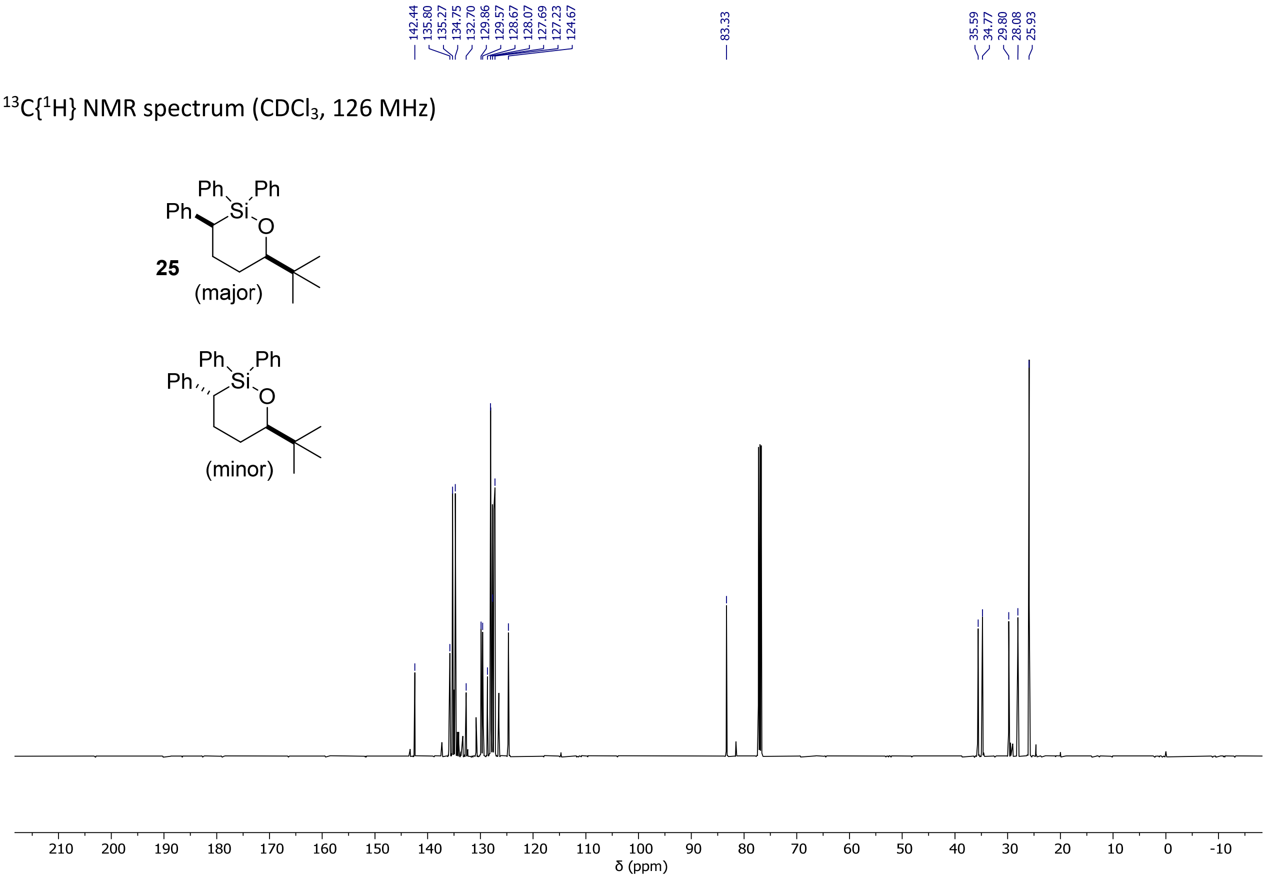

^{1}H NMR spectrum ($CDCl_3$, 500 MHz)

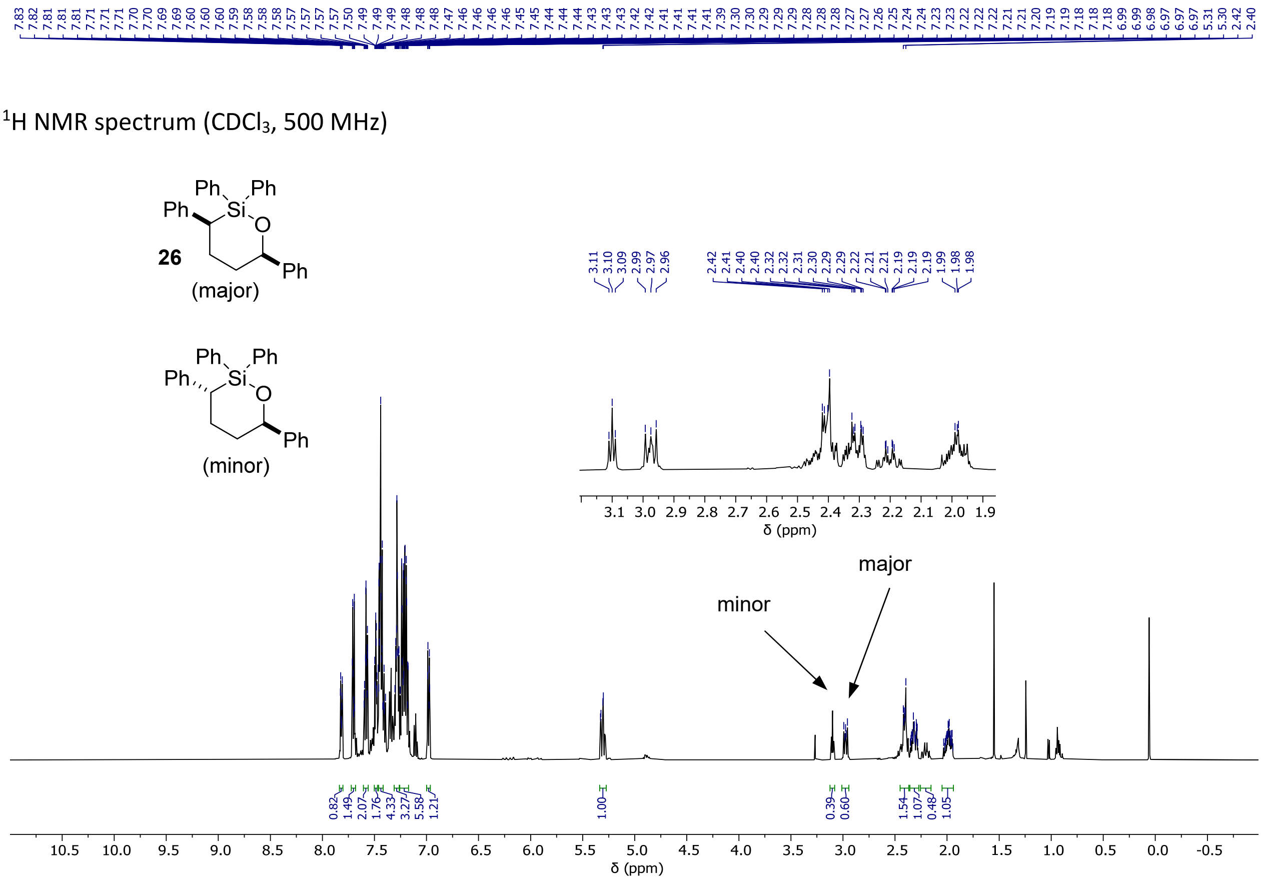

^{13}C{^{1}H} NMR spectrum ($CDCl_3$, 126 MHz)

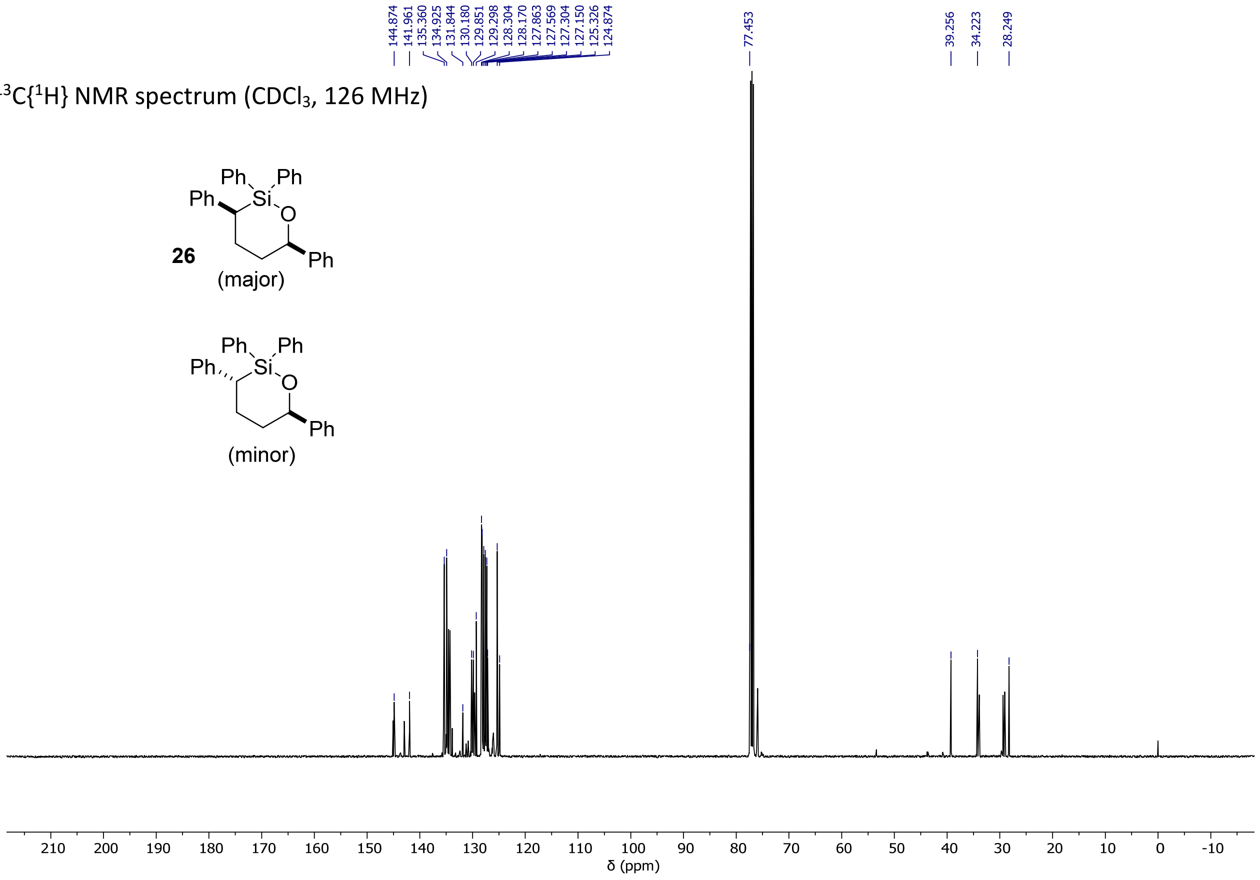

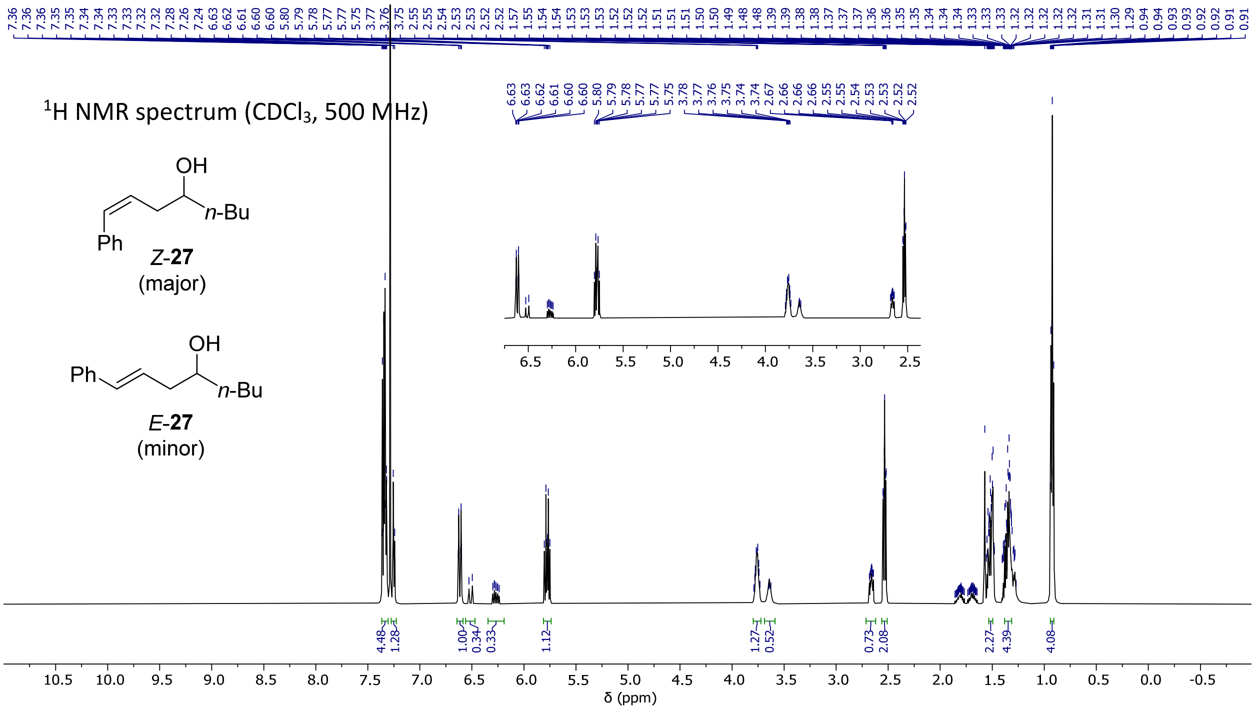
1H NMR spectrum (CDCl3, 500 MHz)
OH
n-Bu
Ph
Z-27
(major)
OH
Ph
n-Bu
E-27
(minor)
δ (ppm)

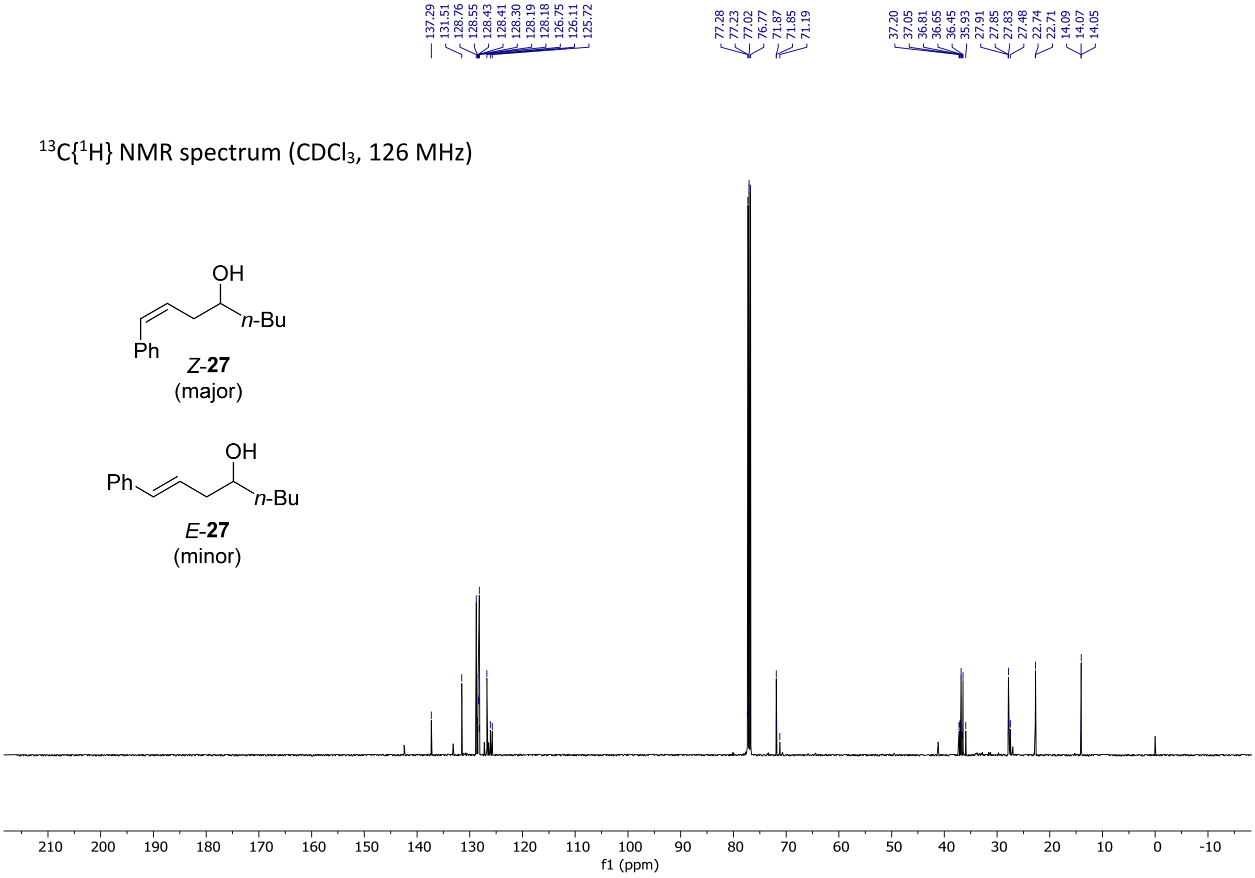
13C{1H} NMR spectrum (CDCl3, 126 MHz)
OH
n-Bu
Ph
Z-27
(major)
OH
Ph
n-Bu
E-27
(minor)
f1 (ppm)

^{1}H NMR spectrum ($CDCl_3$, 500 MHz)

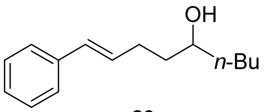

29

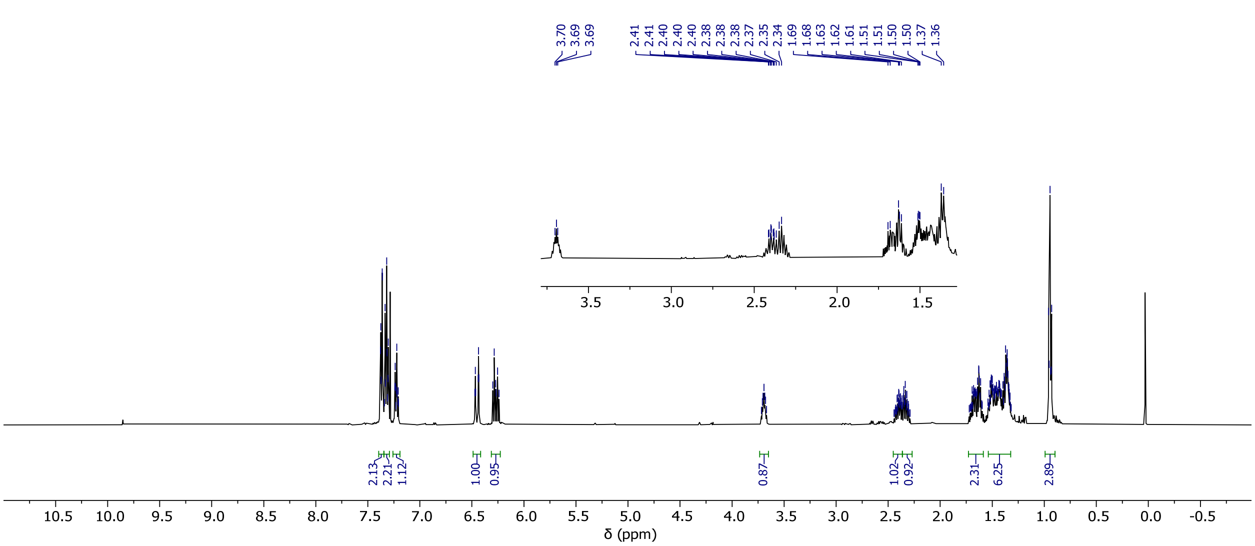

$^{13}C\{^{1}H\}$ NMR spectrum ($CDCl_3$, 126 MHz)

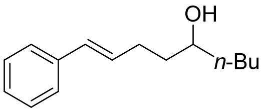

29

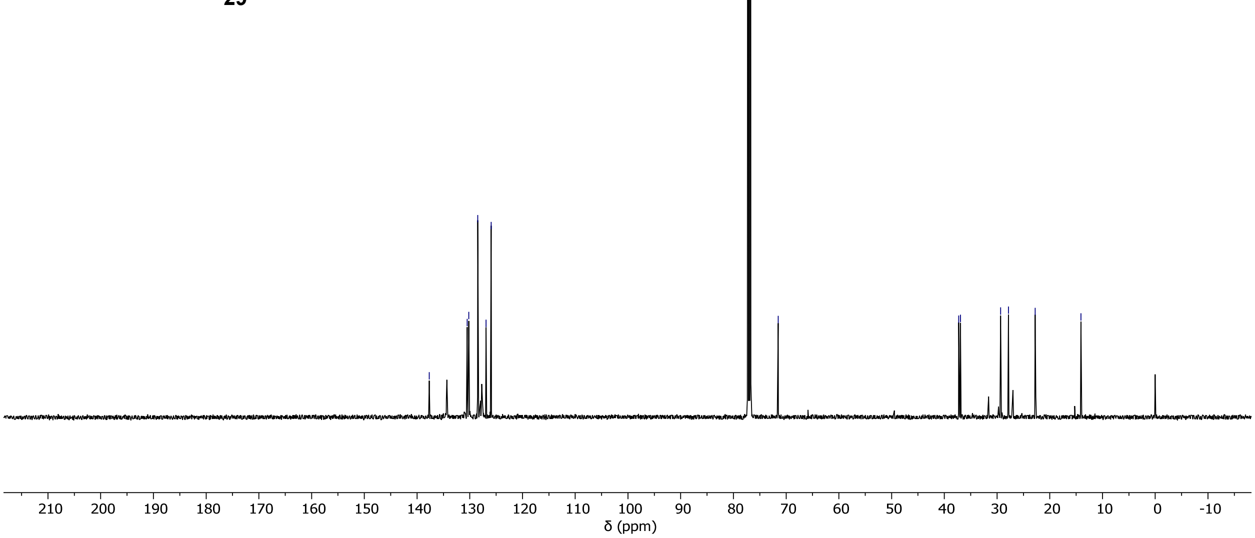

^{1}H NMR spectrum ($CDCl_3$, 500 MHz)

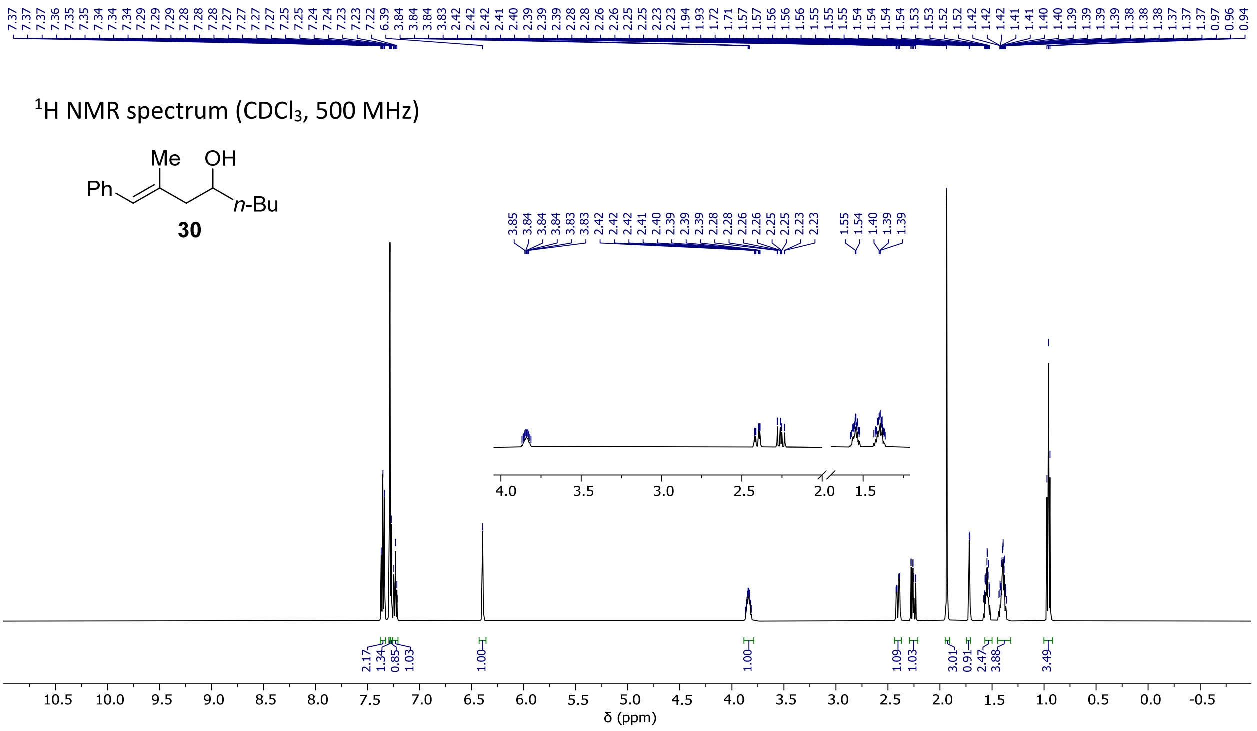

$^{13}C\{^{1}H\}$ NMR spectrum ($CDCl_3$, 126 MHz)

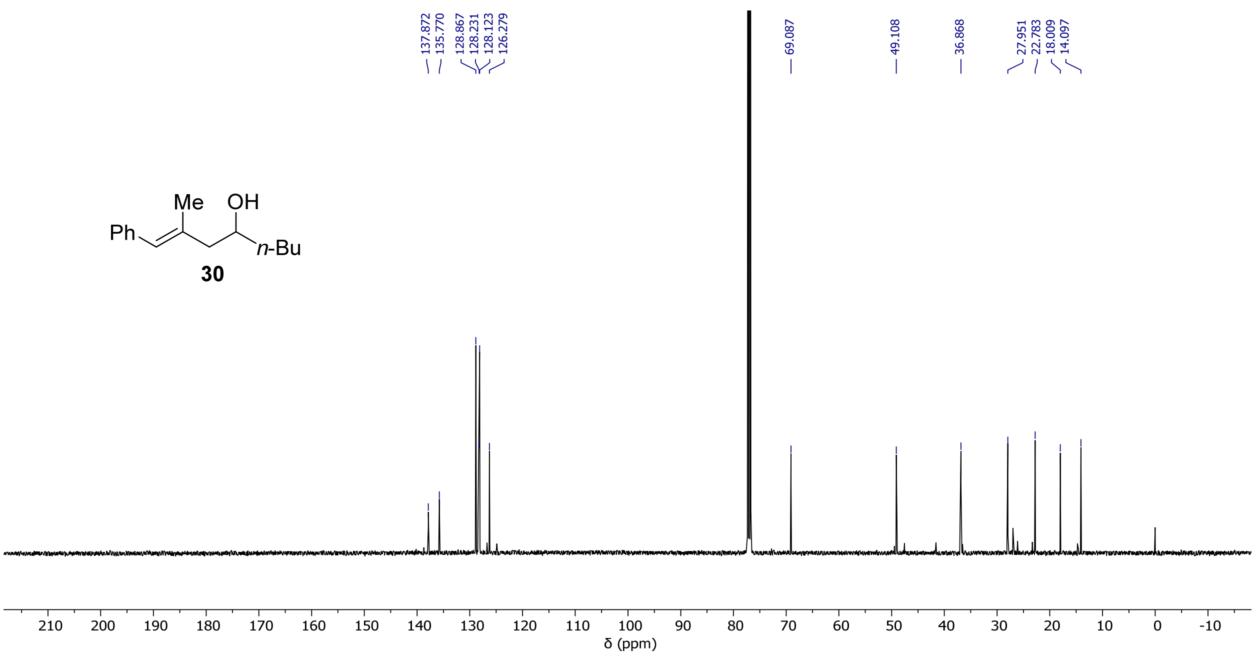

^{1}H NMR spectrum ($CDCl_3$, 500 MHz)

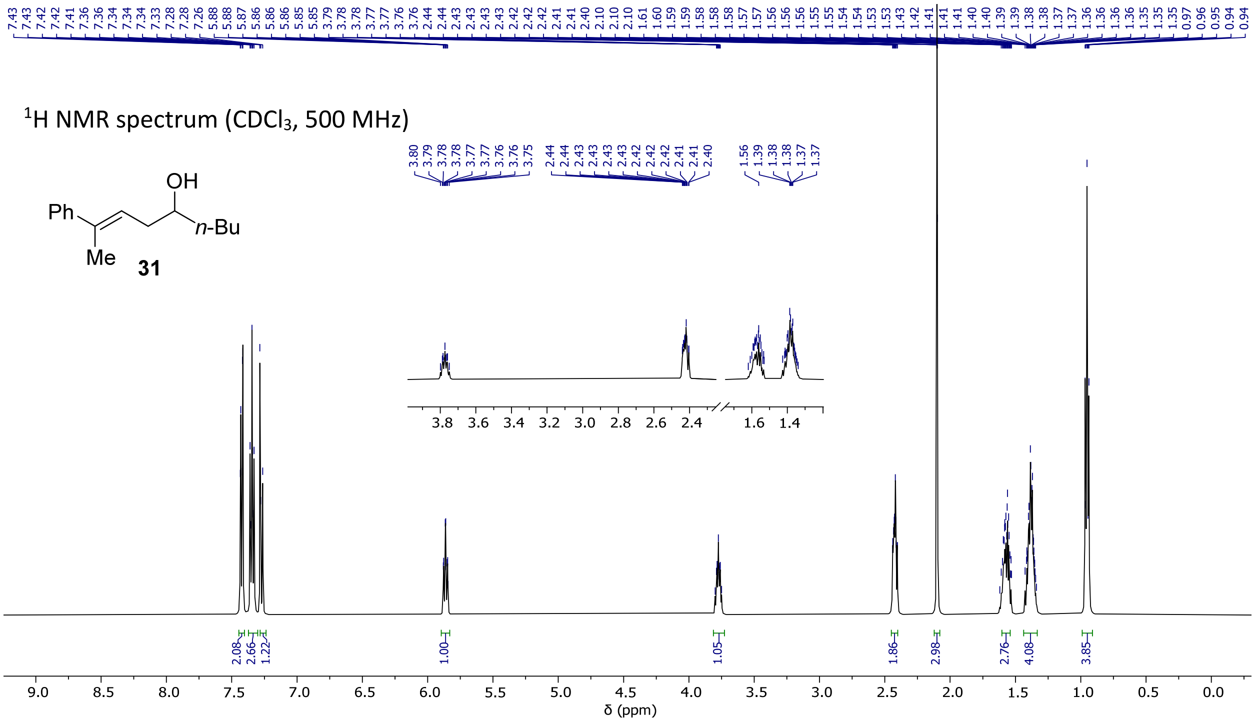

^{13}C{^{1}H} NMR spectrum ($CDCl_3$, 126 MHz)

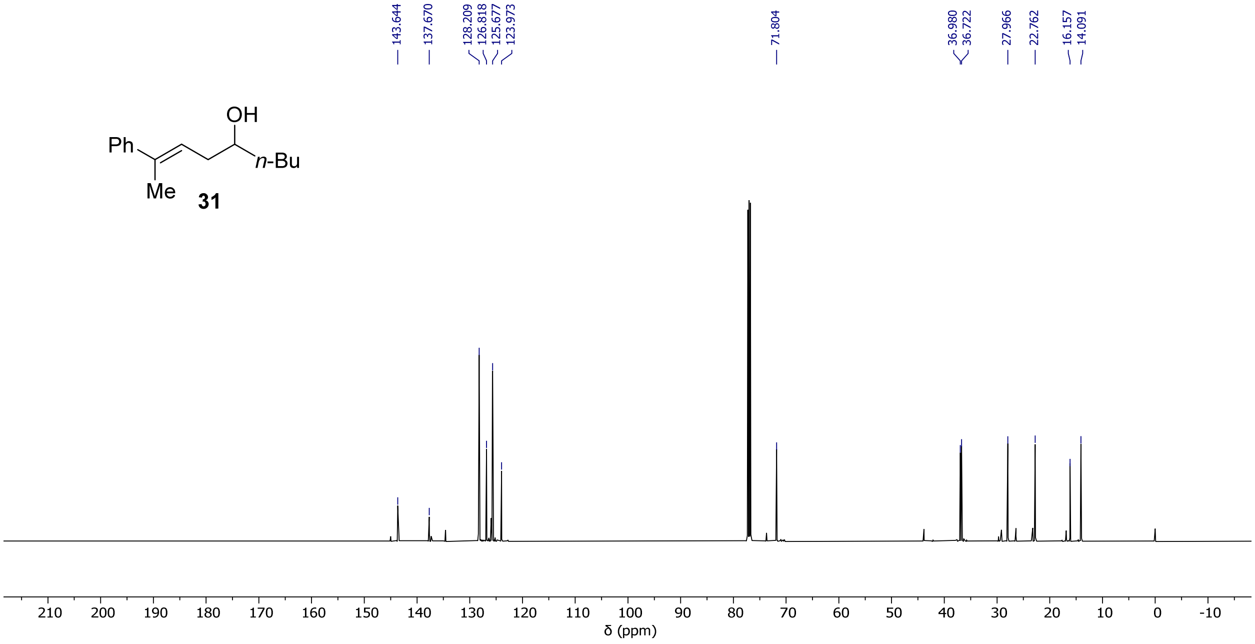

^{1}H NMR spectrum ($CDCl_3$, 500 MHz)

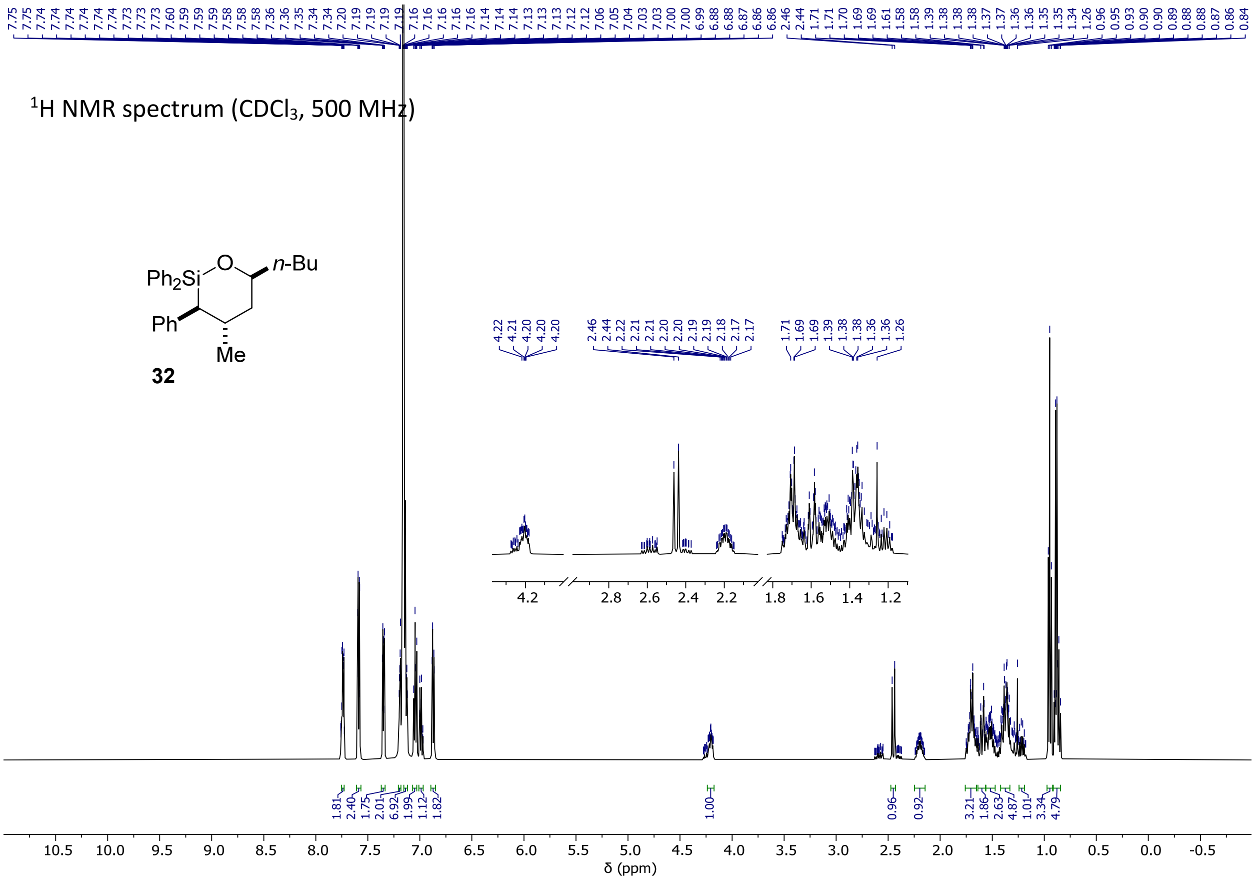

$^{13}C\{^{1}H\}$ NMR spectrum ($CDCl_3$, 126 MHz)

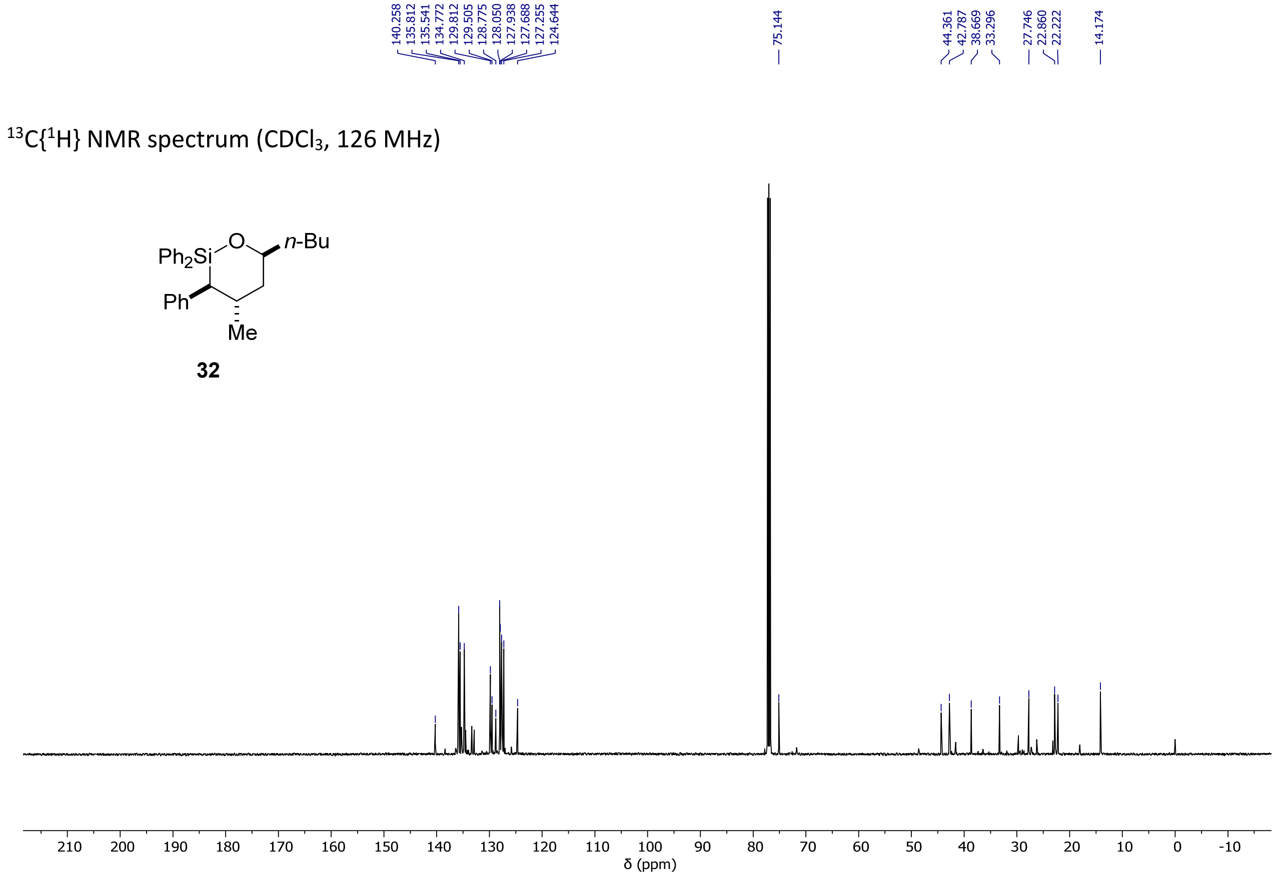

^{1}H NMR spectrum ($CDCl_3$, 500 MHz)

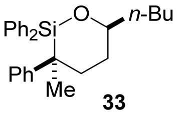

33

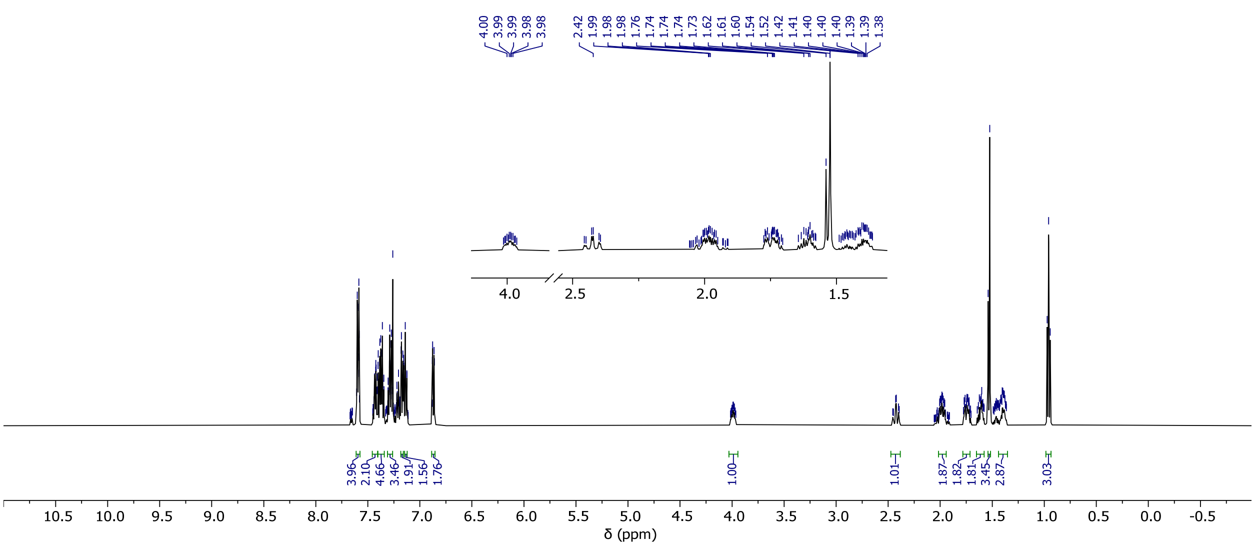

^{13}C{^{1}H} NMR spectrum ($CDCl_3$, 126 MHz)

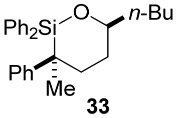

33

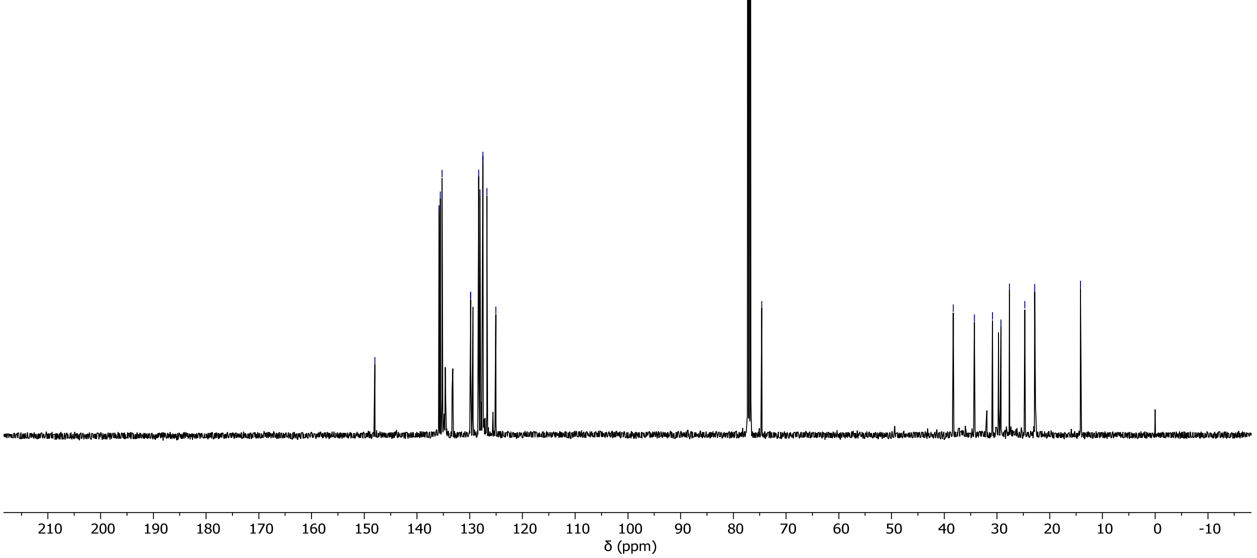

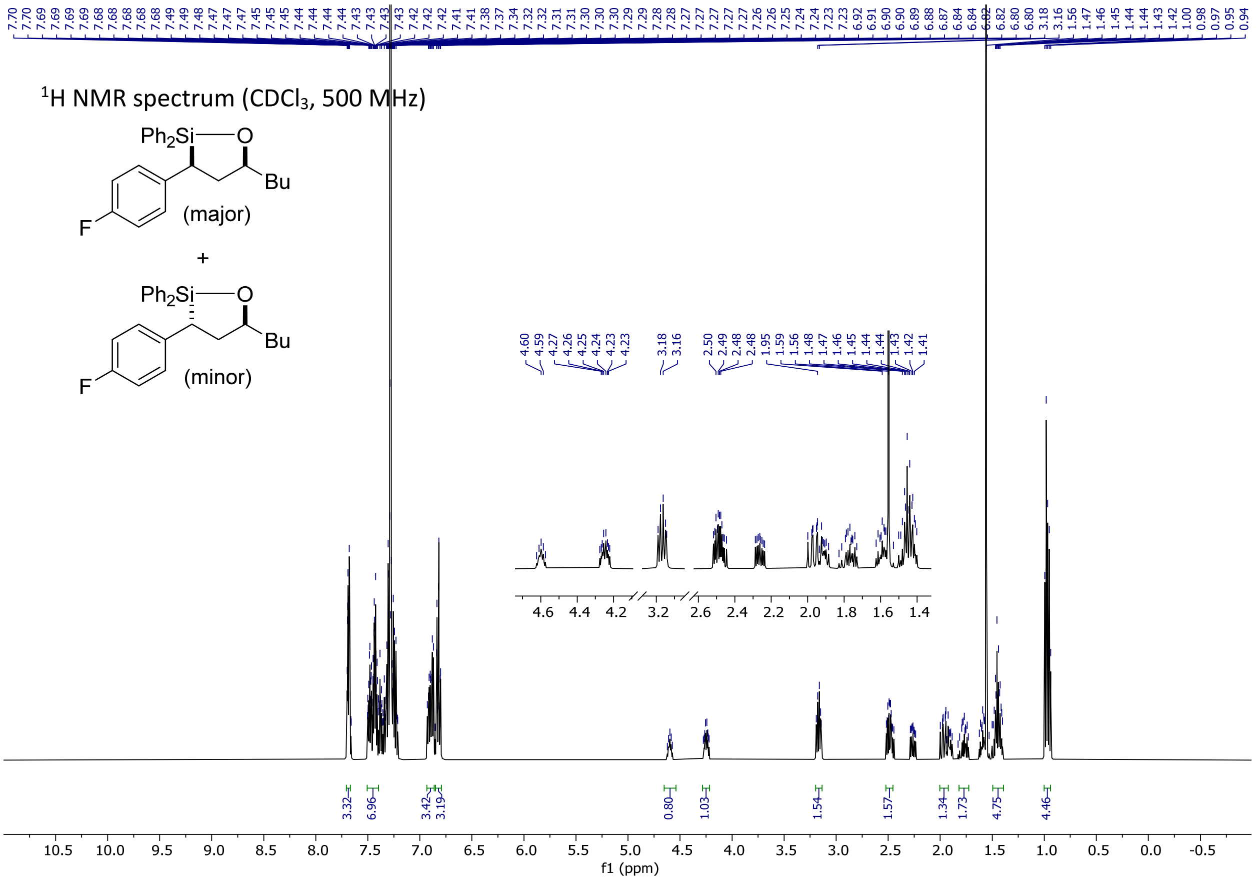

^{1}H NMR spectrum (CDCl$_3$, 500 MHz)
Ph$_2$Si
O
Bu
F
(major)
+
Ph$_2$Si
O
Bu
F
(minor)
f1 (ppm)

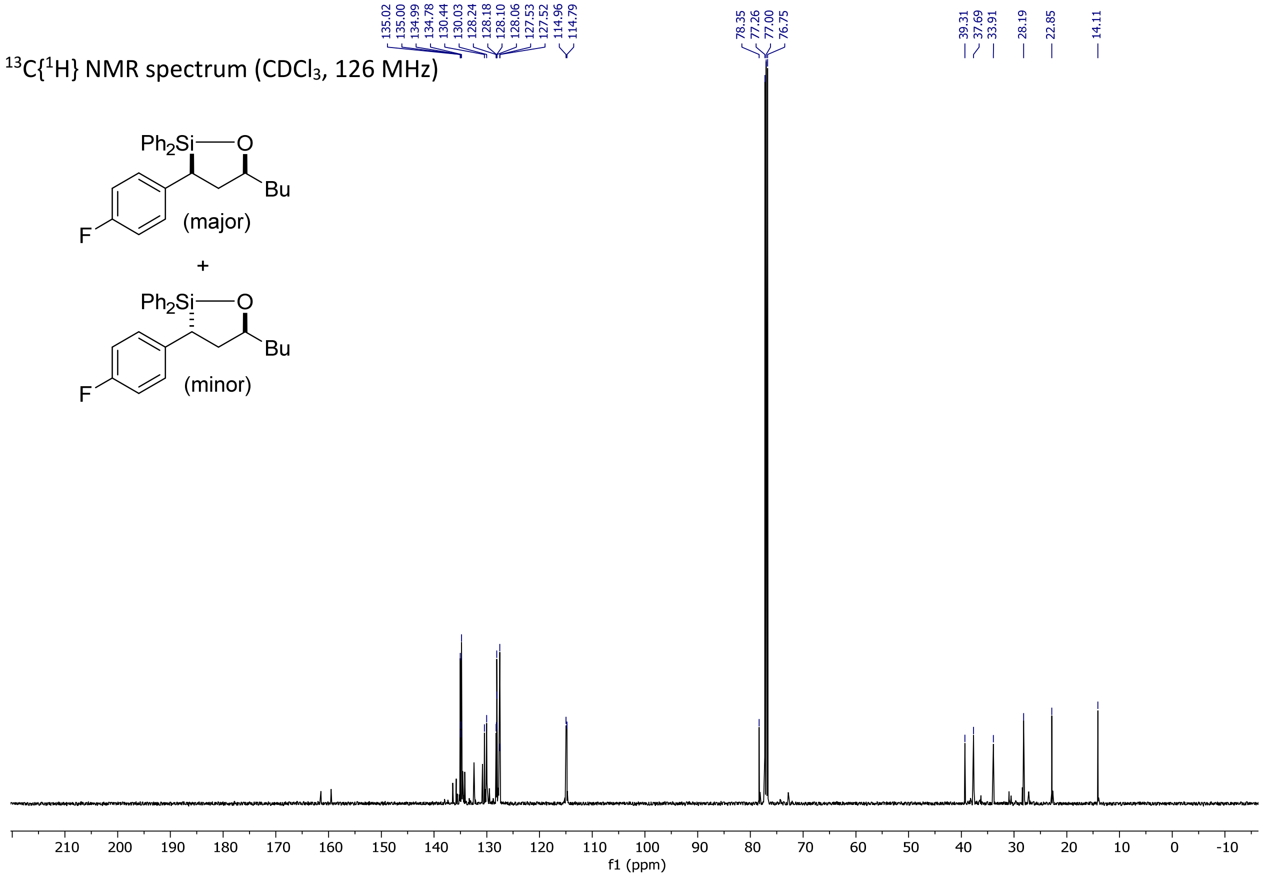

^{13}C{^{1}H} NMR spectrum (CDCl$_3$, 126 MHz)
135.02
135.00
134.99
134.78
130.44
130.03
128.24
128.18
128.10
128.06
127.53
127.52
114.96
114.79
78.35
77.26
77.00
76.75
39.31
37.69
33.91
28.19
22.85
14.11
Ph$_2$Si
O
Bu
F
(major)
+
Ph$_2$Si
O
Bu
F
(minor)
f1 (ppm)

^{1}H NMR spectrum ($CDCl_3$, 500 MHz)

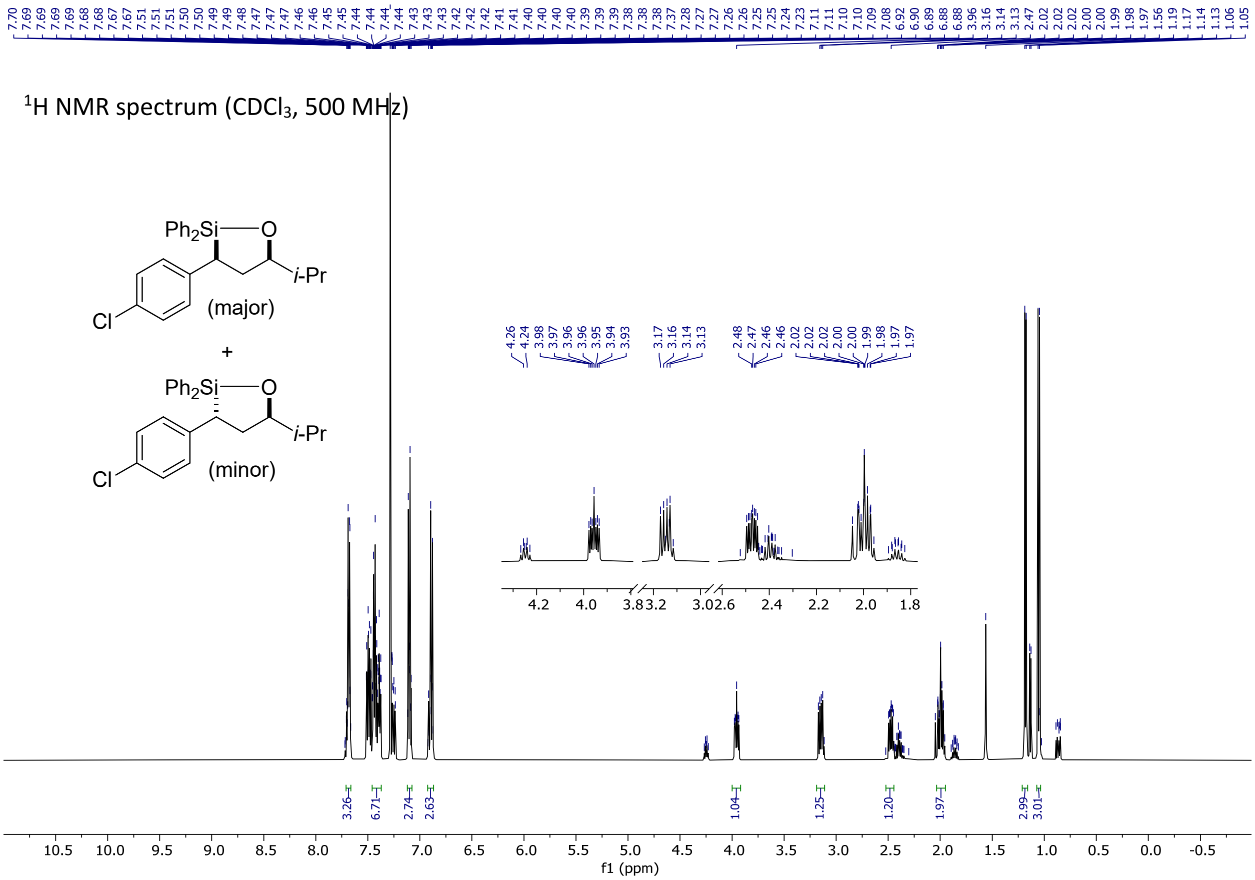

^{13}C{^{1}H} NMR spectrum ($CDCl_3$, 126 MHz)

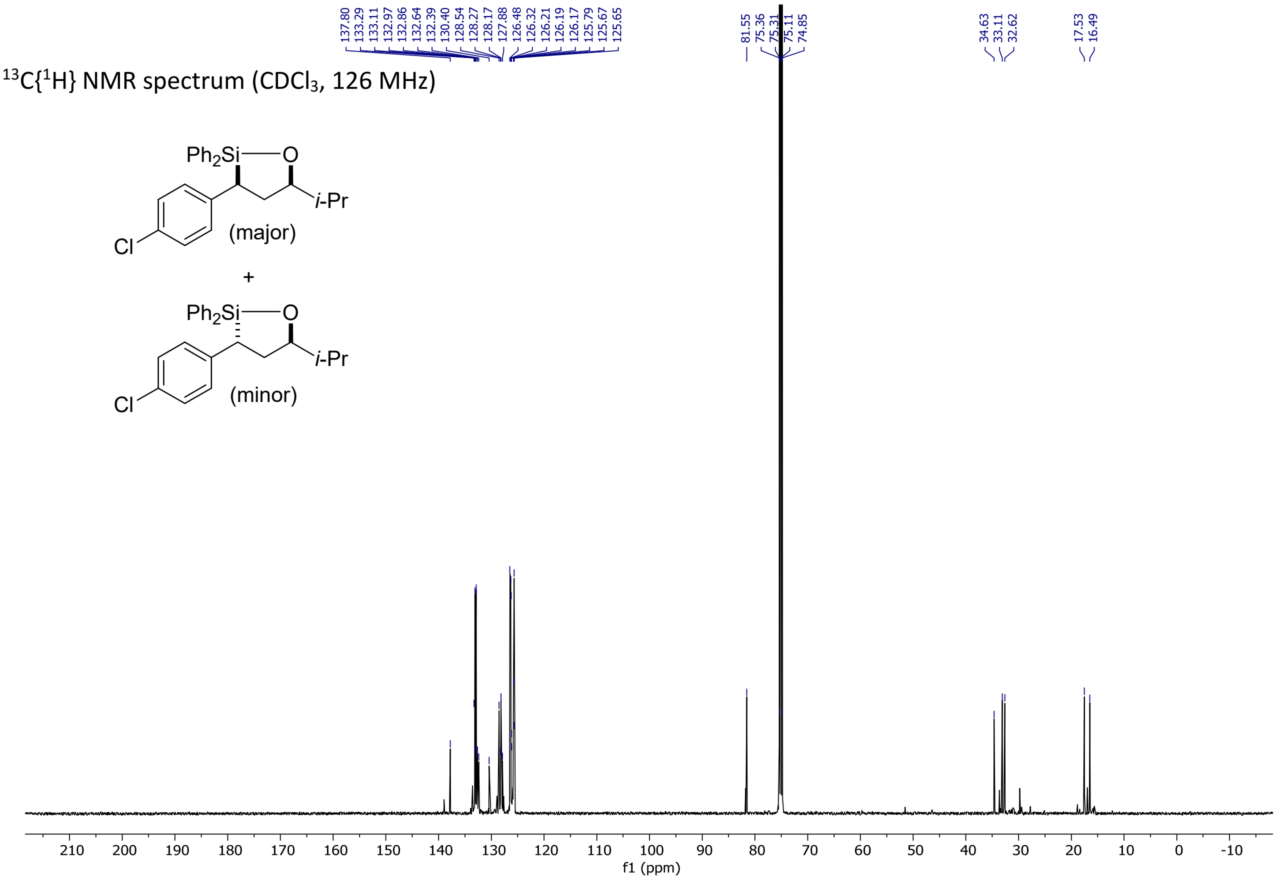

^{1}H NMR spectrum ($CDCl_3$, 500 MHz)

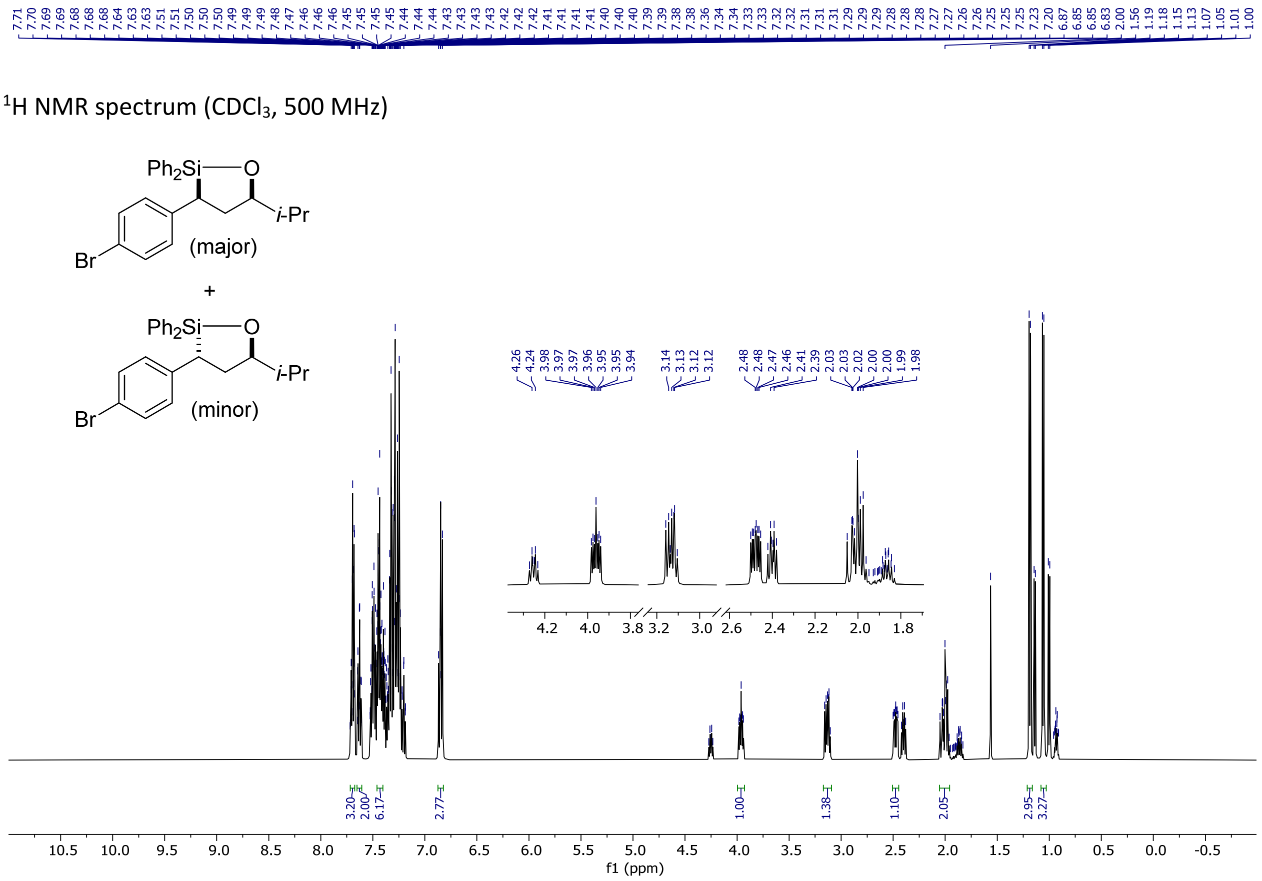

$^{13}C\{^{1}H\}$ NMR spectrum ($CDCl_3$, 126 MHz)

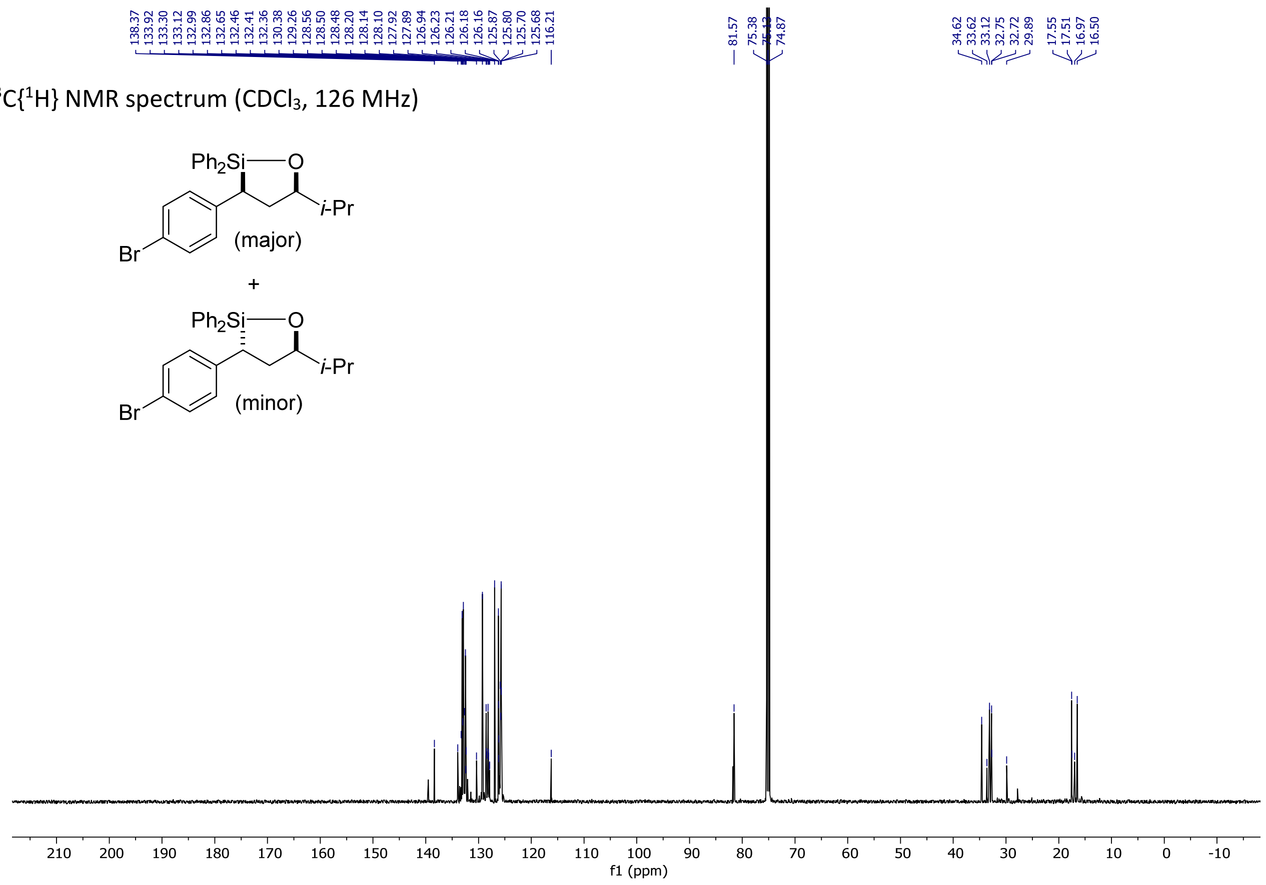

^{1}H NMR spectrum ($CDCl_3$, 500 MHz)

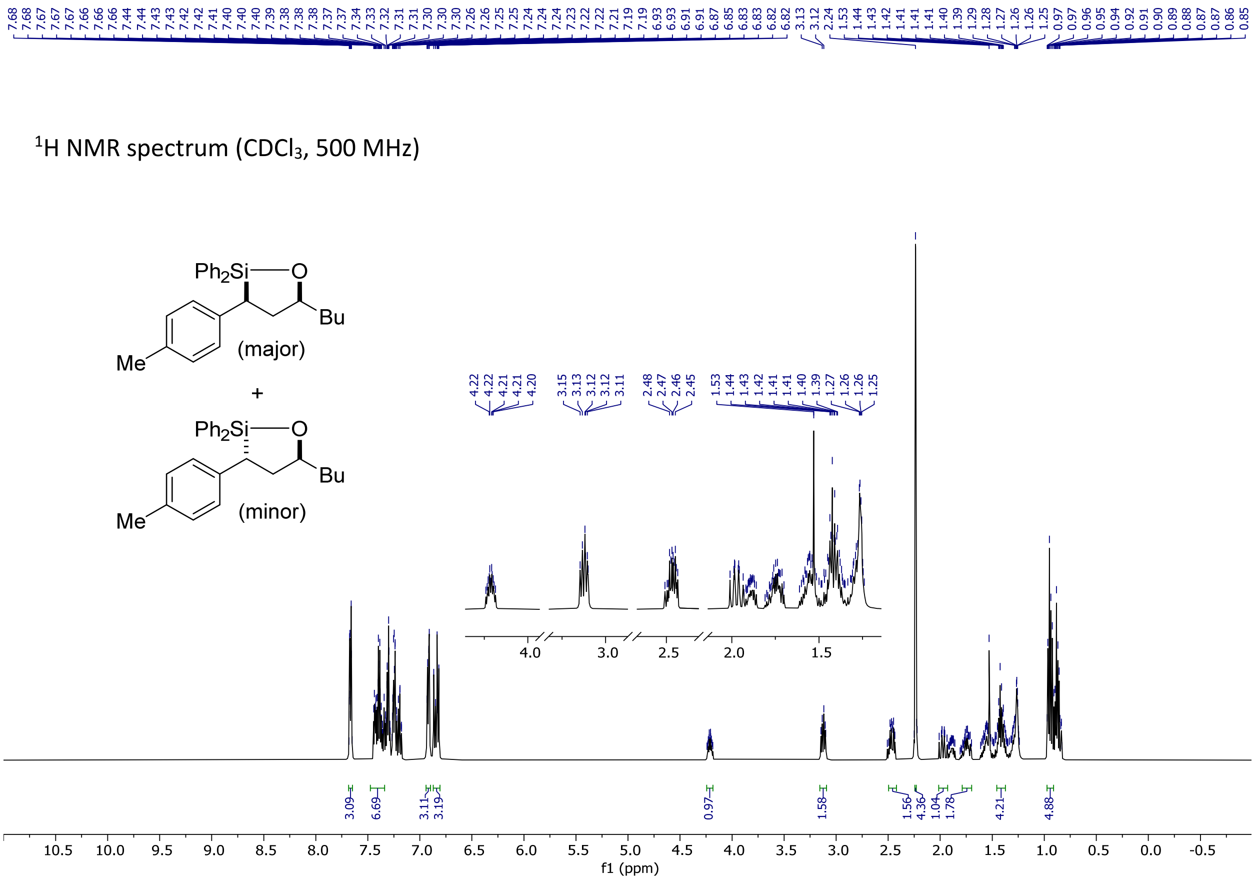

$^{13}C\{^{1}H\}$ NMR spectrum ($CDCl_3$, 126 MHz)

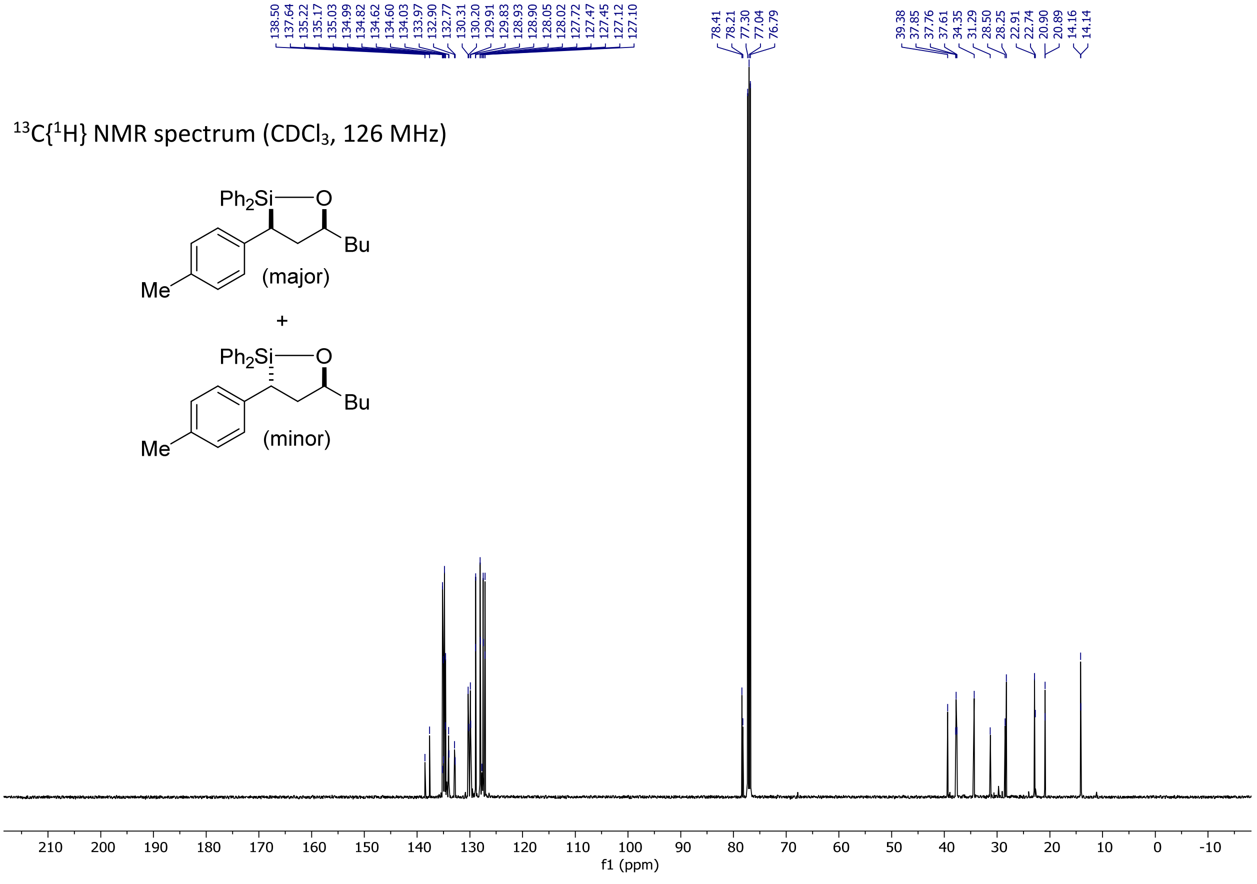

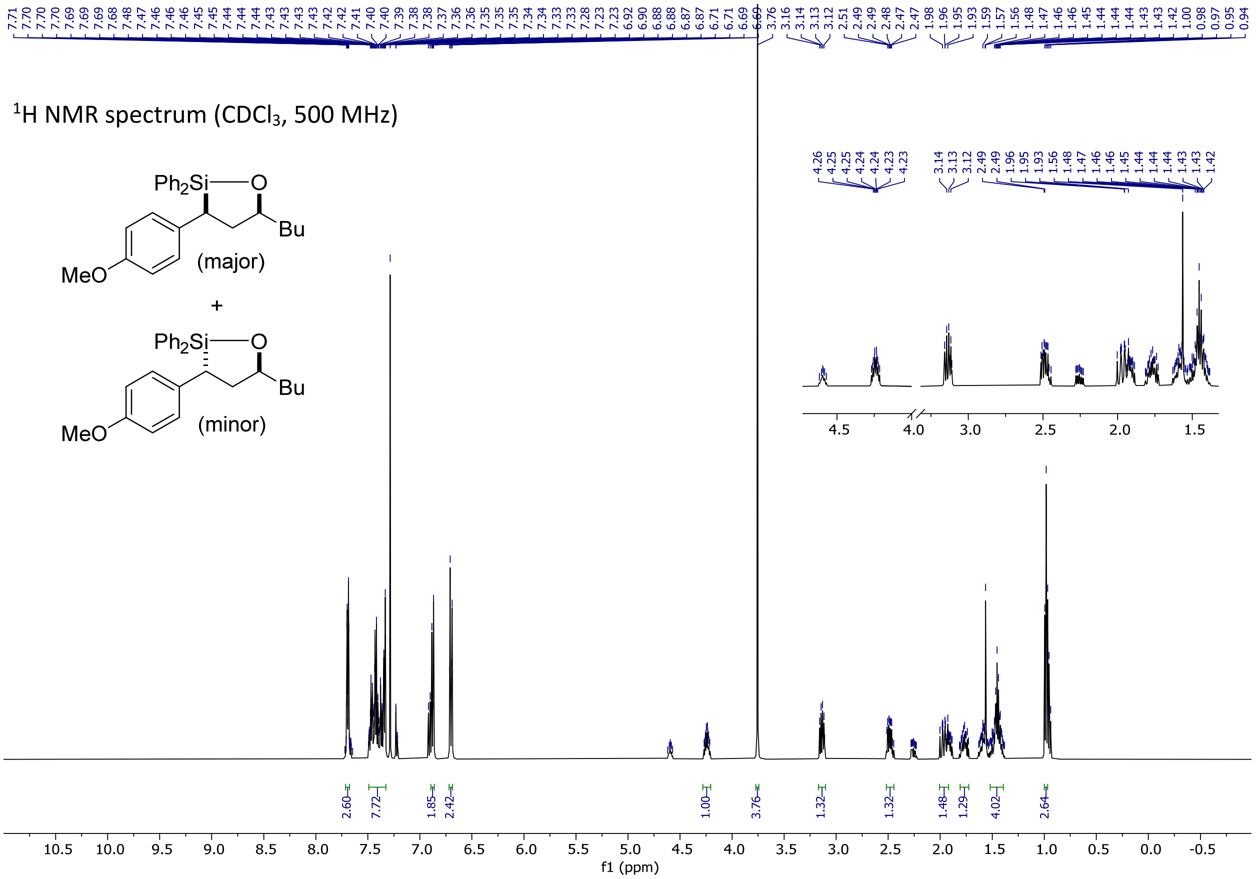
7.71 7.70 7.70 7.69 7.69 7.69 7.68 7.48 7.47 7.46 7.46 7.45 7.45 7.44 7.44 7.44 7.43 7.43 7.43 7.43 7.42 7.42 7.41 7.40 7.40 7.39 7.38 7.38 7.37 7.36 7.36 7.35 7.35 7.34 7.34 7.33 7.33 7.28 7.23 7.23 6.92 6.90 6.88 6.87 6.71 6.71 6.69 6.69 3.76 3.16 3.14 3.13 3.12 2.51 2.49 2.49 2.48 2.47 2.47 1.98 1.96 1.95 1.93 1.59 1.57 1.56 1.48 1.47 1.46 1.46 1.45 1.44 1.44 1.44 1.43 1.43 1.42 1.00 0.98 0.97 0.95 0.94
^{1}H NMR spectrum ($CDCl_3$, 500 MHz)
Ph_2Si
O
Bu
MeO
(major)
+
Ph_2Si
O
Bu
MeO
(minor)
4.26 4.25 4.25 4.24 4.24 4.23 4.23 3.14 3.13 3.12 2.49 2.49 1.96 1.95 1.93 1.56 1.48 1.47 1.46 1.46 1.45 1.44 1.44 1.44 1.43 1.43 1.42
4.5 4.0 3.0 2.5 2.0 1.5
2.60 7.72 1.85 2.42 1.00 3.76 1.32 1.32 1.48 1.29 4.02 2.64
10.5 10.0 9.5 9.0 8.5 8.0 7.5 7.0 6.5 6.0 5.5 5.0 4.5 4.0 3.5 3.0 2.5 2.0 1.5 1.0 0.5 0.0 -0.5
f1 (ppm)

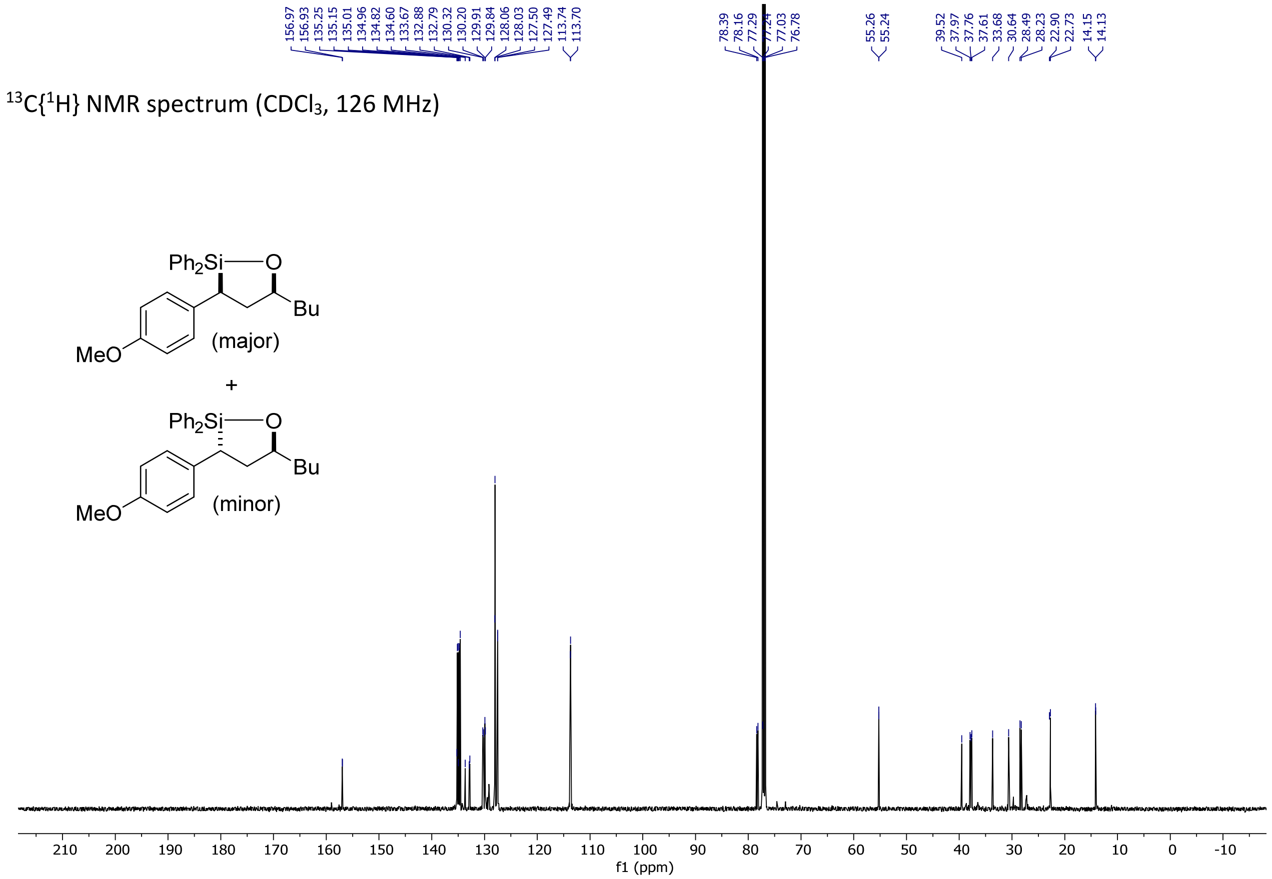
156.97 156.93 135.25 135.15 135.01 134.96 134.82 134.60 133.67 132.88 132.79 130.32 130.20 129.91 129.84 128.06 128.03 127.50 127.49 113.74 113.70
78.39 78.16 77.29 77.24 77.03 76.78
55.26 55.24
39.52 37.97 37.76 37.61 33.68 30.64 28.49 28.23 22.90 22.73 14.15 14.13
^{13}C{^{1}H} NMR spectrum ($CDCl_3$, 126 MHz)
Ph_2Si
O
Bu
MeO
(major)
+
Ph_2Si
O
Bu
MeO
(minor)
210 200 190 180 170 160 150 140 130 120 110 100 90 80 70 60 50 40 30 20 10 0 -10
f1 (ppm)

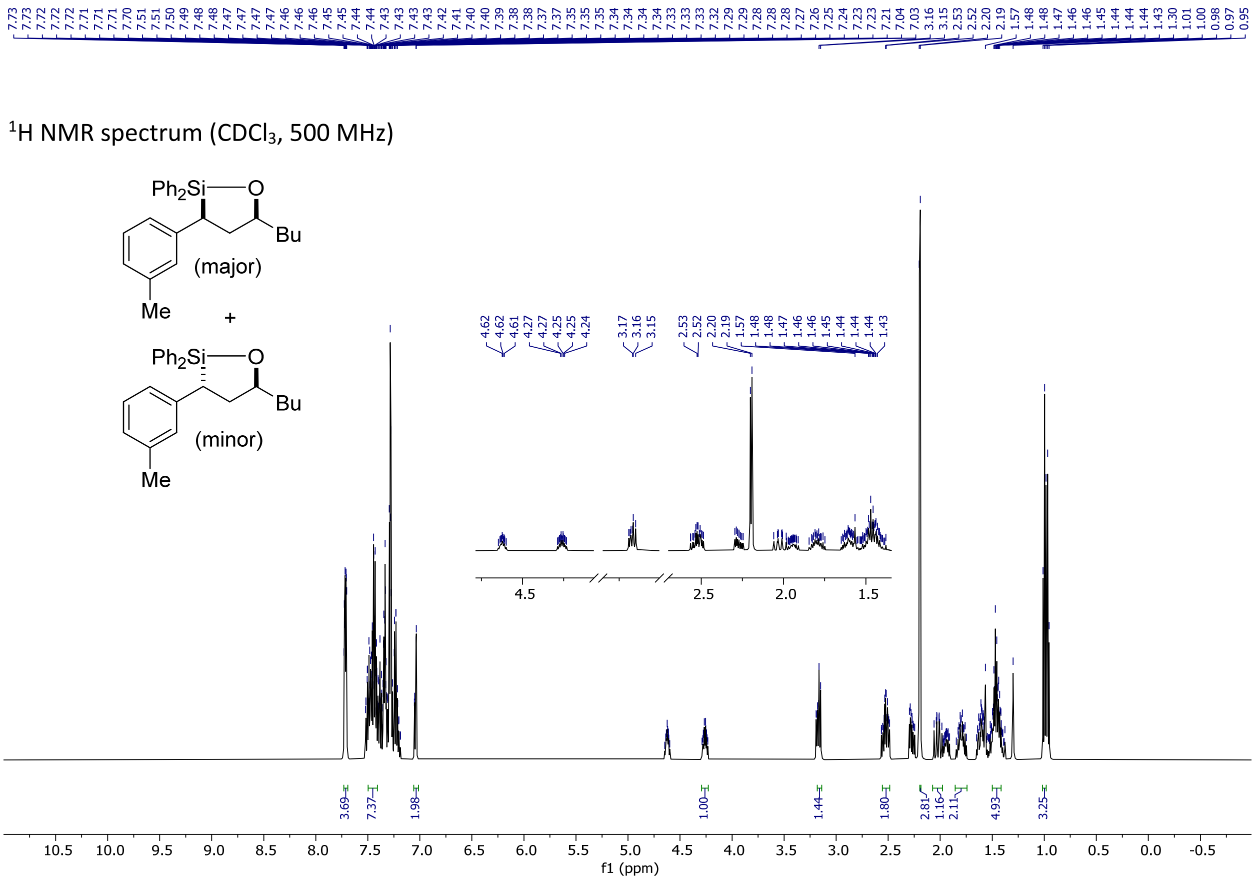

^{1}H NMR spectrum (CDCl$_3$, 500 MHz)
Ph$_2$Si
O
Bu
(major)
Me
+
Ph$_2$Si
O
Bu
(minor)
Me
f1 (ppm)

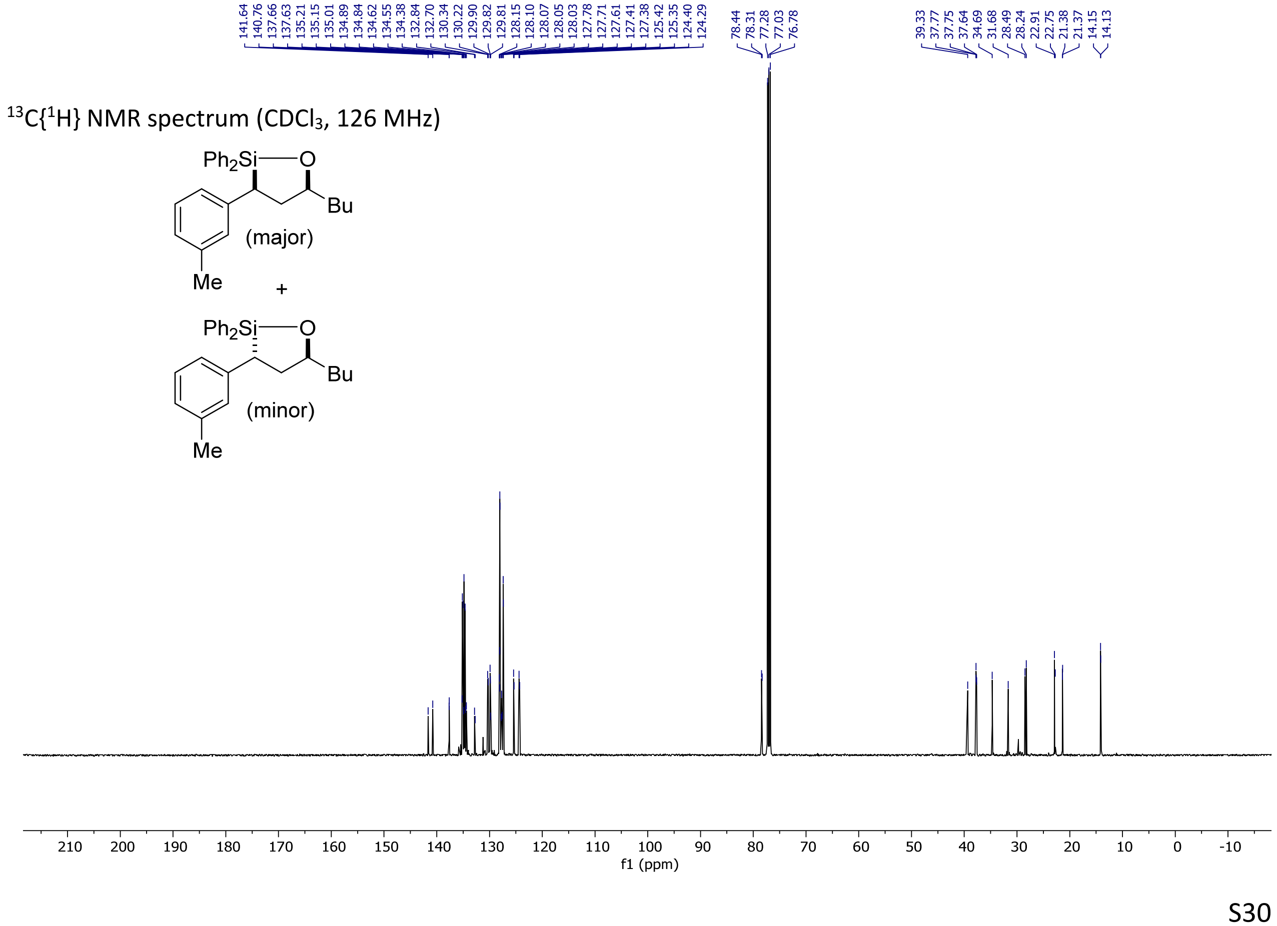

^{13}C{^{1}H} NMR spectrum (CDCl$_3$, 126 MHz)
Ph$_2$Si
O
Bu
(major)
Me
+
Ph$_2$Si
O
Bu
(minor)
Me
f1 (ppm)

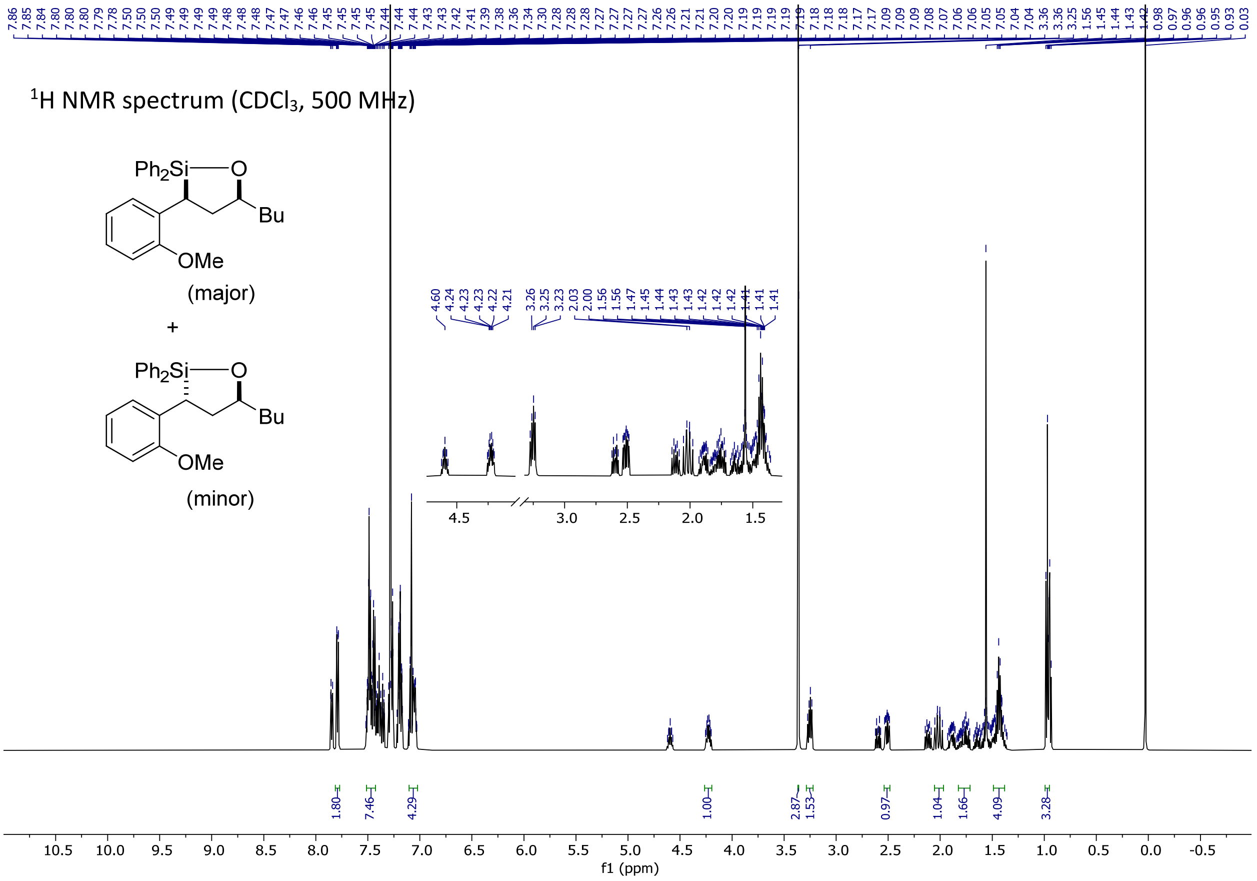
1H NMR spectrum (CDCl3, 500 MHz)
Ph2Si
O
Bu
OMe
(major)
+
Ph2Si
O
Bu
OMe
(minor)
f1 (ppm)

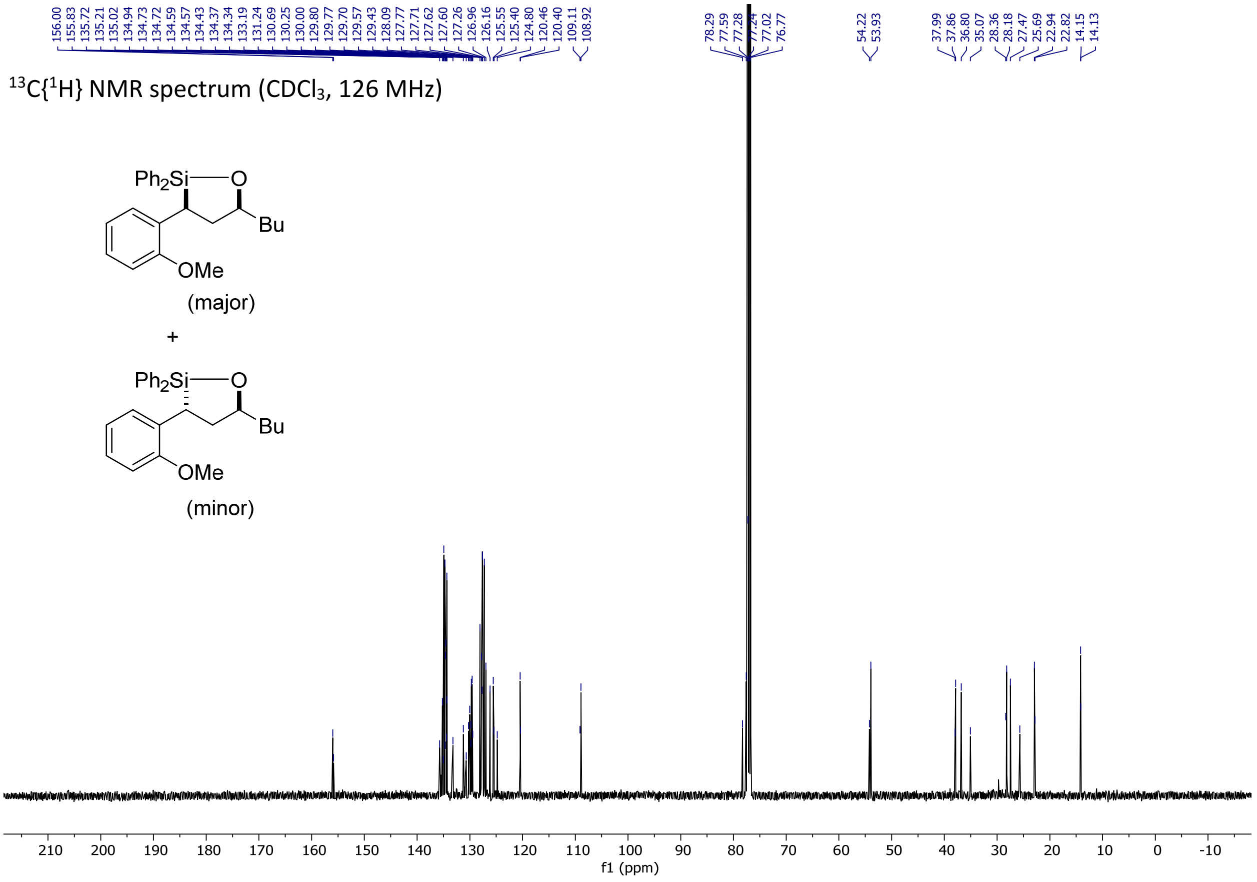
13C{1H} NMR spectrum (CDCl3, 126 MHz)
Ph2Si
O
Bu
OMe
(major)
+
Ph2Si
O
Bu
OMe
(minor)
f1 (ppm)

Additive Screening Results for Hydrosilylation of 24.

Experiments were performed by preparing a solution of **34** (250 mg) plus the indicated additive (~1.0 equiv.) and 1-octene (~1.0 equiv.) as an inert internal standard in toluene-d8. A ^{1}H NMR spectrum of the mixture was then obtained and signals corresponding to **34**, the additive, and 1-octene were integrated to determine a starting molar ratio (Table S1, left columns, time = 0). TBAT (0.2 equiv.) was then added and the mixture was heated to 80 °C for 2 h. The resulting product mixture was analyzed directly by ^{1}H NMR, with signals corresponding to **2**, 1-octene, and the additive integrated to determine the yield of **2** from **34** and the additive recovery percentage (right columns, time = 2 hr).

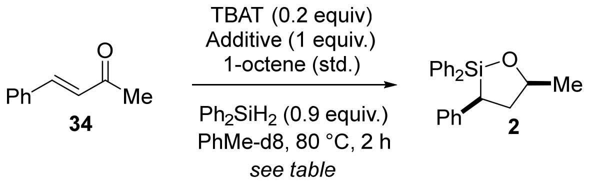

**Time = 0**		
**Additive (∫)**	**∫ 34**	**∫ 1-octene**
--	1.34	1.0
ethyl acetate (0.81)	1.06	1.0
pyridine (0.82)	0.71	1.0
acetonitrile (1.24)	1.16	1.0
nitromethane (1.03)	0.92	1.0

**Time = 2 hr**		
**∫ 2 (Yield %)**	**∫ 1-octene**	**∫ Additive (%Recovery)**
0.48 (36)	1.0	--
0.35 (33)	1.0	0.58 (72)
0.25 (35)	1.0	0.62 (76)
0.31 (27)	1.0	0.87 (70)
--	1.0	--

Crystallographic data for the structure of compound 25.

A colorless block, measuring 0.19 x 0.13 x 0.05 mm^3 was mounted on a loop with oil. Data was collected at -173ºC on a Bruker APEX II single crystal X-ray diffractometer, Mo-radiation, equipped with a Miracol X-ray optical collimator.

Crystal-to-detector distance was 40 mm and exposure time was 100 seconds per frame for all sets. The scan width was 0.5º. Data collection was xx.x% complete to 25º in ϑ. A total of xxx reflections were collected covering the indices, -12<=h<=12, -15<=k<=15, -15<=l<=15. 34155 reflections were symmetry independent and the R_{int} = 0.0330 indicated that the data was brilliant. Indexing and unit cell refinement indicated a triclinic lattice. The space group was found to be $P\bar{1}$ (No. 2).

The data was integrated and scaled using SAINT, SADABS within the APEX2 software package by Bruker.[1]

Solution by direct methods (SHELXT[2] or SIR97[3]) produced a complete heavy atom phasing model consistent with the proposed structure. The structure was completed by difference Fourier synthesis with SHELXL.[4] Scattering factors are from Waasmair and Kirfel[5]. Hydrogen atoms were placed in geometrically idealised positions and constrained to ride on their parent atoms with C---H distances in the range 0.95-1.00 Angstrom. Isotropic thermal parameters U_{eq} were fixed such that they were 1.2U_{eq} of their parent atom Ueq for CH's and 1.5U_{eq} of their parent atom U_{eq} in case of methyl groups. All non-hydrogen atoms were refined anisotropically by full-matrix least-squares.

The centro-symmetric structure does not allow absolute determination of the handednesses of chiral centers, however chiral centers C1 and C4 have same sense of handednes.

There are no hydrogen bonds in this structure.

The structure is of high quality and ready for publication. Table S1 summarizes the data collection details. Figure 1 shows an ORTEP[6] of the asymmetric unit.

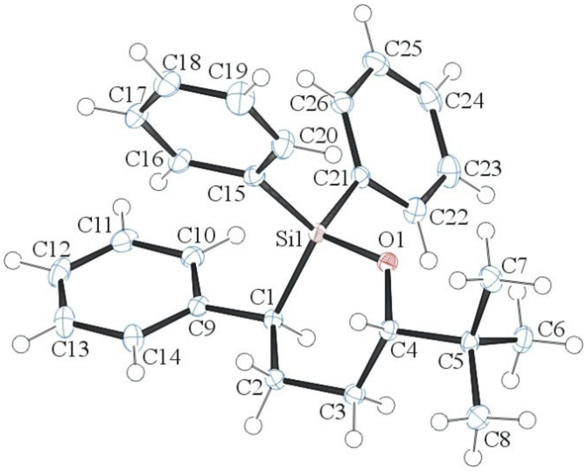

Figure S1. ORTEP of **25** with thermal ellipsoids at the 50% probability level.

Table S1. Crystallographic data for the structure of compound **25**.

Empirical formula	C26 H30 O Ss.i	
Formula weight	386.59	
Temperature	100(2) K	
Wavelength	0.71073 Å	
Crystal system	Triclinic	
Space group	P -1	
Unit cell dimensions	a = 9.0039(5) Å	a= 64.543(3)°.
	b = 11.4769(6) Å	b= 77.586(3)°.
	c = 11.8265(6) Å	g = 81.771(3)°.
Volume	1075.87(10) $Å^3$	
Z	2	
Density (calculated)	1.193 Mg/m^3	
Absorption coefficient	0.123 mm^{-1}	
F(000)	416	
Crystal size	0.190 x 0.130 x 0.050 mm^3	
Theta range for data collection	1.936 to 28.547°.	
Index ranges	-12<=h<=12, -15<=k<=15, -15<=l<=15	
Reflections collected	34155	
Independent reflections	5298 [R(int) = 0.0330]	
Completeness to theta = 25.242°	99.0 %	
Refinement method	Full-matrix least-squares on F^2	
Data / restraints / parameters	5298 / 0 / 256	
Goodness-of-fit on F^2	1.055	
Final R indices [I>2sigma(I)]	R1 = 0.0336, wR2 = 0.0841	
R indices (all data)	R1 = 0.0397, wR2 = 0.0882	
Largest diff. peak and hole	0.375 and -0.307 e.$Å^{-3}$	

References

[1] Bruker (**2007**) APEX2 (Version 2.1-4), SAINT (version 7.34A), SADABS (version 2007/4), BrukerAXS Inc, Madison, Wisconsin, USA.

[2] (a) Sheldrick GM. (2008) A short history of SHELX. Acta Cryst. A64, 112-122.
(b) Sheldrick GM. (2015) SHELXT - Integrated space-group and crystal-structure determination. Acta Cryst. A71, 3-8.

[3] (a) Altomare A, Burla C, Camalli M, Cascarano G L, Giacovazzo C, Guagliardi A, Moliterni AGG, Polidori G, Spagna R. **(1999)** SIR97: a new tool for crystal structure determination and refinement *Journal of Applied Cryst*allography, ***32*,** 115-119.

(b) Altomare A, Cascarano G L, Giacovazzo C, Guagliardi A. **(1993)** Completion and refinement of crystal structures with SIR 92. *Journal of Applied Cryst*allography, ***26*,** 343-350.

[4] (a) Sheldrick GM. (1997) SHELXL-97, Program for the Refinement of Crystal Structures. University of Göttingen, Germany.

(b) Sheldrick GM. (2015) Crystal structure refinement with SHELXL. Acta Cryst. C71, 3–8

[5] Waasmaier, D.; Kirfel, A. **(1995)** New Analytical Scattering Factor Functions for Free Atoms and Ions. *Acta Crystallographica* A., ***51***, 416-430.

[6] Farrugia LJ. **(1997)** Ortep-3 for Windows. *Journal of Applied Cryst*allography, ***30*,** 565
